# Synthesis of pyrido-annelated [1,2,4,5]tetrazines, [1,2,4]triazepine, and [1,2,4,5]tetrazepines for anticancer, DFT, and molecular docking studies

**DOI:** 10.1038/s41598-023-32421-x

**Published:** 2023-04-05

**Authors:** Aisha Y. Hassan, Sara N. Shabaan, Samiha A. El-Sebaey, Eman S. Abou-Amra

**Affiliations:** 1 Department of Chemistry, Organic Chemistry, Faculty of Science (Girls), Al-Azhar University, Youssef Abbas Street, Nasr City, Cairo Egypt; 2 Department of Pharmaceutical Organic Chemistry, Faculty of Pharmacy (Girls), Al-Azhar University, Youssef Abbas Street, Nasr City, Cairo Egypt

**Keywords:** Cancer, Organic chemistry

## Abstract

In this strategy, we attempt to design various novel nitrogen-rich heterocycles in one molecule. Green, simple, and efficient aza-annulations of an active, versatile building block, 1-amino-4-methyl-2-oxo-6-phenyl-1,2-dihydropyridine-3-carbonitrile (**1**), with different bifunctional reagents were developed under solvent-free conditions, resulting in the bridgehead tetrazines and azepines (triazepine and tetrazepines). Pyrido[1,2,4,5]tetrazines have been synthesized through two pathways; [3 + 3]- and [5 + 1]-annulations. In addition, pyrido-azepines have been developed by applying [4 + 3]-and [5 + 2]-annulations. This protocol establishes an efficient technique for synthesizing essential biological derivatives of 1,2,4,5-tetrazines, 1,2,4-triazepines, and 1,2,4,5-tetrazepine, tolerating a diverse variety of functionalities without the need for catalysis and fast reaction rates in high yields. The National Cancer Institute (NCI, Bethesda, USA) examined twelve compounds produced at a single high dosage (10^−5^ M). Compounds **4**, **8**, and **9** were discovered to have potent anticancer action against certain cancer cell types. To explain NCI results, the density of states was calculated to conduct a better description of the FMOs. The molecular electrostatic potential maps were created to explain a molecule's chemical reactivity. In silico ADME experiments were performed to better understand their pharmacokinetic characteristics. Finally, the molecular docking investigations on Janus Kinase-2 (PDB ID: **4P7E**) were carried out to study the binding mechanism, binding affinity, and non-bonding contacts.

## Introduction

Heterocyclic compounds containing the pyridine^1^ nucleus show a wide range of interesting biological effects, such as anticancer^2–4^, antioxidant^5,6^, antimicrobial^2,6,7^, and anti-viral^8^. Furthermore, it has been demonstrated that 1,2,4-triazepines have a wide spectrum of therapeutic and biological effects^9–11^.

Moreover, 1,2,4,5-tetrazines are a family of heterocyclic compounds having a diverse variety of biological actions, including anticonvulsant, anti-inflammatory, and antibacterial properties^12^. Many studies have shown that several 1,2,4,5-tetrazine compounds exhibit anticancer activity^13–15^.

Tetrazepines are seven-membered azaheterocyclic molecules with valuable medical and pesticide applications^16^. Tetrazepine derivatives interest chemists and pharmacists, but there are only a few ways to synthesize them. Because of the importance of tetrazepines in pharmaceuticals and their wide variety of uses^17^, great emphasis has lately been dedicated to the synthesis of novel tetrazepines as well as new methodologies.

In light of the above findings, following the pot, atom, and step economy (PASE)^18^ concept aims to reduce the number of steps required to synthesize various fused heterocyclic structures as the vessel must be operated economically in a single reaction vessel without any of the requirement for workup or transitional species separation. We provide a simple method for synthesizing several bioactive nitrogen heterocycles regarding the idea of 'solvent-free', including fused pyrido-1,2,4-triazepines, 1,2,4,5-tetrazines, and 1,2,4,5-tetrazepines.

Computational chemistry is a prominent method for investigating the biological characteristics of recently produced compounds. Density functional theory (DFT) is a quantitative quantum mechanical modelling approach used to explore the thermal and electrical stability of the produced compounds in this work. This study's determined parameters include dipole moment, the highest occupied molecular orbital (HOMO), the lowest unoccupied molecular orbital (LUMO), and the subtraction (HOMO–LUMO) gap energies. The molecular electrostatic potential surfaces (MEPs) were computed using Gauss View to measure their chemical and thermal characteristics.

Molecular docking is a bioinformatics modelling technique that deals with the interaction of two or more molecules to produce a stable adduct. The primary goal of molecular docking is to anticipate the possible binding geometries of produced molecules with a target protein in a three-dimensional structure. As many small ligands with JAK2 inhibitory activity produced therapeutic effects, and visualization of the compounds' greatest binding mode into Janus Kinase-2 (PDB ID: 4P7E) than other proteins, using the computer software Molecular Operating Environment (MOE), the more active compounds were investigated in silico to highlight their likely binding energy and interaction patterns with the active site of JAK2 (PDB ID: **4P7E**). The more potent compounds were then screened for drug-likeness using Lipinski's rule of five and ADME characteristics.

## Experimental

### Chemistry

All melting points were measured and uncorrected in open glass capillaries using an Electrothermal LA 9000 SERIS digital melting point instrument. Bruker high-performance digital FT-NMR spectrometer Avance III was used to scan the ^1^H,^13^C NMR spectra at [(600, 400) & 213] MHz in deuterated dimethyl sulfoxide and chloroform (DMSO-*d*_*6*_ & CDCl_3_) as a solvent. Mass spectra were acquired at 70 eV using a Schimadzu GC/MS-QP-5050A mass spectrometer et al.-Azhar University's regional center for mycology and biotechnology.

### General procedure for the synthesis of compounds (2, 3, 5–7)

Equivalent amounts of **1**^19^ (2.25 g, 10 mmol) and each of [cyanoguanidine (0.84 g, 10 mmol), chloroacetone (0.92 g, 10 mmol), anthranilic acid (1.37 g, 10 mmol), hydrazinecarbonitrile (0.58 mL, 10 mmol), or thiosemicarbazide (0.91 g, 10 mmol)] were fused at 150–170 °C for 3 h. The fused mass was allowed to cool, triturated with ethanol, and then the precipitate was filtered, washed with ethanol, and dried.

#### N-(8-Cyano-7-methyl-5-phenyl-[1,2,4]triazolo[1,5-a]pyridin-2-yl)cyanamide (2)

Yellow crystals are obtained, crystalized from EtOH/DMF (40/60%); yield (79) %, mp.: 228–230 °C; FT‐IR (KBr, ν, cm^−1^): 3190(NH), 3003(CH-Ar), 2980 (CH-aliph.), 2230, 2150 (2(C≡N), 1608(C=N); ^1^H NMR (400 MHz, DMSO‐*d*_6_) δ(ppm): 2.51 (s, 3H, CH_3_), 3.80(br., 1H, NH–C≡N, exchangeable with D_2_O), 7.29–7.56 (m, 5H, Ar–H), 8.09 (s, 1H, CH-pyridine). ^13^C NMR (213 MHz, CDCl_3_) δ(ppm): 29.24(CH_3_),111.23 (pyridine C–C≡N), 118.93 (C≡N),125.11 (CH-pyridine), 128.87, 129.73, 138.00 (phenyl C), 146.18 (2C=N), 156.37 (pyridine C–CH_3_), 170.61 (pyridine C–Ph). MS (m/z, %): 274.08 (M^+·^, 31); 272.08 (M^+·^–2H, 29); 141.84 (100). Anal. Calcd. for C_15_H_10_N_6_ (274.29); C, 65.68; H, 3.67; N, 30.64. Found C, 65.75; H, 3.77; N, 30.76.

#### 4-Methyl-2-oxo-1-((2-oxopropyl)amino)-6-phenyl-1,2-dihydropyridine-3-carbonitrile (3)

Orange crystals are obtained, crystallized from EtOH; yield (85) %, mp.: 115–118 °C; FT‐IR (KBr, ν, cm^−1^): 3400 (OH tautomer), 3250(NH), 3000, 2890 (CH), 2220 (C≡N), 1650, 1630 (2C=O), 1590 (C=N); ^1^H NMR: (400 MHz, DMSO‐*d*_6_) δ(ppm): 2.24, 2.29 (2s, 6H, CH_3_ & CH_3_-tautomer); 2.19 (2s, 6H, COCH_3_ & COCH_3_—tautomer); 3.80 (s, 2H, CH_2_); 4.17, 4.28 (2s, 2H, NH & NH—tautomer, exchangeable with D_2_O); 5.05(s, 1H, =CH(tautomer)); 6.50, 6.55 (2s, 2H, CH-pyridine & CH-tautomer); 7.30–7.73 (m, 10H, Ar–H), 10.51 (s, 1H, OH(tautomer), exchangeable with D_2_O). MS (m/z, %): 281.46(M^+^, 16); 138.67(100). Anal. Calcd. for C_16_H_15_N_3_O_2_ (281.32); C, 68.31; H, 5.37; N, 14.94. Found C, 68.39; H, 5.42; N, 15.05.

#### 3,9-Dimethyl-2,7-diphenyl-2,5-dihydropyrido[1,2-b][1,2,4,5]tetrazepine-10-carbonitrile (4)

Equivalent amounts of **3** (2.81 g, 10 mmol) and phenylhydrazine (1.08 mL, 10 mmol) were fused at 150–170 °C for 2.5 h. The resultant solid was triturated with ethanol, filtered, washed by ethanol, dried, and crystallized from EtOH.

Brown crystals are obtained; yield (72) %, mp.: 241–243 °C; FT‐IR (KBr, ν, cm^−1^): 3170 (NH), 3020 (CH-Ar), 2870 (CH-aliph.), 2150 (C≡N); 1600 (C=N); ^1^H NMR (400 MHz, DMSO‐*d*_6_) δ(ppm): 2.22, 2.24 (2s, 6H, 2CH_3_), 2.65(s, 1H, NH-tetrazepine, exchangeable with D_2_O), 4.18 (s,1H, CH-tetrazepine), 7.25–7.58 (m, 10H, Ar–H), 7.73 (s, 1H, CH-pyridine). ^13^C NMR (213 MHz, CDCl_3_) δ (ppm): 15.62 (CH_3_), 21.20 (CH_3_), 95.07(CH-pyridine), 104.75 (CH-tetrazepine),118.81 (C≡N), 119.06, 119.24, 122.15, 122.37, 126.07, 126.25, 129.00, 130.15, 131.73 (phenyl C). MS (m/z, %): 353.66 (M^+·^, 9); 352.56(M^+·^–H, 23); 328.22(100). Anal. Calcd. for C_22_H_19_N_5_ (353.43); C, 74.77; H, 5.42; N, 19.82. Found, C, 74.80; H, 5.48; N, 19.78.

#### 2-Methyl-7-oxo-4-phenyl-6,7-dihydrobenzo[e]pyrido[1,2-b][1,2,4]triazepine-1-carbonitrile (5)

Brown powder are obtained, crystallized from EtOH; yield (78) %, mp.: 189–191 °C; FT‐IR (KBr, ν, cm^−1^): 3420 (NH/OH), 3005 (CH–Ar), 2890 (CH–aliph.), 2226 (C≡N), 1625 (C=O); 1605 (C=N); ^1^H NMR: (400 MHz, CDCl_3_) δ(ppm): 2.20 (s, 3H, CH_3_), 7.17–7.85 (m, 9H, Ar–H), 7.40 (s, 1H, CH–pyridine), 10.24(s, 1H, NH, exchangeable with D2O). ^13^C NMR (213 MHz, DMSO-*d*_*6*_) δ (ppm): 24.31 (CH_3_), 95.63 (CH–pyridine), 117.98 (C≡N), 122.03, 122.36, 124.75, 128.50, 128.68, 129.42, 138.62, 141.79, 143.40 (phenyl C), 148.17 (C=N), 162.56 (C=O). MS (m/z, %): 323.51(M^+·^–3H, 9); 94.24 (100). Anal. Calcd. for C_20_H_14_N_4_O (326.36); calc. C, 73.61; H, 4.32; N, 17.17. Found C, 73.64; H, 4.30; N, 17.15.

#### 3-Amino-8-methyl-6-phenyl-4H-pyrido[1,2-b][1,2,4,5]tetrazine-9-carbonitrile (6)

VYellow crystals are obtained, crystallized from EtOH; yield (67) %, mp.: 187–189 °C; FT‐IR (KBr, ν, cm^−1^): 3380, 3250, 3120 (NH_2_, NH), 3010 (CH–Ar), 2950 (CH–aliph.), 2227 (C≡N), 1611 (C=N). Anal. Calcd. for C_14_H_12_N_6_ (264.29); C, 63.62; H, 4.58; N, 31.80. Found C, 63.72; H, 4.52; N, 31.73.

#### 3-Amino-8-methyl-6-phenyl-2H-pyrido[1,2-b][1,2,4,5]tetrazine-9-carbonitrile (7)

Yellow crystals are obtained, crystallized from EtOH; yield (89) %, mp.: 202–204 °C; FT‐IR (KBr, ν, cm^−1^): 3300, 3220 (NH_2_, NH), 3000 (CH-Ar), 2900 (CH-aliph.), 2170 (C≡N), 1610 (C=N). ^1^H NMR (400 MHz, DMSO‐*d*_6_) δ (ppm): 2.25 (s, 6H, CH_3_, CH_3_(tautomer)), 6.43(s, 2H, NH_2_, exchangeable with D_2_O), 7.25–7.40 (m, 5H, Ar–H), 7.73, 7.75 (2 s, 2H, CH-pyridine & CH-pyridine (tautomer)), 8.07 (s, 1H, NH-tetrazine (tautomer), exchangeable with D_2_O), 12.38, 12.60 (2s, 2H, =NH, NH-tetrazine, exchangeable with D_2_O). ^13^C NMR (213 MHz, DMSO‐*d*_6_) δ(ppm): 15.34, 15.63 (CH_3_, CH_3_ (tautomer)), 89.35, 94.23 (CH–pyridine, CH–pyridine (tautomer)), 117.36 (C≡N), 120.68, 121.49, 125.36, 129.28, 139.00, 140.40 (phenyl C). MS (m/z, %): 264.80 (M^+·^, 9); 262.47 (M^+·^–2H, 25); 50.27 (100). Anal. Calcd. for C_14_H_12_N_6_ (264.29); C, 63.62; H, 4.58; N, 31.80. Found C, 63.72; H, 4.52; N, 31.89.

### General procedure for the synthesis of compounds (8–10)

Equivalent amounts of **7** (2.64 g, 10 mmol) and each of [(diethylmalonate (1.58 g, 10 mmol), phenacyl bromide (1.97 g, 10 mmol) or diethyl oxalate (1.47 mL, 10 mmol)] were fused at 150–180 °C for 3 h. The produced mass was allowed to cool, triturated with ethanol, and then the precipitate was filtered, washed with ethanol, dried, and crystallized from EtOH.

#### 8-Methyl-2,4-dioxo-10-phenyl-1,2,3,4-tetrahydropyrido[1,2-b]pyrimido[1,2-e][1,2,4,5]tetrazine-7-carbonitrile (8)

Orange crystals are obtained; yield (78) %, mp.: 250–252 °C; FT‐IR (KBr, ν, cm^−1^): 3420 (OH tautomer), 3380 (NH), 3000 (CH-Ar), 2970(CH-aliph.), 2225 (C≡N), 1750, 1670 (2C=O), 1600 (C=N). ^1^H NMR (400 MHz, DMSO‐*d*_6_) δ(ppm): 2.14 (s, 3H, CH_3_), 3.38 (s, 2H, CH_2_-pyrimidindione), 7.16–7.45 (m, 5H, Ar–H), 8.05, 8.07 (2s, 2H, CH-pyridine & CH-pyridine (tautomer), 8.33, 8.36 (2s, 2H, NH-pyrimidindione & NH-pyrimidindione (tautomer), exchangeable with D_2_O), 12.08, 12.09 (br.,2H,2OH(tautomer), exchangeable with D_2_O). ^13^C NMR (213 MHz, DMSO‐*d*_6_) δ (ppm): 17.13 (CH_3_), 43.47 (CH_2_), 88.05 (CH-pyridine), 118.42(C≡N), 121.01, 124.89, 125.45, 129.34, 138.59, 139.41 (phenyl C), 160.74, 163.55 (2C=O). MS (m/z, %): 332.05 (M^+·^, 25); 309.49(100). Anal. Calcd. for C_17_H_12_N_6_O2 (332.32); C, 61.44; H, 3.64; N, 25.29. Found, C, 61.35; H, 3.52; N, 25.21.

#### 7-Methyl-2,9-diphenyl-3H-imidazo[1,2-b]pyrido[1,2-e][1,2,4,5]tetrazine-6-carbonitrile (9)

Orange crystals are obtained; yield (85) %, mp.: 258–260 °C; FT‐IR (KBr, ν, cm^−1^): 3100 (CH-Ar), 2970 (CH-aliph.), 2215 (C≡N), 1609(C=N). ^1^H NMR (400 MHz, DMSO‐*d*_6_) δ(ppm): 2.08 (s, 3H, CH_3_), 4.59 (s, 2H, CH_2_-imidazole), 7.28–7.76 (m, 10H, Ar–H), 7.40 (s,1H, CH-pyridine). ^13^C NMR (213 MHz, CDCl_3_) δ(ppm): 22.94(CH_3_), 52.29 (CH_2_-imidazole), 99.54 (CH-pyridine), 119.40 (C≡N), 120.82, 121.15, 125.88, 126.60, 128.84, 129.41, 137.42, 138.37 (phenyl C). MS (m/z, %): 364.39 (M^+·^, 28); 198.96 (100). Anal. Calcd. for C_22_H_16_N_6_ (364.41); C, 72.51; H, 4.43; N, 23.06. Found C, 72.45; H, 4.49; N, 23.02.

#### *7-Methyl-2,3-dioxo-9-phenyl-2,3-dihydro-1H-imidazo[1,2-b]pyrido[1,2-e]*[1, 2, 4, 5]* tetrazine-6-carbonitrile (10)*

Orange crystals are obtained; yield (74) %, mp.: 143–145 °C; FT‐IR (KBr, ν, cm^−1^): 3300 (NH), 3000 (CH-Ar), 2870 (CH-aliph.), 2220 (C≡N), 1750, 1650 (2C=O), 1600(C=N). ^1^H NMR (400 MHz, DMSO‐*d*_6_) δ (ppm): 2.25 (s, 3H, CH_3_), 7.25–7.73 (m, 5H, Ar–H), 7.75 (s, 1H, CH-pyridine), 8.63 (s, 1H, NH-imidazole, exchangeable with D_2_O). ^13^C NMR (213 MHz, DMSO‐*d*_6_) δ (ppm): 20.81 (CH_3_), 87.75 (CH-pyridine), 116.70 (C≡N), 121.36, 125.12, 129.33, 137.56, 138.40 (phenyl C), 175.17, 175.70 (2C = O). MS (m/z, %): 318.44 (M^+·^, 23); 248.85 (100). Anal. Calcd. for C_16_H_10_N_6_O_2_ (318.30); C, 60.38; H, 3.17; N, 26.40. Found C, 60.46; H, 3.25; N, 26.49.

#### 1-Amino-2-hydrazono-4-methyl-6-phenyl-1,2-dihydropyridine-3-carbonitrile (11)

Equivalent amounts of **1**^19^ (2.25 g, 10 mmol) and hydrazine hydrate (0.4 mL, 10 mmol) were fused at 120–140 °C for 3 h. The reaction mixture was allowed to cool, triturated with ethanol, and then the precipitate was filtered, washed with ethanol, dried, and crystallized from EtOH.

Orange powder are obtained; yield (84) %, mp.: 176–178 °C; FT‐IR (KBr, ν, cm^−1^): 3352, 3315(NH_2_), 3003 (CH-Ar), 2990 (CH-aliph.), 2250 (C≡N), 1608 (C=N). ^1^H NMR (600 MHz, DMSO‐*d*_6_) δ (ppm): 2.11 (s, 3H, CH_3_), 4.50(s,2H, NH_2,_ exchangeable with D_2_O), 5.49 (s, 2H, =N–NH_2_, exchangeable with D_2_O), 7.28 (s, 1H, CH-pyridine), 6.99–7.29 (m, 5H, Ar–H). ^13^C NMR (213 MHz, DMSO‐*d*_6_) δ (ppm): 19.01(CH_3_), 90.34(CH-pyridine), 118.11(CN), 120.44, 123.72, 125.07, 130.07, 134.61 (phenyl C), 146.49 (C=N). MS (m/z, %): 239.72(M^+·^, 18); 71.42(100). Anal. Calcd. for C_13_H_13_N_5_ (239.12); C, 65.25; H, 5.48; N, 29.27. Found C, 65.20; H, 5.53; N, 29.34.

### General procedure for the synthesis of compounds (12–16)

Equivalent amounts of **11** (2.39 g, 10 mmol) and each of [phenylisothiocyanate (1.35 mL, 10 mmol), chloroacetone (0.92 mL, 10 mmol), phenacyl bromide (1.97 g, 10 mmol), diethyl oxalate (1.47 mL, 10 mmol) or 3,4-dimethoxybenzaldehyde (1.66 g, 10 mmol)] were fused at 140–160 °C for 2 h. The fused mass was allowed to cool, triturated with ethanol, and then the precipitate was filtered, washed with ethanol, and dried.

#### 8-Methyl-6-phenyl-3-(phenylamino)-4H-pyrido[1,2-b][1,2,4,5]tetrazine-9-carbonitrile (12)

Brown crystals are obtained, crystallized from EtOH/DMF (70–30%); yield (71) %, mp.: 223–225 °C; FT‐IR (KBr, ν, cm^−1^): 3420(NH), 3010(CH-Ar), 2980 (CH-aliph.), 2230 (C≡N), 1619 (C=N); ^1^H NMR: (600 MHz, CDCl_3_) δ (ppm): 2.45 (s, 3H, CH_3_), 4.03 (s, 1H, NH tetrazine), 6.77–7.49 (m, 10H, CH-aromatic), 7.82 (s, 1H, CH-pyridine), 7.86 (s, 1H, NH–Ph), ^13^C NMR (213 MHz, DMSO‐*d*_6_) δ (ppm): 24.31(CH_3_), 95.63, 96.4 (2CH-pyridine), 117.84, 117.99 (2C≡N), 118.27, 118.80, 122.03, 122.36, 124.75, 128.50, 129.42, 138.62, 141.17(phenyl C), 152.56, 154.74 (2 (C=N)-tetrazine). MS (m/z, %): 340.16(M^+·^, 13); 339.17(M^+·^–H, 43); 148.56(100). Anal. Calcd. for C_20_H_16_N_6_ (340.14); C, 70.57; H, 4.74; N, 24.69. Found C, 70.63; H, 4.82; N, 24.75.

#### 3,9-Dimethyl-7-phenyl-4,5-dihydropyrido[1,2-b][1,2,4,5]tetrazepine-10-carbonitrile (13)

Yellow crystals are obtained, crystallized from EtOH; yield (83) %, mp.: 210–212 °C; FT‐IR (KBr, ν, cm^−1^): 3350(NH), 3009(CH-Ar), 2950(CH-aliph.), 2225 (C≡N), 1590 (C=N). ^1^H NMR(600 MHz, CDCl_3_) δ (ppm): 2.20 (s, 6H, 2CH_3_)_,_ 3.44 (s, 2H, CH_2-_tetrazepine), 4.20 (s, 1H, NH-tetrazepine, exchangeable with D_2_O), 7.18–7.40 (m, 5H, Ar–H), 7.85(s, 1H, CH-pyridine), ^13^C NMR (213 MHz, DMSO‐*d*_6_) δ(ppm): 21.36, 22.40 (2CH_3_), 65.41 (CH_2_-tetrazepine), 87.72 (CH-pyridine), 114.20 (CH-tetrazepine), 118.20 (C≡N), 120.68, 121.48, 125.36, 129.28, 139.00 (phenyl C), 152.56, 154.74 (2 (C=N)-tetrazepine). MS (m/z, %): 277.47(M^+·^, 34); 65.36 (100). Anal. Calcd. for C_16_H_15_N_5_ (277.33); C, 69.29; H, 5.45; N, 25.25. Found C, 69.35; H, 5.38; N, 25.18.

#### 9-Methyl-3,7-diphenyl-2,5-dihydropyrido[1,2-b][1,2,4,5]tetrazepine-10-carbonitrile (14)

Orange powder is obtained, crystallized from EtOH; yield (86) %, mp.: 230–232 °C; FT‐IR (KBr, ν, cm^−1^): 3330 (NH), 3010 (CH-Ar), 2960 (CH-aliph.), 2220 (C≡N), 1605 (C=N). ^1^H NMR (400 MHz, DMSO‐*d*_6_) δ(ppm): 2.23 (s, 3H, CH_3_), 4.51(s, 2H, CH & NH-tetrazepine), 7.25–7.70 (m,10H, Ar–H), 7.76 (s, 1H, CH-pyridine), 8.10 (s, 1H, NH-tetrazepine). ^13^C NMR (213 MHz, DMSO‐*d*_6_) δ(ppm): 24.21(CH_3_), 86.41 (CH-pyridine), 104.85 (CH-tetrazepine), 117.84 (C≡N), 122.03, 122.36, 124.75, 128.57, 128.68, 129.42, 138.62 (phenyl C), 149.17 (C=N-tetrazepine). MS (m/z, %): 339.67 (M^+·^, 4); 336.80 (M^+·^–3H, 21); 78.29 (100). Anal. Calcd. for C_21_H_17_N_5_ (339.40); C, 74.32; H, 5.05; N, 20.63. Found C, 74.29; H, 5.01; N, 20.69.

#### 9-Methyl-3,4-dioxo-7-phenyl-2,3,4,5-tetrahydropyrido[1,2-b][1,2,4,5]tetrazepine-10-carbonitrile (15)

Faint yellow crystals are obtained, crystallized from EtOH; yield (86) %, mp.: 200–202 °C; FT‐IR (KBr, ν, cm^−1^): 3450, 3335 (2NH), 3009 (CH-Ar), 2890 (CH-aliph.), 2220 (C≡N), 1650, 1630 (2C=O), 1600 (C=N). ^1^H NMR (400 MHz, DMSO‐*d*_6_) δ (ppm): 2.12 (s, 3H, CH_3_), 7.70 (s, 1H, CH-pyridine) 7.64–7.76 (m, 5H, Ar–H), 8.44, 8.56 (2s, 2H, 2NH-tetrazepine. ^13^C NMR (213 MHz, DMSO‐*d*_6_) δ (ppm): 21.53 (CH_3_), 82.71 (CH_-_pyridine), 118.22 (C≡N), 120.51, 123.74, 124.17, 127.68, 129.24, 129.41 (phenyl C), 147.74 (C=N-tetrazepine), 151.08, 152.51 (2C=O). MS (m/z, %): 293.06 (M^+·^, 20); 55.68 (100). Anal.Calcd. for C_15_H_11_N_5_O_2_ (293.29); C, 61.43; H, 3.78; N, 23.88. Found, C, 61.38; H, 3.72; N, 23.80.

#### 1-((3,4-Dimethoxybenzylidene)amino)-2-hydrazono-4-methyl-6-phenyl-1,2-dihydro- pyridine-3-carbonitrile (16)

Orange crystals are obtained; crystallized from EtOH; yield (88) %, mp.: 246–248 °C; FT‐IR (KBr, ν, cm^−1^): 3450, 3340 (NH_2_), 3010 (CH-Ar), 2950 (CH-aliph.), 2225 (C≡N), 1600(C=N). ^1^H NMR (600 MHz, DMSO‐*d*_6_) δ (ppm): 2.15 (s, 3H, CH_3_), 3.44 (s, 3H, OCH_3_), 3.70 (s, 3H, OCH_3_), 5.36 (s, 2H, NH_2_, exchangeable with D_2_O), 7.07–7.42 (m, 8H, Ar–H), 7.40 (s, 1H, CH-pyridine), 7.69 (s, 1H, CH=N–). Anal. Calcd. for C_22_H_21_N_5_O_2_ (387.44); C, 68.20; H, 5.46; N, 18.08. Found, C, 68.14; H, 5.54; N, 18.02.

### Biological activities

#### NCI anticancer activity screening

The preliminary anticancer screening for novel fused pyridine derivatives (**2**–**16**) was performed in the Developmental Therapy Program of the National Cancer Institute (NCI) in the United States. In accordance with the NCI, Bethesda, Drug Evaluation Branch procedure (http://dtp.nci.nih.gov), the twelve derivatives **2**, **4**, **5**, **7**–**10,** and **12**–**16** were chosen and tested for initial in vitro one dose anticancer testing at 10^–5^ M concentration against full NCI 60 cell line screens indicating nine various types of cancer comprising renal cancer, leukaemia, melanoma, prostate cancer, non-small cell lung cancer, ovarian cancer, CNS cancer, breast cancer. The results of each tested compound (**2**, **4**, **5**, **7**–**10**, and **12**–**16**) were provided as a mean chart of the growth percentage of the treated cells as compared to the control, which reveals both inhibitory values (between 0 and 100) and toxicity values (less than 0). The COMPARE tool was used to examine the single-dose evaluation findings of all chosen compounds against sixty cancer cell lines. As previously stated^20^, every molecule supplied for the NCI-60 Cell screen is examined at a single high dosage (10^−5^ M).

#### Protein preparation and molecular docking work

The docking investigation was carried out using the computer software Molecular Operating Environment (MOE) version 2015.10^21^, Chemical Computing Group Inc., Montreal, Quebec, Canada. The docking technique was carried out as previously reported^22^. The 3-dimensional (3D) structure of Janus Kinase (Jak2) with PDB ID: **4P7E** was obtained through the link (https://www.rcsb.org/structure/4P7E) on the Protein Data Bank site. Using the program's default parameters, the co-crystallized ligand was re-docked in its original protein structure. Interactions of the amino acids, affinities by bond strength, and hydrogen bond lengths were illustrated in Table 1.

**Table 1:** In vitro anticancer screening results of compounds **2, 4, 5, (7–10) and (12–16)** against sixty human tumour cell lines with single dose assay (10^–5^ M concentration). Data was provided as cell growth inhibition percentage.

Subpanel/tumour cell lines	Compounds^a^											
	**2**	**4**	**5**	**7**	**8**	**9**	**10**	**12**	**13**	**14**	**15**	**16**
Leukemia												
CCRF-CEM	**–**	**–**	**–**	**–**	**–**	**–**	**–**	**–**	**–**	**–**	**–**	**–**
HL-60(TB)	**–**	**–**	**–**	**–**	**–**	**–**	**–**	**–**	**–**	**–**	**–**	**–**
K-562	**–**	12.50	**–**	19.06	11.47	**–**	**–**	**–**	**–**	**–**	**–**	**–**
MOLT-4	**–**	**–**	**–**	**–**	**–**	**–**	**–**	**–**	**–**	**–**	**–**	**–**
RPMI-8226	**–**	**22.45**	13.43	11.43	**29.42**	**51.58**	**–**	**–**	**–**	**–**	**–**	**–**
SR	**–**	**–**	**–**	9.30	**–**	**–**	**–**	**–**	**–**	**–**	**–**	**–**
Non-small cell lung cancer												
A549/ATCC	**–**	**–**	**–**	**–**	**–**	**–**	**–**	**–**	**–**	**–**	**–**	**–**
EKVX	**–**	**–**	**–**	**–**	**–**	**–**	16.39	**–**	**–**	**–**	**–**	**–**
HOP-62	16.88	**23.38**	12.88	**30.19**	**29.39**	**20.17**	**22.28**	18.57	13.10	14.43	16.16	**22.80**
HOP-92	NT	NT	NT	NT	NT	NT	NT	NT	NT	NT	NT	NT
NCI-H226	**–**	**–**	**–**	**–**	**–**	11.58	**–**	**–**	**–**	**–**	**–**	**–**
NCI-H23	**–**	**–**	**–**	**–**	**–**	**–**	**–**	**–**	**–**	**–**	**–**	**–**
NCI-H322M	**–**	**–**	**–**	8.09	**–**	**–**	**–**	**–**	**–**	**–**	**–**	**–**
NCI-H460	**–**	**–**	**–**	**–**	**–**	**–**	**–**	**–**	**–**	**–**	**–**	**–**
NCI-H522	10.14	19.05	13.32	16.19	**24.14**	12.55	8.83	10.63	**–**	**–**	**–**	**–**
Colon cancer												
COLO 205	**–**	**–**	**–**	**–**	**–**	**–**	**–**	**–**	**–**	**–**	**–**	**–**
HCC-2998	**–**	**–**	**–**	**–**	**–**	**–**	**–**	**–**	**–**	**–**	**–**	**–**
HCT-116	**–**	9.85	**–**	**–**	14.71	**77.94**	**–**	**–**	**–**	**–**	**–**	**–**
HCT-15	**–**	**–**	**–**	**–**	**–**	**–**	**–**	**–**	**–**	**–**	**–**	**–**
HT29	**–**	**–**	**–**	**–**	**–**	**–**	**–**	**–**	**–**	**–**	**–**	**–**
KM12	**–**	**–**	**–**	**–**	10.42	**-**	**–**	**–**	**–**	**–**	**–**	**–**
SW-620	**–**	**–**	**–**	**–**	**–**	8.07	**–**	**–**	**–**	**–**	**–**	**–**
CNS cancer												
SF-268	**–**	**–**	**–**	**–**	**–**	**–**	**–**	**–**	**–**	**–**	**–**	**–**
SF-295	**–**	**–**	**–**	**–**	14.91	9.57	**–**	**–**	**–**	**–**	**–**	**–**
SF-539	**–**	**–**	**–**	**–**	**–**	**–**	**–**	**–**	**–**	**–**	**–**	**–**
SNB-19	**–**	10.40	8.87	8.94	14.40	12.79	**–**	**–**	**–**	**–**	**–**	**–**
SNB-75	NT	NT	NT	NT	NT	NT	NT	NT	NT	NT	NT	NT
U251	**–**	**–**	**–**	**–**	**–**	9.37	**–**	**–**	**–**	**–**	**–**	**–**
Melanoma												
LOX IMVI	**–**	**–**	**–**	8.68	**–**	8.76	**–**	**–**	**–**	**–**	**–**	**–**
MALME-3M	**–**	**–**	9.41	**–**	14.76	**–**	16.30	**–**	**–**	**–**	**–**	8.69
M14	**–**	**–**	**–**	**–**	**–**	**–**	**–**	**–**	**–**	**–**	**–**	**–**
MDA-MB-435	**–**	**–**	**–**	**–**	**–**	**–**	**–**	**–**	**–**	**–**	**–**	**–**
SK-MEL-2	**–**	**–**	**–**	**–**	**–**	**–**	**–**	**–**	**–**	**–**	**–**	**–**
SK-MEL-28	**–**	**–**	**–**	**–**	**–**	**–**	**–**	**–**	**–**	**–**	**–**	**–**
SK-MEL-5	**–**	**–**	**–**	**–**	**–**	**–**	**–**	**–**	**–**	**–**	**–**	**–**
UACC-257	**–**	**–**	**–**	**–**	9.97	**–**	**–**	**–**	**–**	**–**	**–**	**–**
UACC-62	**–**	**21.71**	**–**	**22.82**	**28.57**	19.56	12.73	**–**	**–**	**–**	**–**	9.64
Ovarian cancer												
IGROV1	**–**	15.09	**–**	**–**	**–**	10.62	**–**	**–**	**–**	**–**	**–**	**–**
OVCAR-3	**–**	**–**	**–**	**–**	**–**	**–**	**–**	**–**	**–**	**–**	**–**	**–**
OVCAR-4	**–**	9.96	**–**	**–**	13.10	**–**	**–**	**–**	**–**	**–**	**–**	**–**
OVCAR-5	**–**	**–**	**–**	**–**	**–**	**–**	**–**	**–**	**–**	**–**	**–**	**–**
OVCAR-8	**–**	**–**	**–**	**–**	**–**	**–**	**–**	**–**	**–**	**–**	**–**	**–**
NCI/ADR-RES	**–**	**–**	**–**	**–**	**–**	**–**	**–**	**–**	**–**	**–**	**–**	**–**
SK-OV-3	**–**	8.53	**–**	11.98	**36.25**	12.68	13.42	10.36	**–**	**–**	**–**	17.88
Renal cancer												
786-0	**–**	**–**	**–**	**–**	**–**	**–**	**–**	**–**	**–**	**–**	**–**	**–**
A498	**–**	**–**	**–**	**–**	**–**	**–**	**–**	**–**	**–**	**–**	**–**	**–**
ACHN	**–**	8.16	**–**	**–**	**–**	**–**	**–**	**–**	**–**	**–**	**–**	**–**
CAKI-1	**–**	16.21	**–**	16.91	16.39	16.77	13.41	**–**	**–**	**–**	**–**	15.57
RXF 393	**–**	**–**	**–**	**–**	**–**	**–**	**–**	**–**	**–**	**–**	**–**	**–**
SN12C	**–**	**–**	**–**	**–**	**–**	**–**	**–**	**–**	**–**	**–**	**–**	**–**
TK-10	13.75	**–**	**–**	**–**	**–**	**–**	**–**	**–**	**–**	**–**	9.11	**-**
UO-31	9.31	**32.07**	**24.74**	**28.36**	**24.10**	**36.84**	**34.48**	**25.30**	10.78	8.76	9.83	**27.71**
Prostate cancer												
PC-3	**–**	8.83	**–**	**21.02**	**23.28**	9.91	**–**	**–**	**–**	**–**	**–**	18.14
DU-145	**–**	**–**	**–**	**–**	**–**	**–**	**–**	**–**	**–**	**–**	**–**	**–**
Breast cancer												
MCF7	NT	NT	NT	NT	NT	NT	NT	NT	NT	NT	NT	NT
MDA-MB- 231/ATCC	**–**	13.08	**–**	15.43	13.78	15.35	17.01	**–**	**–**	**–**	**–**	10.11
HS 578 T	**–**	**–**	**–**	**–**	8.14	9.27	13.16	**–**	**–**	**–**	**–**	**–**
BT-549	**–**	**–**	**–**	**–**	**–**	**–**	**–**	**–**	**–**	**–**	**–**	11.22
T-47D	**–**	**32.30**	9.75	**28.31**	18.61	19.37	12.09	**–**	**–**	**–**	**–**	15.02
MDA-MB-468	**–**	**–**	**–**	**–**	**–**	**–**	**–**	**–**	**–**	**–**	**–**	**–**

^a^Only GI % higher than 8% are shown. *NT* not tested.

Significant values are in [bold].

#### Designing and optimization of more potent fused pyridine derivatives

Molecular orbital and electrostatic characteristics are frequently calculated using quantum mechanical techniques. The Gaussian 09 software program was used to complete the calculations, which used density functional theory (DFT)^23^. DFT with Beck's (B)^24^ three-parameter hybrid models and Lee, Yang, and Parr's (LYP)^25^ association function under 6-31G (d,p) basis set was used to optimize and forecast the molecular orbital features of the more active fused pyridine derivatives (**4**, **8**, and **9**). At the same level of theory, the highest occupied molecular orbital (HOMO) and the lowest unoccupied molecular orbital (LUMO) were counted as frontier molecular orbital characteristics. The HOMO–LUMO energy gap was computed for each of the more powerful derivatives.

#### In silico physicochemical properties, drug-likeness, and pharmacokinetics profiles of most active compounds 4, 8, and 9 compared to the reference 2HB

Drug development is incomplete unless the pharmacokinetic parameters of drug candidates are evaluated during the primary screening stage because some drug candidates do not act therapeutically due to insufficient pharmacokinetic characteristics. This process may result in better hits and less late-stage abrasion of drug candidates. To identify the physicochemical properties, drug-likeness, and ADME profile, "absorption, distribution, metabolism, and excretion" were calculated hypothetically based on SwissADME online software [www.SwissADME.ch].

## Discussion of the results

### Chemistry

As reported, the cyclocondensation of 2-cyanoacetohydrazide with benzoyl acetone in a slightly alkaline medium afforded the dihydropyridine-3-carbonitrile (**1**)^19^ as the primary entry material for the synthesis of bridgehead pyrido[1,2,4,5]tetrazine and pyrido-azepines with one ring joint nitrogen atom.

**Figure 1:**
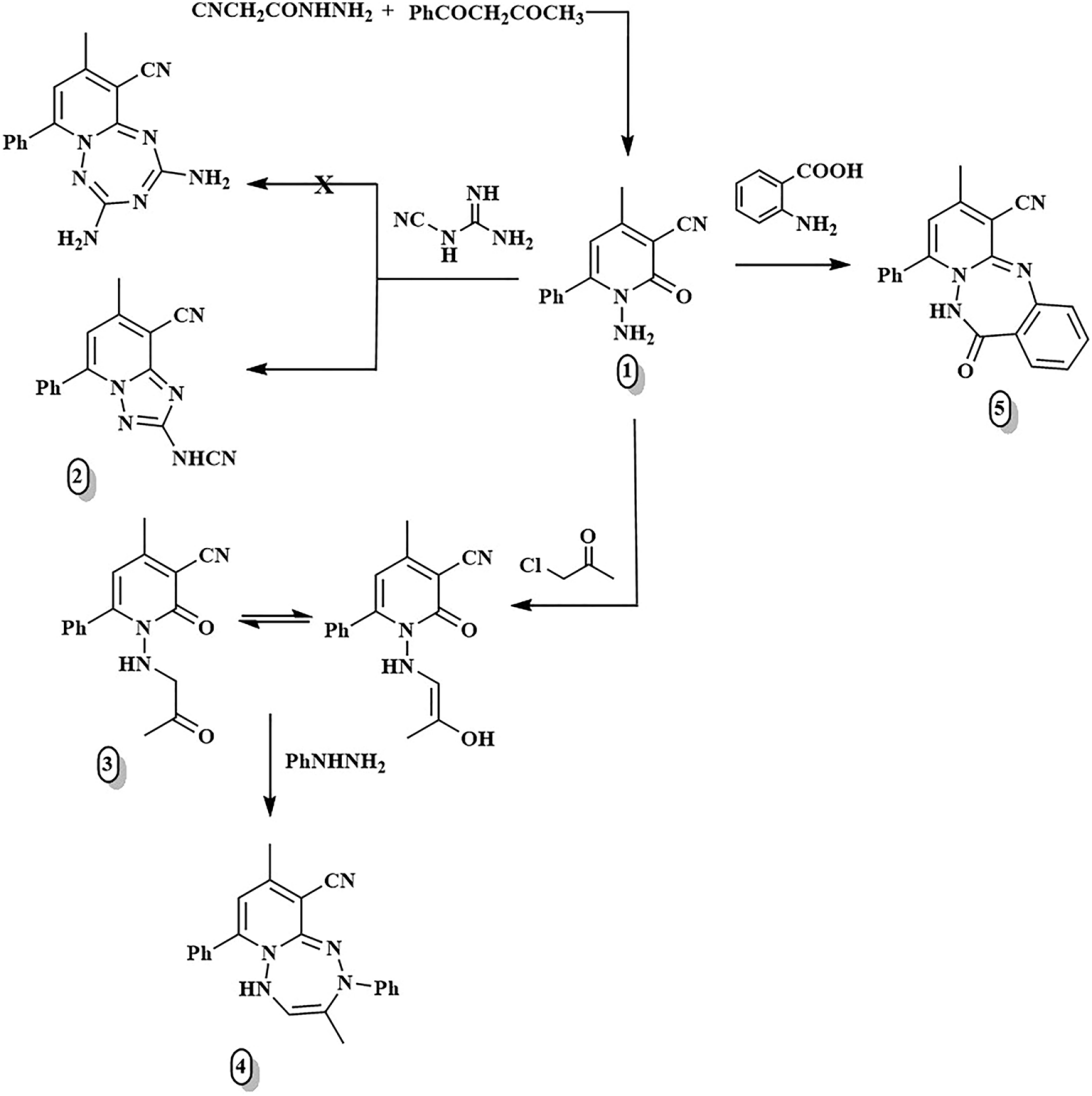
Synthesis of compounds **2**–**5.**

The treatment of building block **1**^19^ with cyanoguanidine yielded the fused triazolopyridine **2** in good yield, rather than the predictable 2,4-diamino-9-methyl-7-phenylpyrido[1,2-*b*][1,2,4,6]tetrazepine-10-carbonitrile (Fig. 1). Compound **2** is produced due to the elimination of the water molecule, followed by intramolecular cyclization by removing the NH_3_ molecule. The appearance of an NH proton signal at 3.80 ppm exchangeable with D_2_O in the ^1^H-NMR spectrum, and a new carbon signal corresponding to the cyano group at 118.93 ppm in the ^13^C NMR spectrum, affirmed the structure of compound **2**.

Additionally, substrate **1**^19^ was alkylated with chloroacetone, furnishing the open-chain product **3**, which was further subjected to cyclocondensation upon fusion with phenylhydrazine to form the cyclized [1,2,4,5]tetrazepine analogue** 4**. The structures of these compounds have been verified by their spectral data (See the Supplementary file).

The fused heterocyclic system (6:7:6) with a single ring connection atom of nitrogen was synthesized through the fusion of the key compound **1**^19^ with the binucleophilic anthranilic acid at 140–160 °C, affording the biologically active benzo[*e*]pyrido[1,2,4]triazepine derivative **5** (Fig. 1).

**Figure 2:**
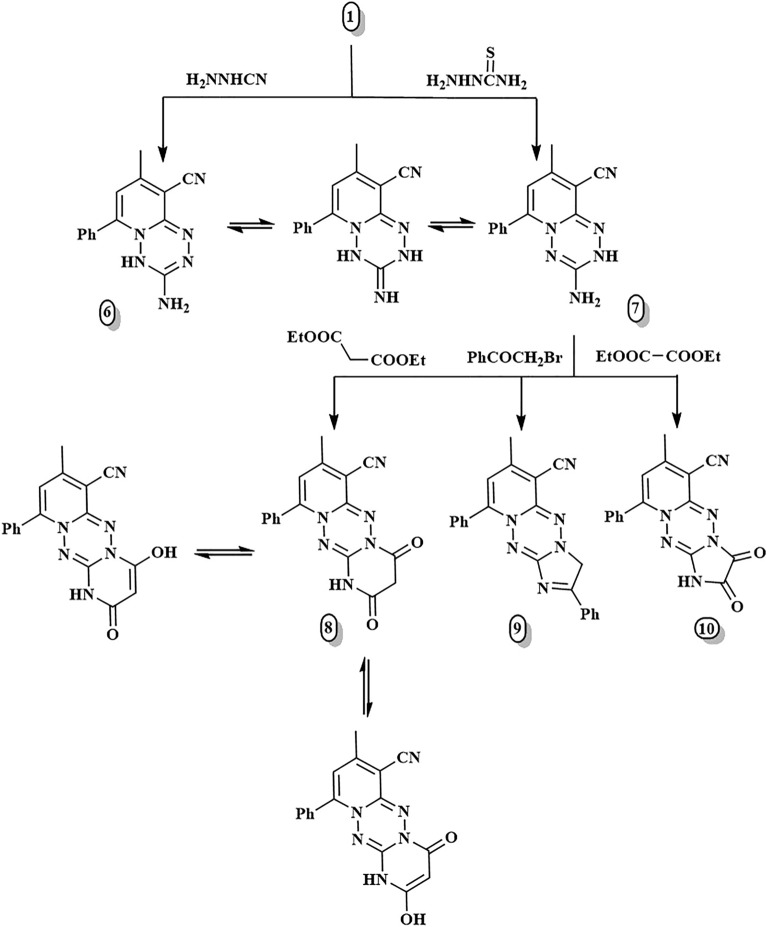
Synthesis of compounds **6**–**10**.

Figure 2 depicts the synthesis of pyrido[1,2,4,5]tetrazines via two pathways: [3 + 3]-and [5 + 1]-annulations. The first pathway was developed under solvent-free conditions via [3 + 3] cyclocondensation of **1**^19^ and the binucleophilic thiosemicarbazide. The reaction gave the corresponding 3-aminopyrido[1,2-*b*][1,2,4,5]tetrazine **7** in 89% yields. The reaction mechanism of compound **7** was hypothesized to proceed via the elimination of both H_2_O and H_2_S molecules as follows: initial condensation of the carbonyl functionality of compound **1** with the primary amine group of the thiosemicarbazide to give an intermediate by removal of a water molecule, followed by ring closure via removal of a hydrogen sulphide molecule to give the target compound **7**, which can be tautomerized to its tautomeric form, as illustrated in Fig. 2. The ^1^H-NMR spectrum of compound **7** recorded two singlet signals at 6.43 and 12.60 ppm exchangeable with D_2_O, attributed to amine and tetrazine-NH, respectively. It is important to note that the fusion of substrate **1**^19^ with a different reactive reagent, hydrazinecarbonitrile, produced the tautomer of compound **7** through a different mechanism and resulted in 3-imino-2*H*-pyrido[1,2-b][1,2,4,5]tetrazine **6** in a 67% yield (Fig. 2). A reaction mechanism is postulated with an initial nucleophilic attack on the carbonyl functional group of compound **1** by the amino nitrogen of the hydrazinecarbonitrile, providing an intermediate by the elimination of a water molecule. The intermediate then undergoes cyclization by attacking the primary amino group on the nitrile carbon, leading to the formation of compound **6**.

The presence of a primary amine functionality in the ortho position to tetrazine-NH opens the way for subsequent chemical modification of tetrazines. Thus, tetrazine derivative **7** was a good substrate for synthesizing a wide range of five- and six-membered heterocycles with high yields via [3 + 2]-and [3 + 3]-cyclocondensation reactions with 1,2- and 1,3-synthons, respectively. The fused heterocyclic system (6:6:6) with two nitrogen ring connections was obtained by fusing compound **7** with a 1,3-dicarbonyl compound, such as diethyl malonate, resulting in the respective dihydropyrimidinone derivative **8**. The formation of the pyrimidine ring was confirmed through ^13^C-NMR with signals at δ 43.47, 160.74, and 163.55 ppm assignable for CH_2_-pyrimidine and two amidic carbonyl carbons, respectively (Fig. 2). While tricyclic imidazo[1,2-*b*]pyrido[1,2-*e*][1,2,4,5]tetrazine analogues **9** and **10** were formed through the treatment of compound **7** with active methylene compound as phenacyl bromide and 1,2-di electrophilic diethyl oxalate; respectively. Compounds **9** and **10**’s structures were determined using spectroscopic data (see the Supplementary file).

**Figure 3:**
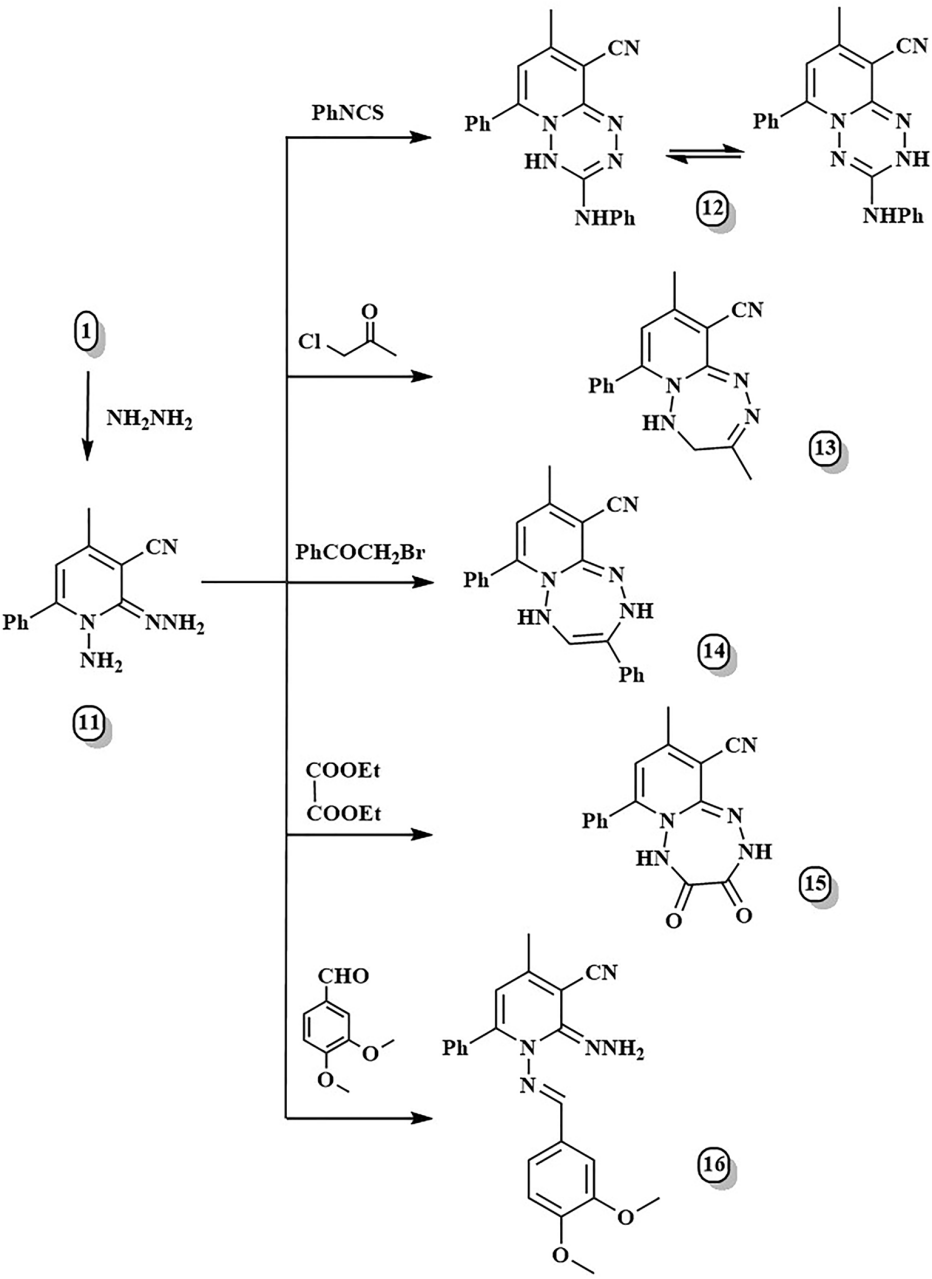
Synthesis of compounds **11**–**16**.

Another important starting point was hydrazone derivative **11**, formed by hydrazinolysis of the substrate **1**^19^ with an equimolar amount of hydrazine hydrate in the absence of solvent (Fig. 3). The latter compound was affirmed by the appearance of a new deuterium oxide exchangeable singlet in ^1^H NMR at δ 5.49 ppm due to =N–NH_2_ protons. In addition, the ^13^C NMR spectrum assured the absence of a carbonyl signal as well as the existence of a signal at δ 146.49 ppm correlated to C = N. Compound **11** was utilized as another way for the preparation of [1, 2, 4, 5]tetrazine and derivatives of [1, 2, 4, 5]tetrazepine via [5 + 1]- and [5 + 2]-annulation, respectively. The fusion of hydrazone derivative **11** with one carbon compound as phenylisothiocyanate was the second pathway for synthesizing pyrido[1,2,4,5]tetrazine **12** via [5 + 1]-annulation. The mechanism of compound **12** was expected to take place first via Michael's addition of the primary amine of **11** to the electron-deficient double bond in PhNCS, generating a Michael-type open-chain adduct (thiocarbamoyl intermediate) that underwent a cyclocondensation reaction with H_2_S removal. Compound **12** displayed characteristic ^1^H-NMR signals at δ 7.86 ppm and 4.03 ppm corresponding to NH-phenyl and NH-tetrazine, respectively, whereas the ^13^C NMR demonstrated signals at 152.56 and 154.74 ppm due to C=N-tetrazine carbons. On the other hand, the catalyst- and solvent-free [5 + 2]-cyclocondensation was applied to synthesize pyrido[1,2-*b*][1,2,4,5]tetrazepine derivatives **13** and **14** by fusing the key starting material **11** with α-halo carbonyl compounds, namely chloroacetone and phenacyl bromide, respectively at 140–160 °C. Additionally, 2,3,4,5-tetrahydropyrido[1,2-*b*][1,2,4,5]tetrazepine-3,4-dione **15** was easily obtained in high yield by treating hydrazone derivative **11** with diethyl oxalate. ^1^H-NMR demonstrated new signals at 8.44 and 8.56 ppm for two tetrazepine-NH protons, respectively, whereas ^13^C-NMRR displayed distinguishable signals at 151.08 and 152.51 ppm for two C=O carbons. Finally, Schiff base **16** can be formed through the condensation of compound **11** with 3,4-dimethoxybenzaldehyde. The ^1^H-NMR spectrum of **16** showed a singlet signal at 7.69 ppm due to the (CH=N–) proton.

### Biological activity

#### NCI screening of anticancer activity

##### Preliminary single high dose screening at 10^–5^ M concentration

Twelve synthesized derivatives were tested for in vitro anticancer activity; some showed modest activity in numerous cancer cell lines, while others showed impact against further than one cancer cell line. Table 1 shows the most sensitive cell lines' growth inhibition percentages (GI %).

**Figure 4:**
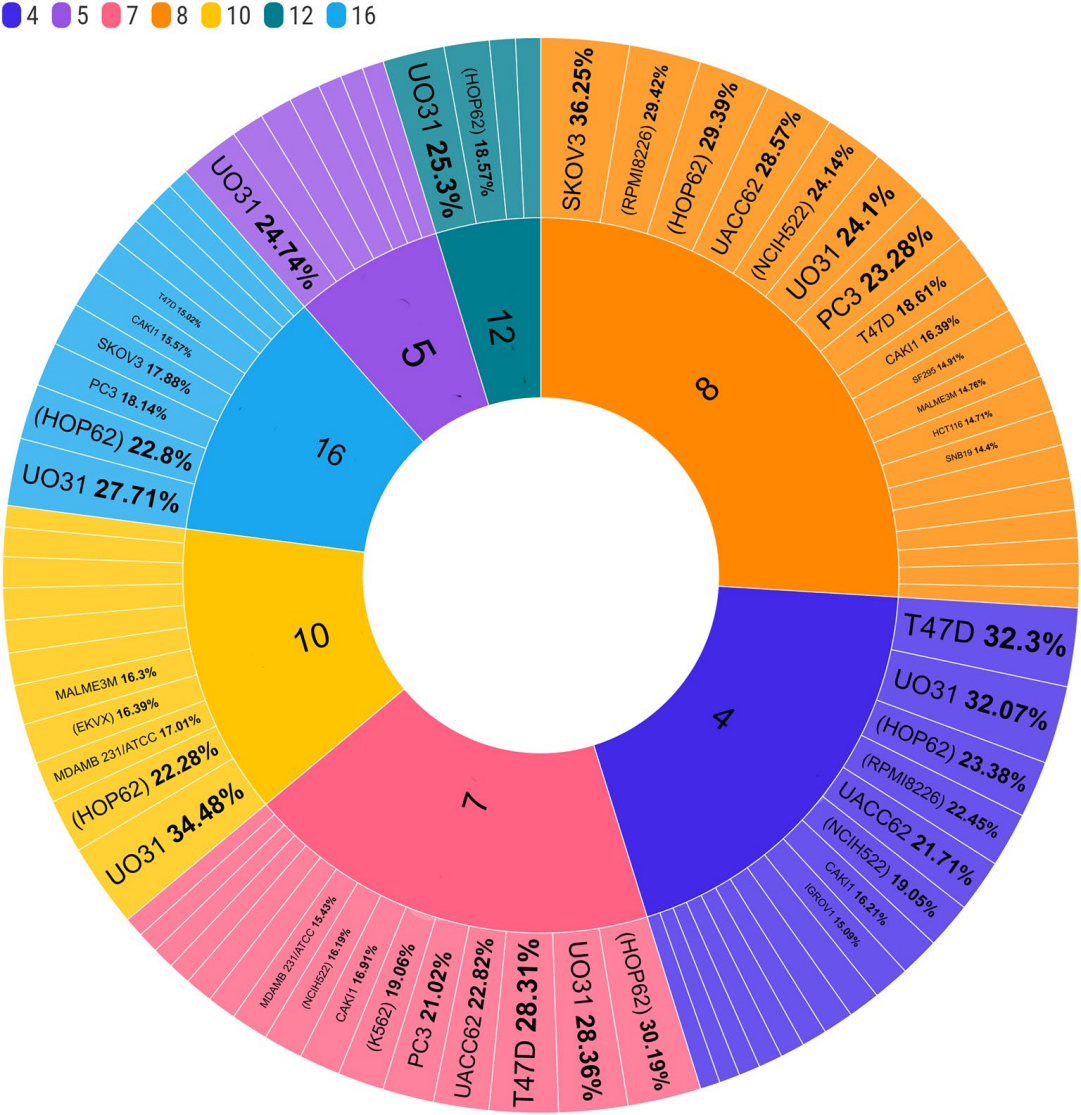
Sunburst chart represents **GI% **higher than **15%** for compounds **4**, **5**, **7**, **8**, **10**, **12**, and **16** against cancer cell lines of the nine tumour panels.

More specifically, compounds (**4**, **5**, **7**, **8**, **10**, **12**, and **16**) showed moderate activity with a mean GI% range (of 21.02–36.25) % against several forms of cancers, including Leukemia (RPMI-8226), non-small cell lung cancer (HOP-62 & NCI-H522), Melanoma (UACC-62), Renal cancer (UO-31), Brest cancer (T-47D), Prostate cancer (PC-3), and Ovarian Cancer (SK-OV-3) (Fig. 4).

**Figure 5:**
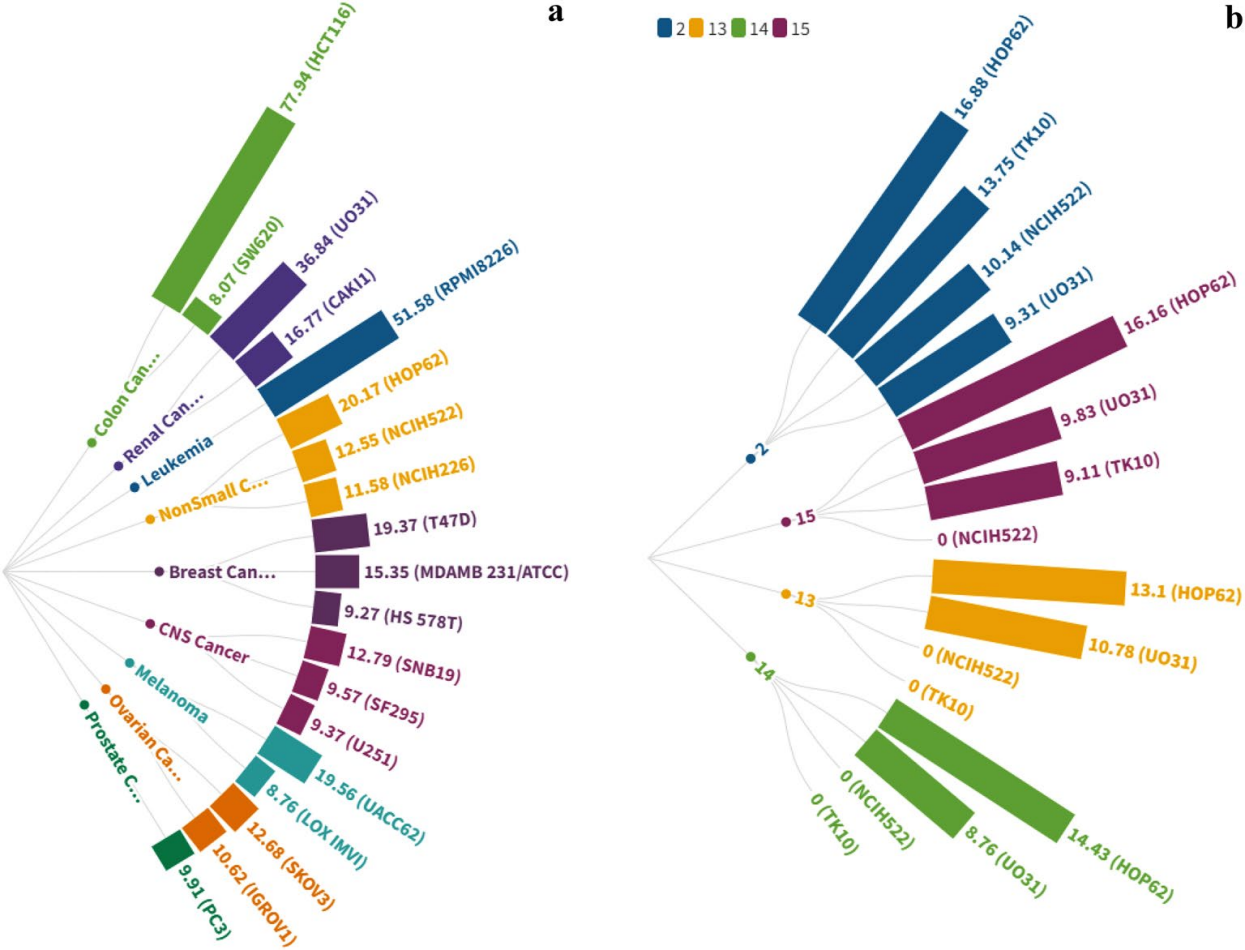
Radial bar chart represents (**a**): **GI%** of compound **9** against cancer cell lines of the nine tumour panels. (**b**): **GI%** of compounds (**2**, **13**, **14**, and **15**) against cancer cell lines of the nine tumour panels.

Among all tested compounds, **9** demonstrated strong anticancer cell line efficacy against Leukemia-RPMI-8226 (GI% = 51.58%) and Colon cancer-HCT116 (GI% = 77.94%). On the other hand, compound **9** inhibited various cell lines in a mild to moderate manner with a mean GI% range (of 8.07–36.84) % (Fig. 5a).

Compounds **2** and **13**–**15** were ineffective against the bulk of the subpanels of cancer cell lines. They revealed a weak effective anticancer efficacy against certain cell lines, such as Non-small cell lung cancer (HOP-62 & NCI-H522) and Renal cancer (UO-31 & TK-10) with GI% range (8.76–16.88)% (Fig. 5b). The screening findings revealed that **4, 8,** and** 9** exhibited the maximum activity in some of the cell lines in the current study.

#### Analysis of molecular docking

**Figure 6:**
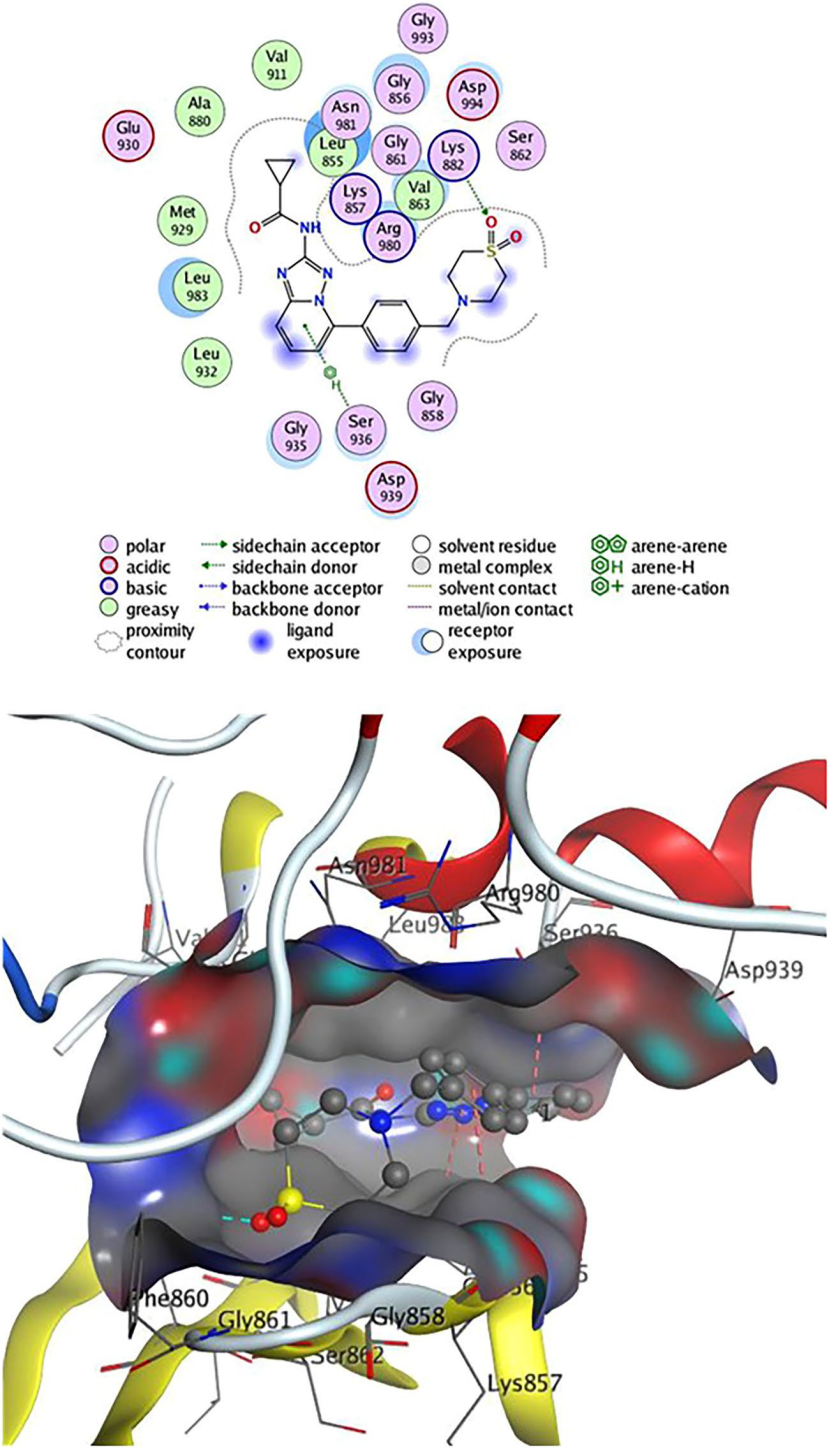
2D& 3D interaction of **2HB** in the active site of Janus kinase **Jak2** (PDB ID: **4P7E**). Hydrogen bonds are displayed in cyan & H-pi-bonds are in dark pink.

The conjugates (**4**, **8**, and **9**) exceeded all other produced fused pyridine derivatives in terms of biological activity against various cancer cell lines. To examine their binding mechanism and non-bonding effects, we did a molecular docking versus Janus Kinase (Jak2) (PDB: 4P7E). The docking process was first confirmed by re-docking the co-crystallized ligand **2HB** (N-(5–4)[(1,1-dioxidothiomorpholin-4-yl)methyl]phenyl[1,2,4]triazolo[1,5-*a*]pyridin-2-yl)cyclopropane carboxamide) at the enzyme's active sites. **2HB** has an energy score (S) =  − 7.45 kcal/mol and showed one H-bond as Lys 882 with SO_2_ group and three arene-H interactions with Gly 856, Ser 936, and Leu 855 residues (Fig. 6).

**Figure 7:**
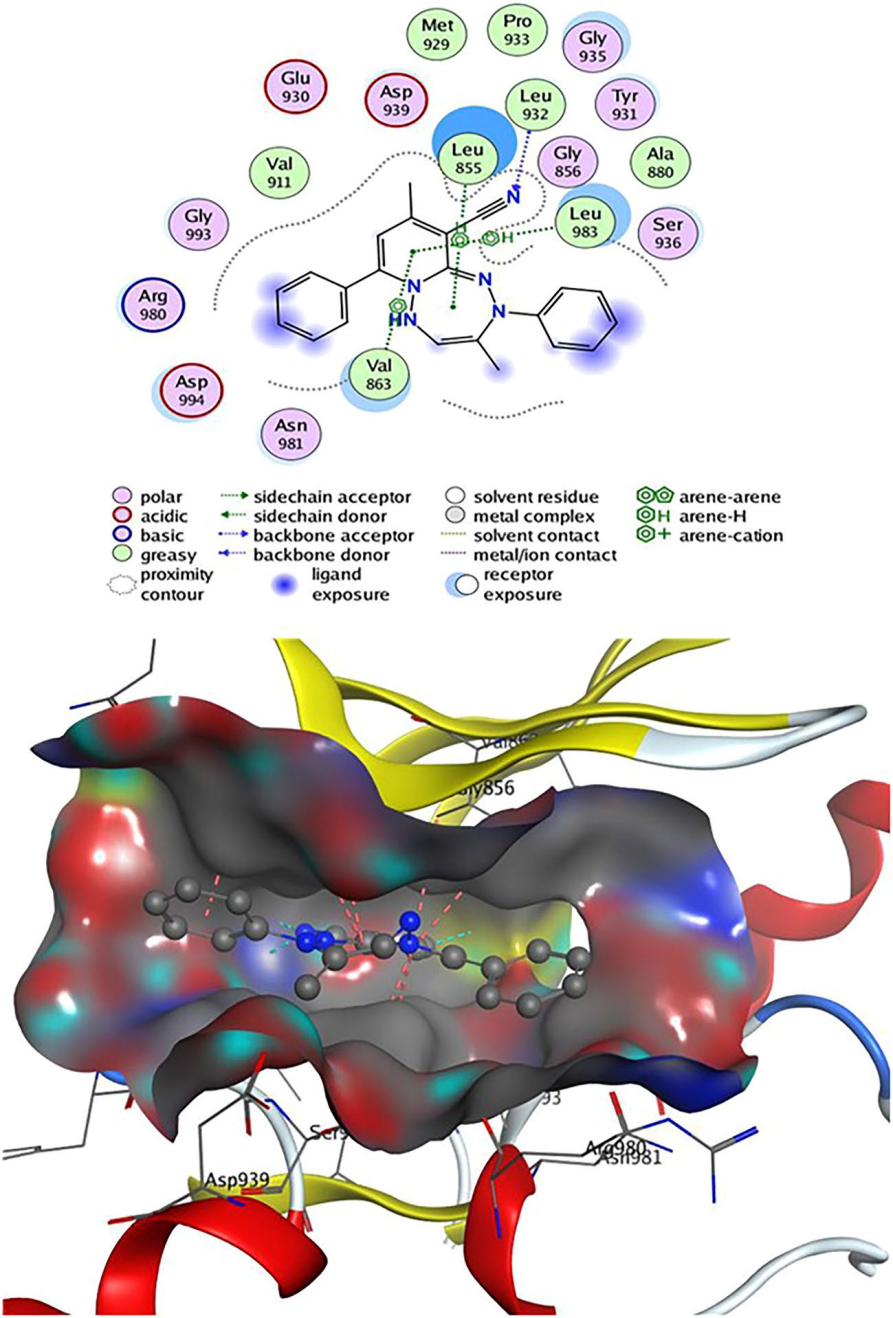
2D& 3D interaction of **4** in the active site of Janus kinase **Jak2** (PDB ID: **4P7E**). Hydrogen bonds are displayed in cyan & H-pi-bonds are in dark pink.

**Figure 8:**
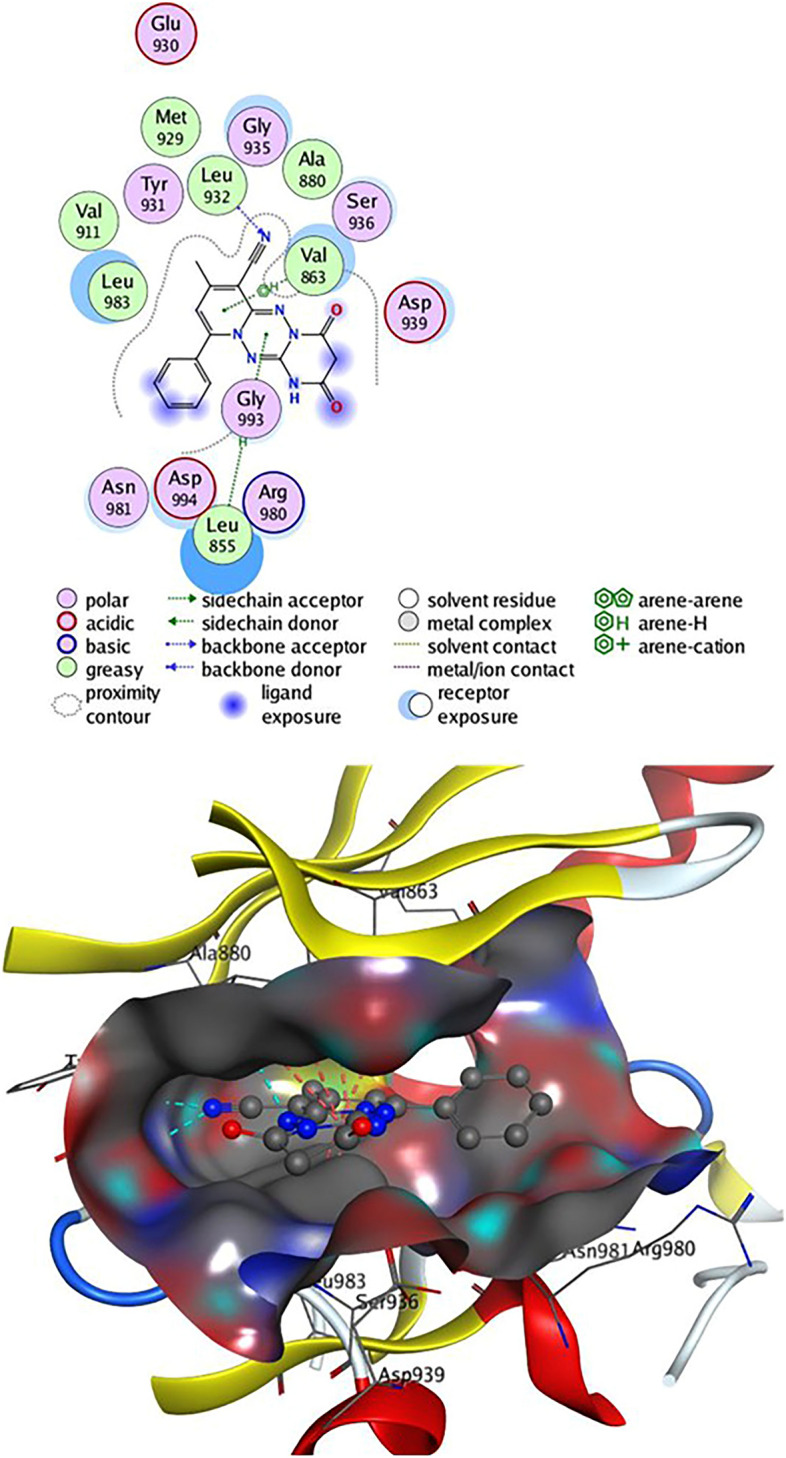
2D& 3D interaction of **8** in the active site of Janus kinase **Jak2** (PDB ID: **4P7E**). Hydrogen bonds are displayed in cyan & H-pi-bonds are in dark pink.

**Figure 9:**
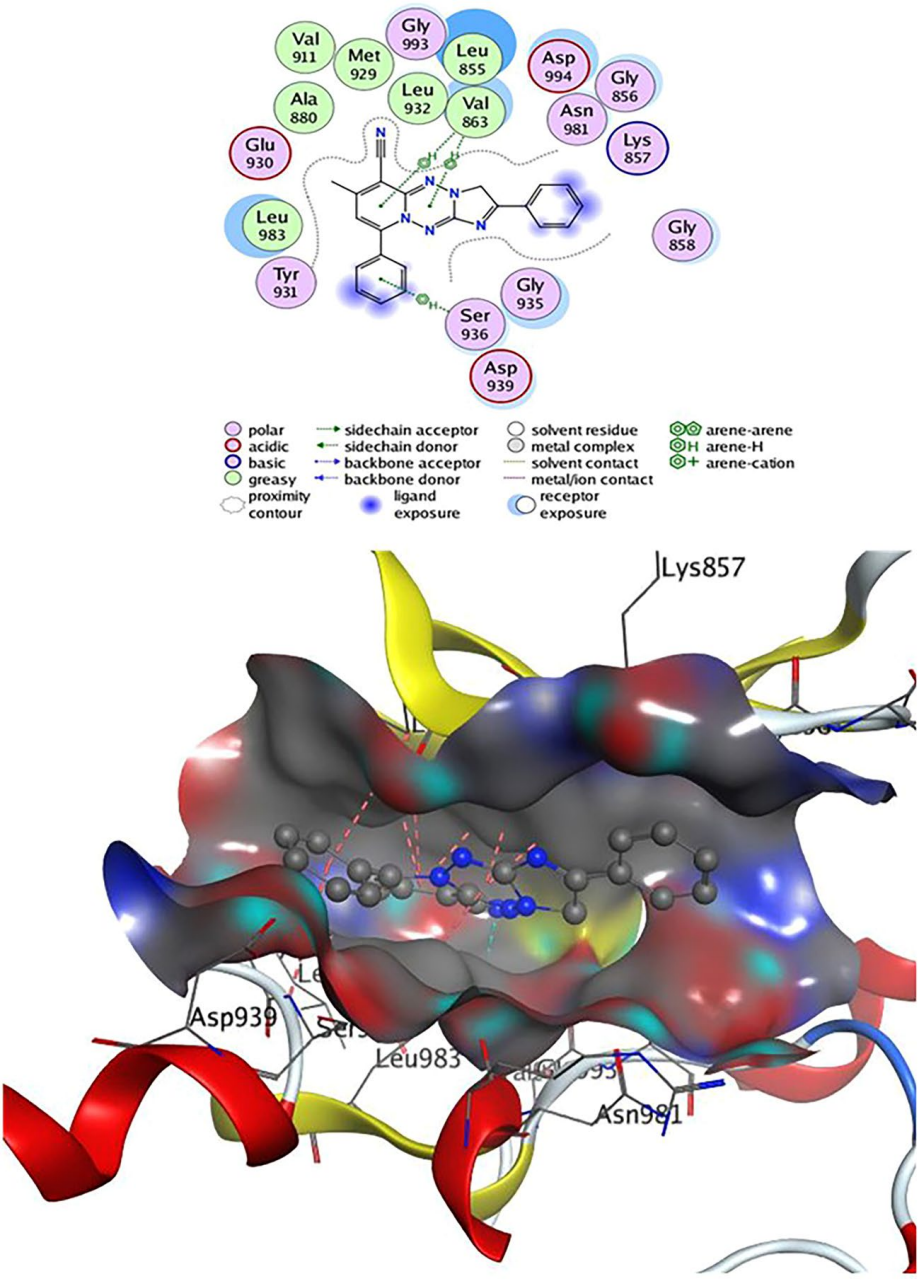
2D& 3D interaction of **9** in the active site of Janus kinase **Jak2** (PDB ID: **4P7E**). Hydrogen bonds are displayed in cyan & H-pi-bonds are in dark pink.

Table 2 revealed that compounds **4** and **9** have an energy score (S) =  − 7.24 and − 7.25 kcal/mol, respectively, close to that of the **2HB** ligand. Compound **4** can establish two H-bonds as Leu 932 with CN group and one hydrogen bond between the CH_3_ group and the residue Met 929. On the other hand, Leu 855 showed three H-arene contacts with the benzene and tetrazepine ring of compound **4**, also the pyridine nucleus produced four arene-H interactions with Val 863 and Leu 983 amino acids (Fig. 7). Compound **8** had an energy score (S) =  − 6.56 kcal/mol, two H-bonds were observed between CN group and the residue Leu 932 and one H-bond formed between Leu 855 and N of tetrazine ring, also, Leu 855 showed two H-arene interaction with tetrazine ring. In addition, the pyridine ring of **8** was oriented in close contact with Leu 983 and Val 863, forming four arene-H interactions (Fig. 8). Finally, compound **9** established one hydrogen bond as Val 911 with CN group and eight arene-H contacts as benzene ring with Leu 855, pyridine ring with (Ser 936, Leu 855 & Val 863) and tetrazine ring with (Val 863 & Leu 983) (Fig. 9).

**Table 2:** Docking results of compounds **4**, **8**, **9,** and **2HB** ligand inside Janus Kinase **Jak2** (PDB ID: **4P7E**) active spots.

Janus Kinase (4P7E)						
Comp	Score	Affinity bond strength (Kcal/mol)	Affinity bond length (in A^o^ from the main residue)	Amino acids	Ligand functional group	Interaction
**4**	− 7.24	− 0.3	2.78	Leu 932	C≡N	H-acceptor
		− 3.6	3.23	Leu 932	C≡N	H-acceptor
		− 0.2	3.91	Met 929	CH_3_	H-donor
		− 0.4	3.56	Leu 855	Benzene ring	pi-H
		− 0.2	4.55	Leu 855	Tetrazepine ring	pi-H
		− 1.3	3.86	Leu 855	Tetrazepine ring	pi-H
		− 0.8	4.00	Val 863	Pyridine ring	pi-H
		− 0.5	4.54	Val 863	Pyridine ring	pi-H
		− 0.7	3.68	Leu 983	Pyridine ring	pi-H
		− 0.2	4.19	Leu 983	Pyridine ring	pi-H
**8**	− 6.56	− 0.3	2.79	Leu 932	C≡N	H-acceptor
		− 3.3	2.26	Leu 932	C≡N	H-acceptor
		− 0.2	3.92	Leu 855	N of tetrazine ring	H-acceptor
		− 0.3	4.66	Leu 855	Tetrazine ring	pi-H
		− 1.5	4.14	Leu 855	Tetrazine ring	pi-H
		− 0.2	4.12	Leu 983	Pyridine ring	pi-H
		− 0.5	3.56	Leu 983	Pyridine ring	pi-H
		− 0.4	4.63	Val 863	Pyridine ring	pi-H
		− 0.8	4.14	Val 863	Pyridine ring	pi-H
**9**	− 7.25	− 0.2	3.93	Val 911	C≡N	H-acceptor
		− 0.5	4.75	Leu 855	Benzene ring	pi-H
		− 0.8	3.79	Ser 936	Pyridine ring	pi-H
		− 0.3	4.09	Leu 855	Pyridine ring	pi-H
		− 0.3	4.59	Leu 855	Pyridine ring	pi-H
		− 0.6	4.45	Val 863	Pyridine ring	pi-H
		− 0.5	4.12	Val 863	Tetrazine ring	pi-H
		− 1.0	4.09	Val 863	Tetrazine ring	pi-H
		− 0.2	4.26	Leu 983	Tetrazine ring	pi-H
**2HB**	− 7.45	− 1.1	3.31	Lys 882	SO_2_	H-acceptor
		− 0.3	4.65	Gly 856	Benzene ring	pi-H
		− 0.6	4.24	Ser 936	Pyridine ring	pi-H
		− 0.3	4.59	Leu 855	Triazole ring	pi-H

#### Analysis of frontier molecular orbitals

**Figure 10:**
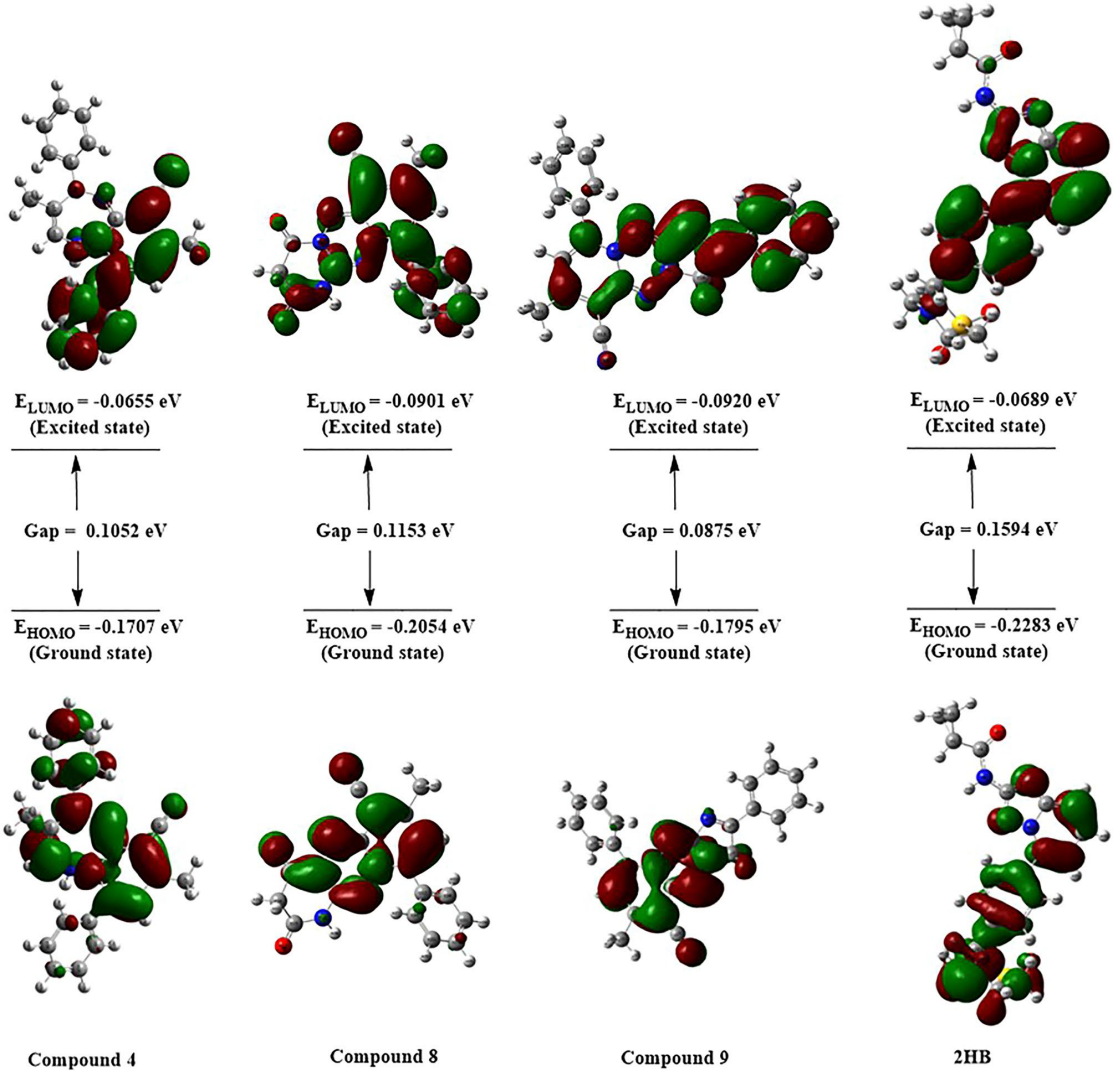
Molecular orbital distribution plots of HOMO and LUMO for compounds **4**,** 8**, **9,** and** 2HB.**

The most fundamental orbitals in molecules are the frontier molecular orbitals, which are used to determine kinetic stability and chemical reactivity. The highest occupied molecular orbital (HOMO) and the lowest unoccupied molecular orbital (LUMO) are the names given to the Frontier molecular orbitals. Figure 10 shows that the HOMO orbitals are less localized than the LUMO orbitals. Electronic absorption is the transition from the ground state to the first excited state and is best defined by the excitation of an electron from HOMO to LUMO^26^. Kinetic stability rises when the HOMO–LUMO gap widens. As a result, moving electrons from the stable state HOMO to the excited state LUMO needs more energy. For compounds **4**, **8**, and **9**, the majority of the HOMO is placed mainly on the pyridine ring and the fused triazepine or triazine rings, with a little contribution from the phenyl ring connected to triazepine nitrogen atom as in compound **4**. Compounds **4** and **8** have LUMOs localized on the pyridine ring, nitrogen atoms of the fused ring, and the phenyl ring attached to pyridine. In contrast, compound **9** has LUMOs localized on the pyridine ring, 4-phenyl imidazole moiety, and nitrogen atoms of fused triazine. Table 3 displays orbital energy and dipole moment values calculated for the selected compounds (**4**, **8, 9**) and **2HB**. The dipole moments of the more potent conjugates and **2HB** were investigated, revealing that the compounds have large dipole moments in the order **8** > **2HB** < **4** > **9**. Although, compounds **4**, **8,** and **9** have an energy gap of 0.1052, 0.1153, and 0.0875 eV, respectively, which are smaller than that of **2HB** (0.1594 eV) (Fig. 10). As a result, these chemicals (**4**,** 8** and** 9**) may enable higher interaction with high dipole moment species, particularly in biological systems.

**Table 3:** DFT parameters calculated for the synthesized compounds (**4, 8, 9**) & **2HB**.

Comp No.	Dipole moment, μ (Debye)	E_HOMO_ (eV)	E_LUMO_ (eV)	(HOMO–LUMO) gaps (eV)
**4**	8.2089	− 0.1707	− 0.0655	0.1052
**8**	9.7371	− 0.2054	− 0.0901	0.1153
**9**	5.0329	− 0.1795	− 0.0920	0.0875
**2HB**	7.2947	− 0.2283	− 0.0689	0.1594

*DFT* density functional theory, *HOMO* highest occupied molecular orbital, *LUMO* lowest unoccupied molecular orbital.

#### The electrostatic potential of molecules (MEP)

**Figure 11:**
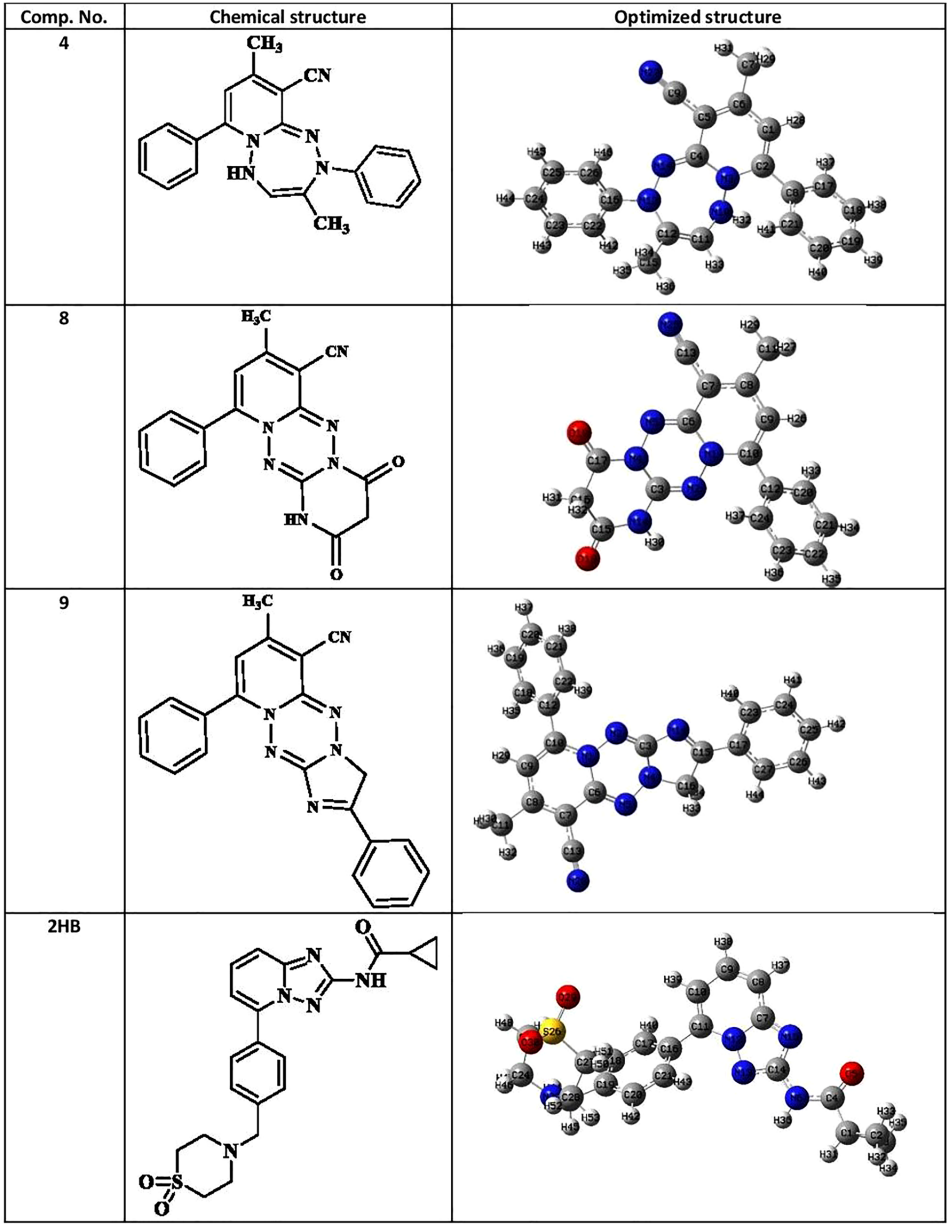
Chemical and optimized structure of compounds (**4**, **8**, **9**) and **2HB**. Optimized with DFT-B3LYP/6-31G.

**Figure 12:**
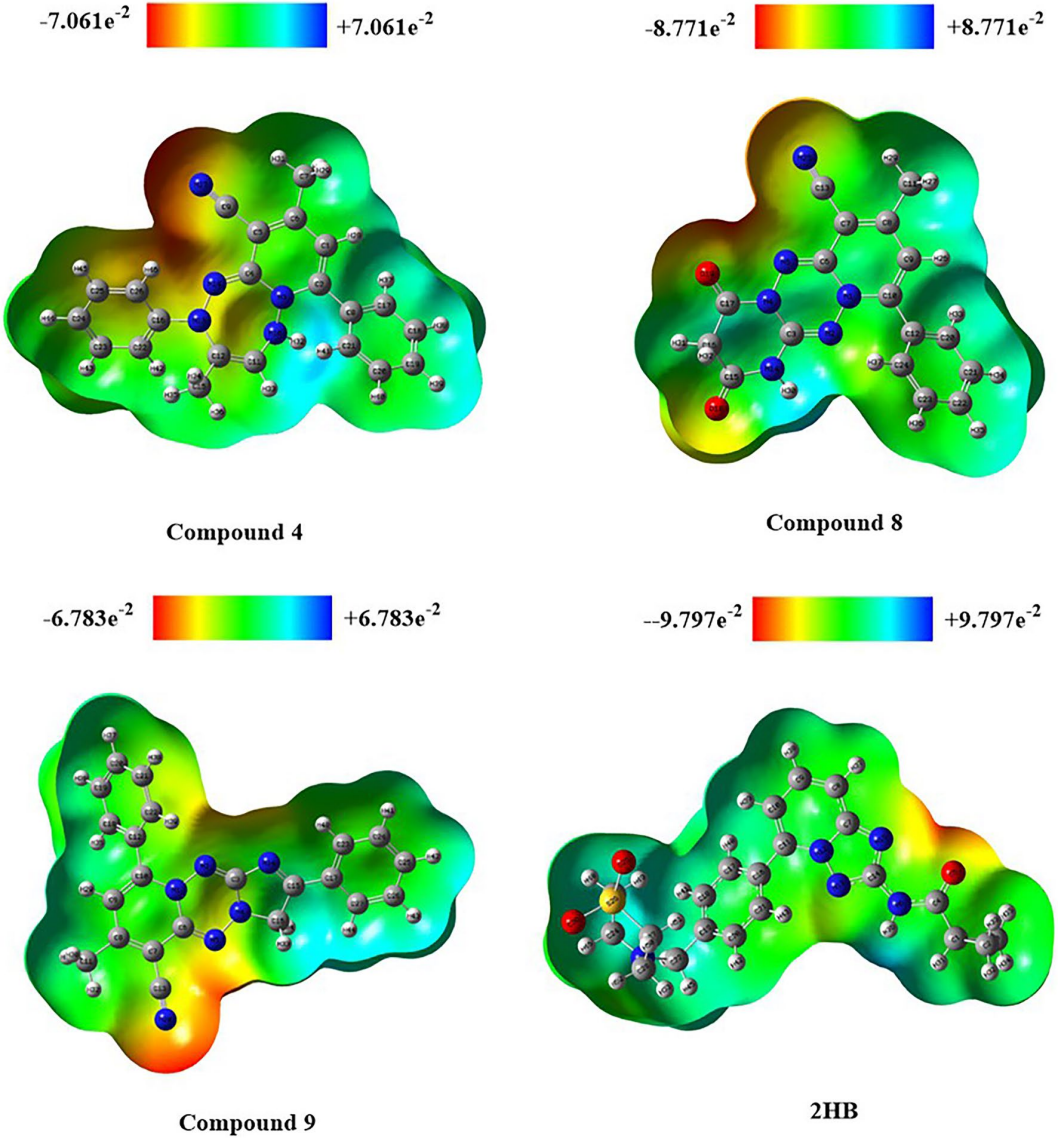
MEP map of compounds (**4**, **8**, **9**) and **2HB**.

The molecular electrostatic potential (MEP) aids in interpreting the biological recognition phase and hydrogen bonding connections^27^. The MEP map of compounds (**4**, **8**, **9**) and **2HB** was generated using the B3LYP with the basis set 6-31G optimized outputs (Fig. 11). MEP was computed to identify the reactive functional groups for electrophilic and nucleophilic reactions in terms of colour grading of the optimized structure of compounds (**4**, **8**, **9**) and **2HB** (Fig. 12). The red colour represents the maximum negative area, which suggests a desirable site for electrophilic attacks, the blue colour represents the largest positive area, which indicates a favourable site for nucleophilic attacks, and the green colour represents zero potential regions.

#### In silico ADME studies

##### Physicochemical properties and drug-likeness

A bioinformatics study was performed on the most active compounds **4, 8**, and **9** to predict their physicochemical and drug-likeness properties. The physicochemical properties for oral bioavailability, which include (size: MW between 150 and 500 g/mol, lipophilicity: Log P between − 0.7 and + 5.0, polarity: TPSA between 20 and 140 Å, flexibility: no more than 10 rotatable bonds, and solubility: log S not higher than 6) demonstrated that all examined compounds have good physicochemical properties similar to the reference 2HB (Table 4). Furthermore, compounds should meet the following criteria under the Lipinski rule of five, which indicates that drugs have good absorption and bioavailability: M.wt. ≤ 500, log P ≤ 5, HBD ≤ 5, and HBA ≤ 10, as shown in Table 4. The examined compounds **4, 8,** and **9** had good values for all of the rule principles with potential drug-like properties, indicating that these compounds may meet cell membrane permeability and bioavailability requirements.

**Table 4:** In silico physicochemical properties and drug-likeness of compounds **4, 8,** and **9,** as well as the reference **2HB.**

Molecule	^a^MW ≤ 500	^b^Log *P*_o/w_ ≤ 5	^c^TPSA Ǻ^2^ ≤ 140	^d^NRB ≤ 10	^e^Log S	^f^HBA ≤ 10	^g^HBD ≤ 5	Lipinski violations
(**4**)	353.42	4.73	61.81	2	− 5.9**	2	1	0
(**8**)	332.32	1.35	105.08	1	− 3.45*	5	1	0
(**9**)	364.4	3.69	71.27	2	− 5.17**	4	0	0
(**2HB**)	425.5	2.27	105.05	6	− 3.31*	6	1	0

^a^MW, molecular weight.

^b^Log *P*_o/w_, partition coefficient octanol/water.

^c^TPSA, topological polar surface area.

^d^NRB, number of rotatable bonds.

^e^Log S, Aqueous solubility (*soluble, **moderately soluble).

^f^HBA, number of H-bond acceptors.

^g^HBD, number of H-bond donors.

##### The ADME profile

Further on, the SwissADME Web tool was used to investigate the pharmacokinetics of compounds **4, 8,** and **9,** as well as the reference 2HB. According to the Boiled-egg model^28^, all compounds had high gastrointestinal absorption (GI), indicating their ability to easily absorb through the intestinal wall. Compounds **4** and **9** were also shown to cross the blood–brain barrier (BBB), which may be useful in anticancer drugs targeting the CNS. In contrast, compound **8** was distinguished by a lack of BBB permeability, indicating the absence of CNS drawbacks like the reference 2HB (Table 5). Furthermore, compounds **4, 8,** and **9** were shown not to bind to P-glycoproteins, in contrast to the reference 2HB.

**Table 5:** In silico pharmacokinetic study of compounds **4, 8,** and** 9** as well as the reference **2HB.**

Cpd. No.	BBB permeant	GI absorption	Pgp substrate	Cytochrome P450 (CYP inhibitor)				
				CYP1A2 inhibitor	CYP2C19 inhibitor	CYP2C9 inhibitor	CYP2D6 inhibitor	CYP3A4 inhibitor
(**4**)	Yes	High	No	Yes	Yes	Yes	Yes	No
(**8**)	No	High	No	No	No	No	No	No
(**9**)	Yes	High	No	Yes	Yes	Yes	No	No
(**2HB**)	No	High	Yes	No	No	Yes	No	Yes

Moreover, the compounds' interactions with cytochromes P450 (CYP) in the liver, which is the main factor for drug elimination via metabolic biotransformation, have been investigated. Compounds **4** and **9** are indicated to inhibit four and three of the five major isoforms of hepatic cytochrome P-450 (CYP), respectively, and should be administered at time intervals when other drugs are prescribed to avoid potential drug interactions, similar to the reference 2HB, which is indicated to inhibit both CYP2C9 and CYP3A4. On the other hand, compound **8** is expected to inhibit none of the cytochrome P-450 (CYP) isoforms in the liver, making it safe to use together with other drugs (Table 5).

## Conclusion

The fusion technique was applied for synthesizing several novel bioactive nitrogen-rich heterocycles, including pyrido-annelated [1, 2, 4, 5]tetrazines, [1, 2, 4]triazepine, and [1, 2, 4, 5]tetrazepines, with yields ranging from 67 to 89%. Additionally, molecules **4**, **8**, and **9** had the highest anticancer effect against many cancer cell lines. Furthermore, compounds **4**, **8**, and **9** have low HOMO–LUMO energy gaps, so that they may become more reactive than the **2HB** ligand. Moreover, the docked complex of analogues **4**, **8**, and **9** with **4P7E** had a higher binding affinity. The MEP study findings revealed the most negative and positive surface areas of the examined conjugates, allowing us to predict potential hydrogen bonding sites. Finally, In silico investigations of the compounds produced encouraging results, including strong GI absorption, good oral bioavailability, and perfect physicochemical features, indicating their potential as attractive medicinal targets “Supplementary Information”.

## Supplementary Information

Supplementary Information.

## Data Availability

The datasets generated and analyzed during the current study are available at https://www.scidb.cn/anonymous/aUFSYlF6.
