# Supplementary material for: Synthesis of pyrido-annelated [1,2,4,5]tetrazines, [1,2,4]triazepine, and [1,2,4,5]tetrazepines for anticancer, DFT, and molecular docking studies
Source: Sci Rep. 2023 Apr 5;13:5585. doi: 10.1038/s41598-023-32421-x (PMC10076274; doi:10.1038/s41598-023-32421-x)
Supplement: Supplementary file 1 — Supplementary Information. [file 41598_2023_32421_MOESM1_ESM.pdf]

Synthesis Of Pyrido-Annelated [1,2,4,5]Tetrazines, [1,2,4]Triazepine, And [1,2,4,5]Tetrazepines For Anticancer, DFT, And Molecular Docking Studies.

Aisha Y. Hassan[1] | Sara N. Shabaan[1] | Samiha A. El-Sebaey[2] | Eman S. Abou-Amra[1*]

[1] Department of Chemistry, Organic Chemistry, Faculty of Science (Girls), Al-Azhar University, Youssef Abbas Street, Nasr City, Cairo, Egypt.

[2] Department of Pharmaceutical Organic Chemistry, Faculty of Pharmacy (Girls), Al-Azhar University, Youssef Abbas Street, Nasr City, Cairo, Egypt.

***Correspondence: Eman S. Abou-Amra, email: emansadek.59@azhar.edu.eg ,**

eman.sadek.1612@gmail.com.

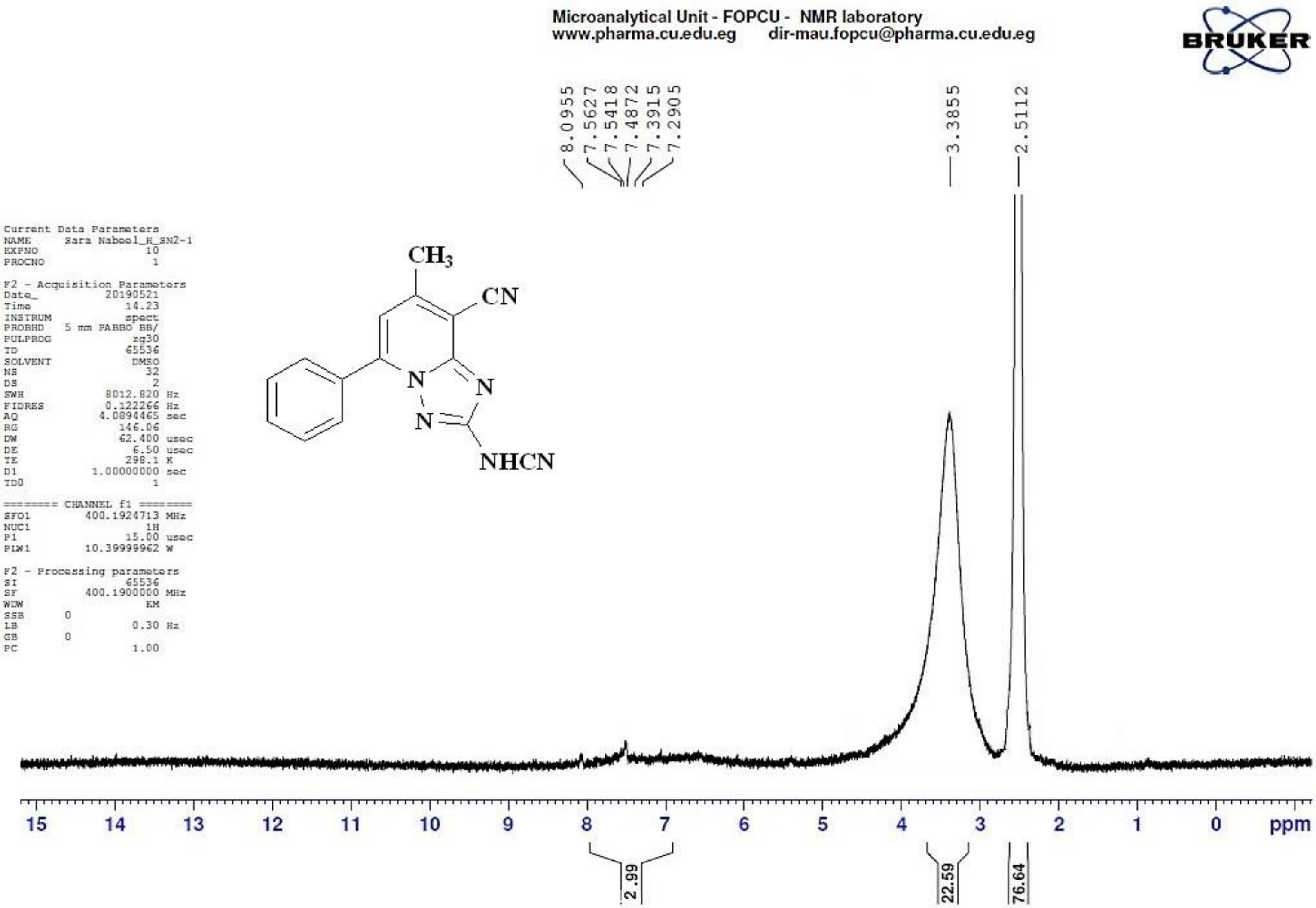

Supplementary fig. (1): ^{1}H NMR for compound **2**.

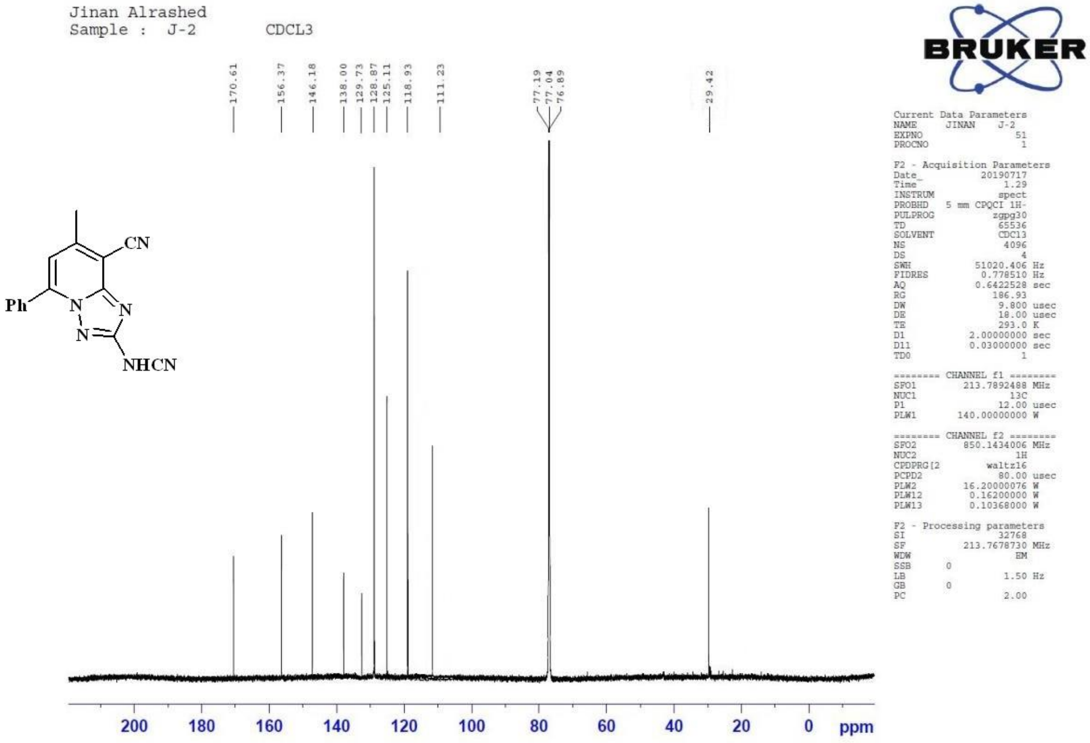

Supplementary fig. (2): ^{13}C NMR for compound **2**.

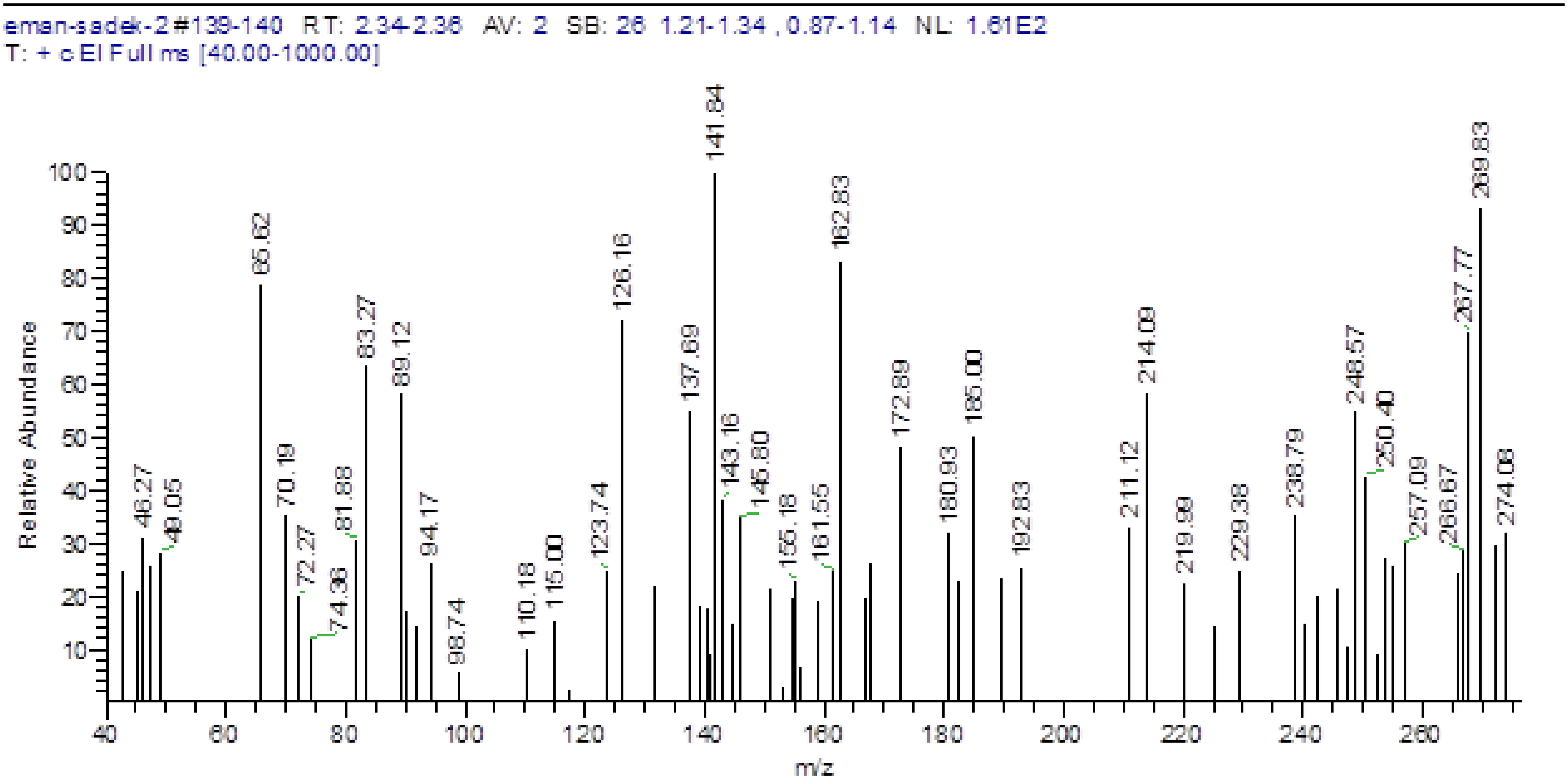

Supplementary fig. (3): mass spectrum for compound **2**.

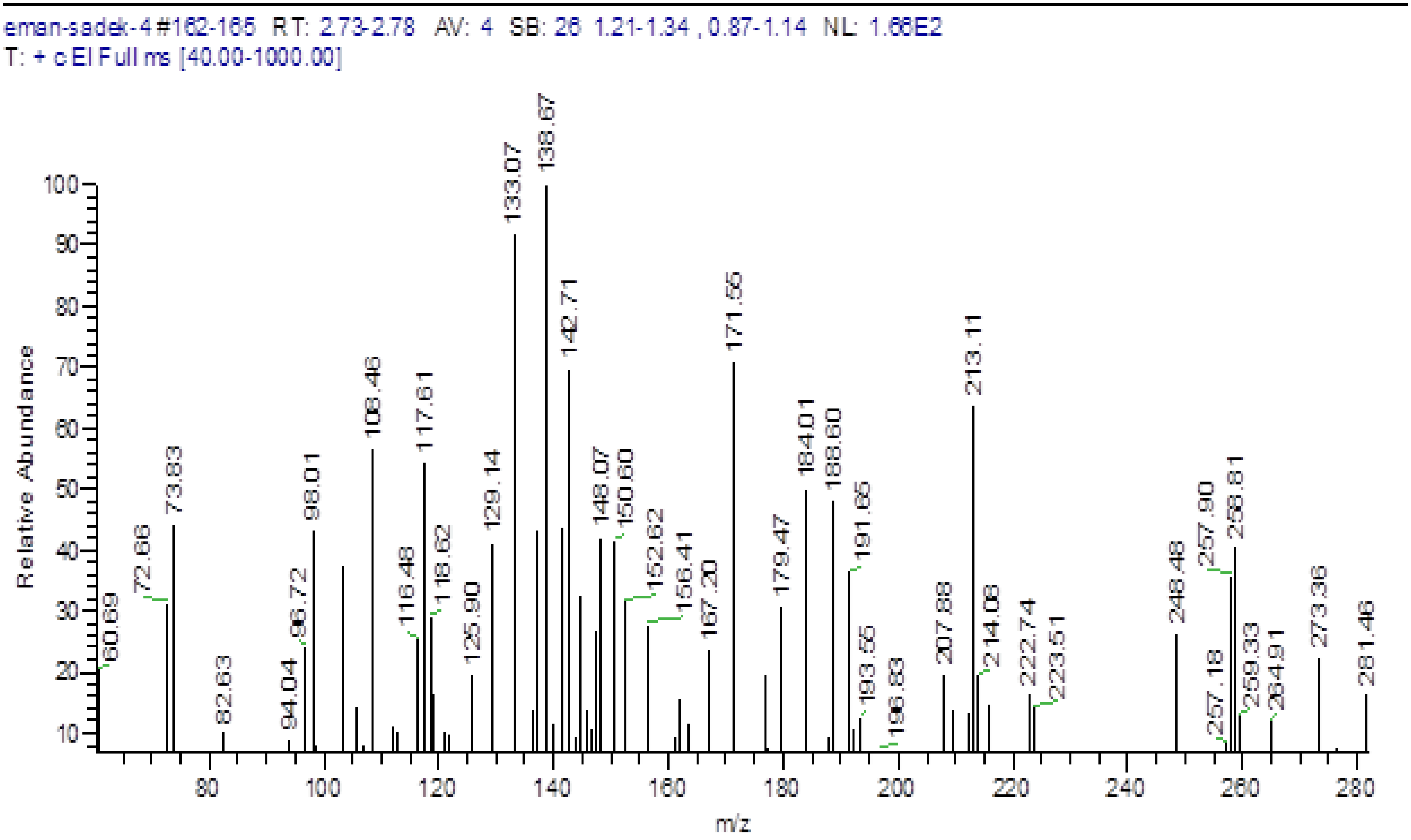

Supplementary fig. (4): mass spectrum for compound **3**.

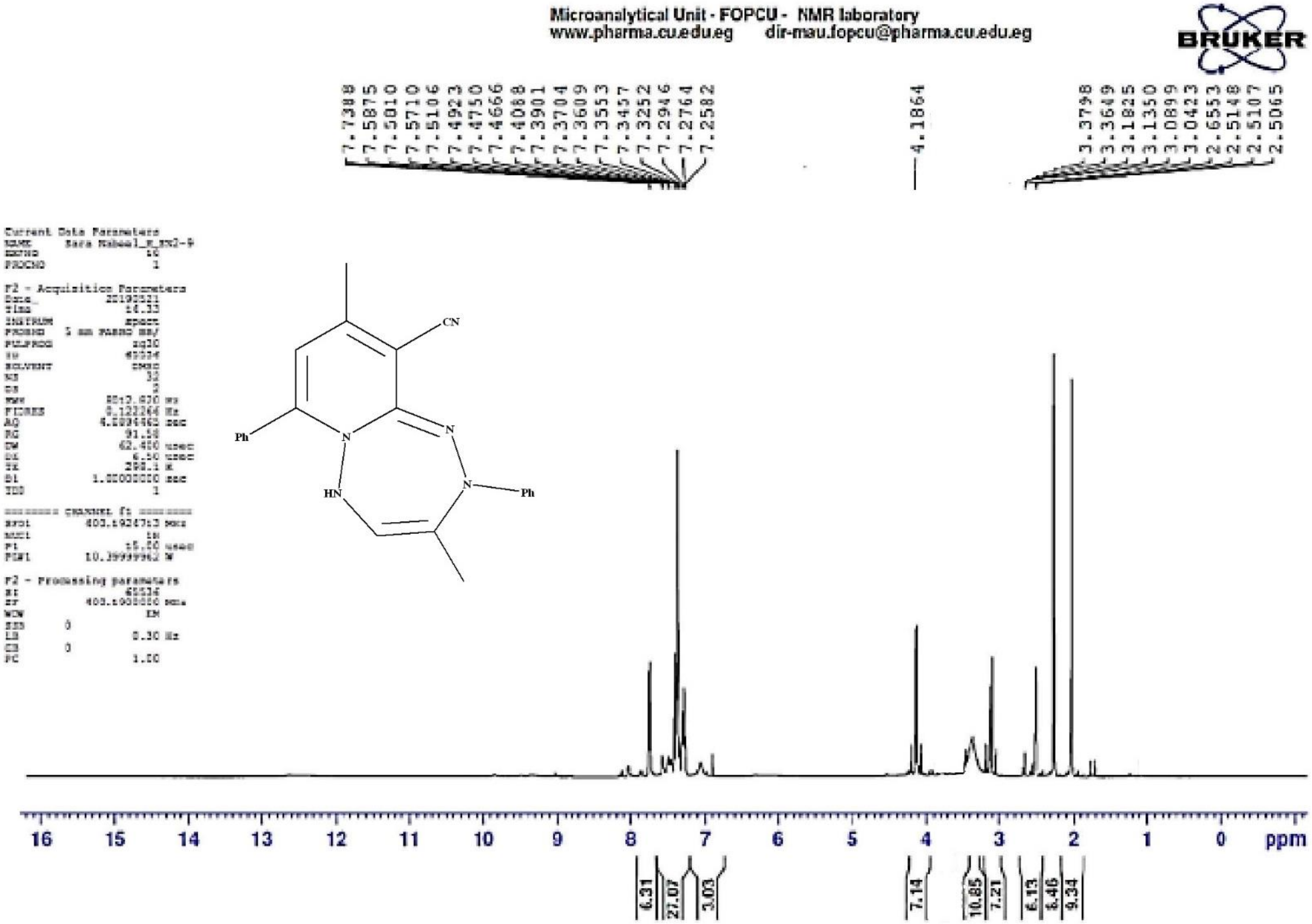

Supplementary fig. (5): ^{1}H NMR for compound **4**.

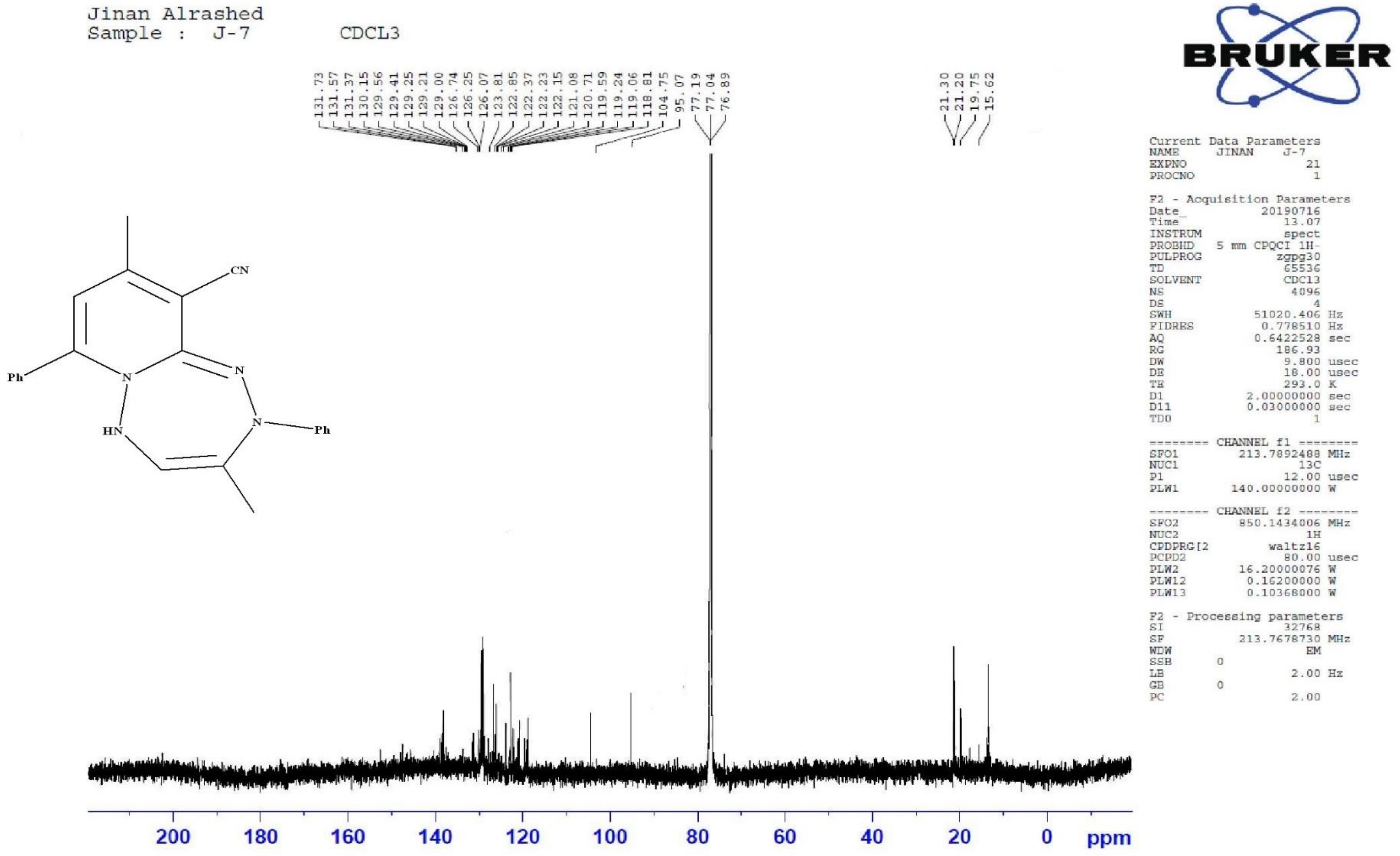

Supplementary fig. (6): ^{13}C NMR for compound **4**.

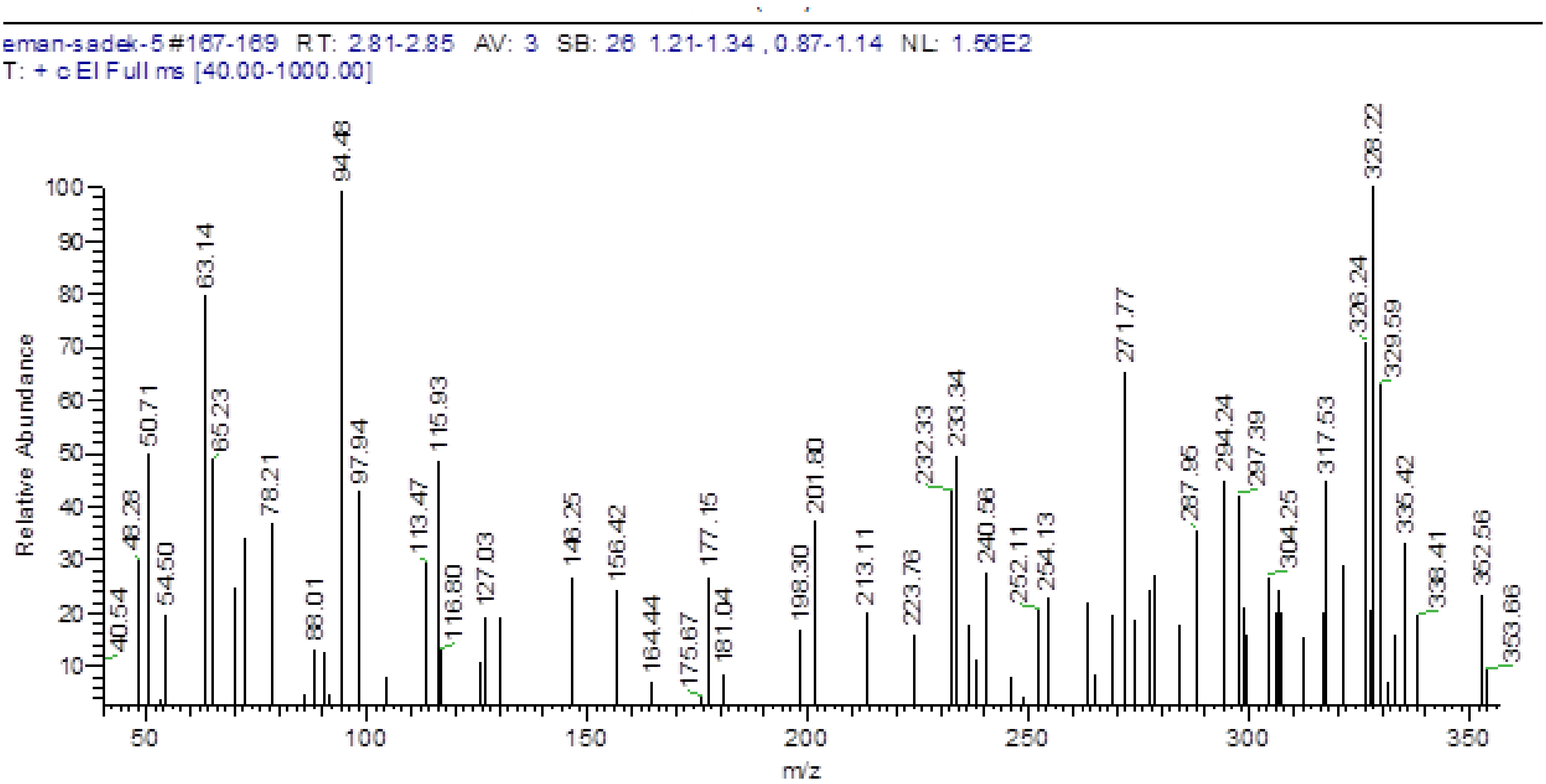

Supplementary fig. (7): mass spectrum for compound **4**.

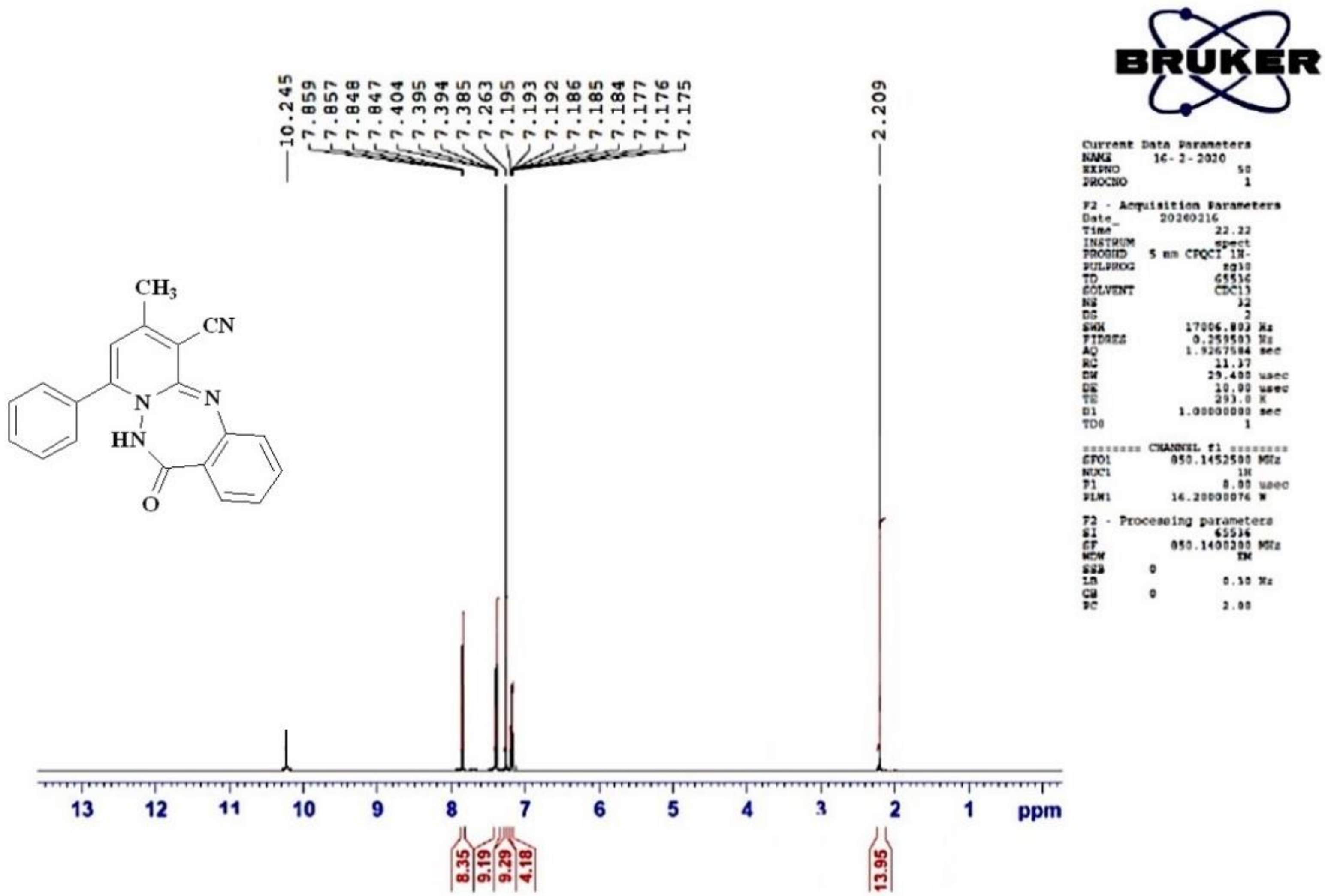

Supplementary fig. (8): ^{1}H NMR for compound **5**.

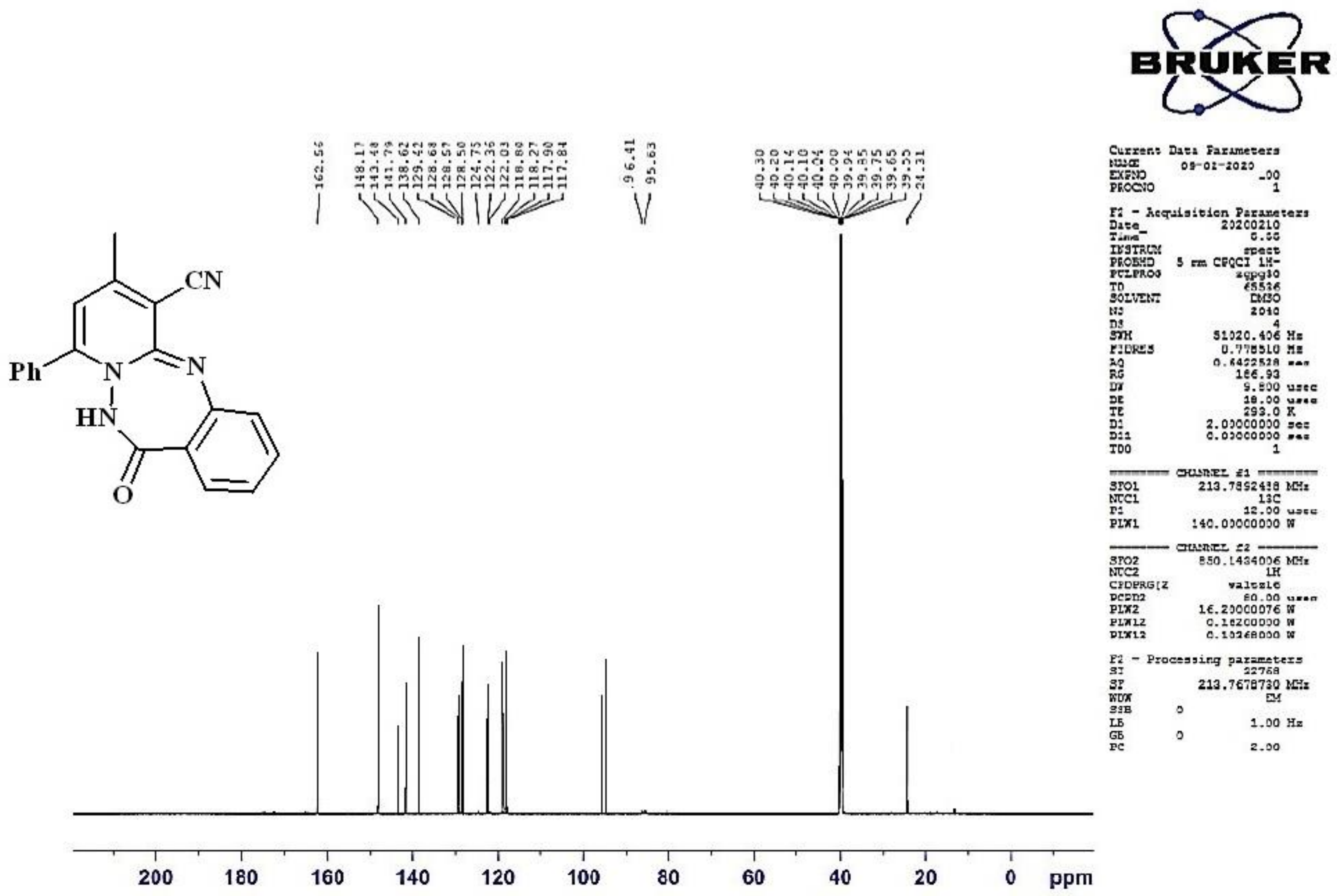

Supplementary fig. (9): ^{13}C NMR for compound **5**.

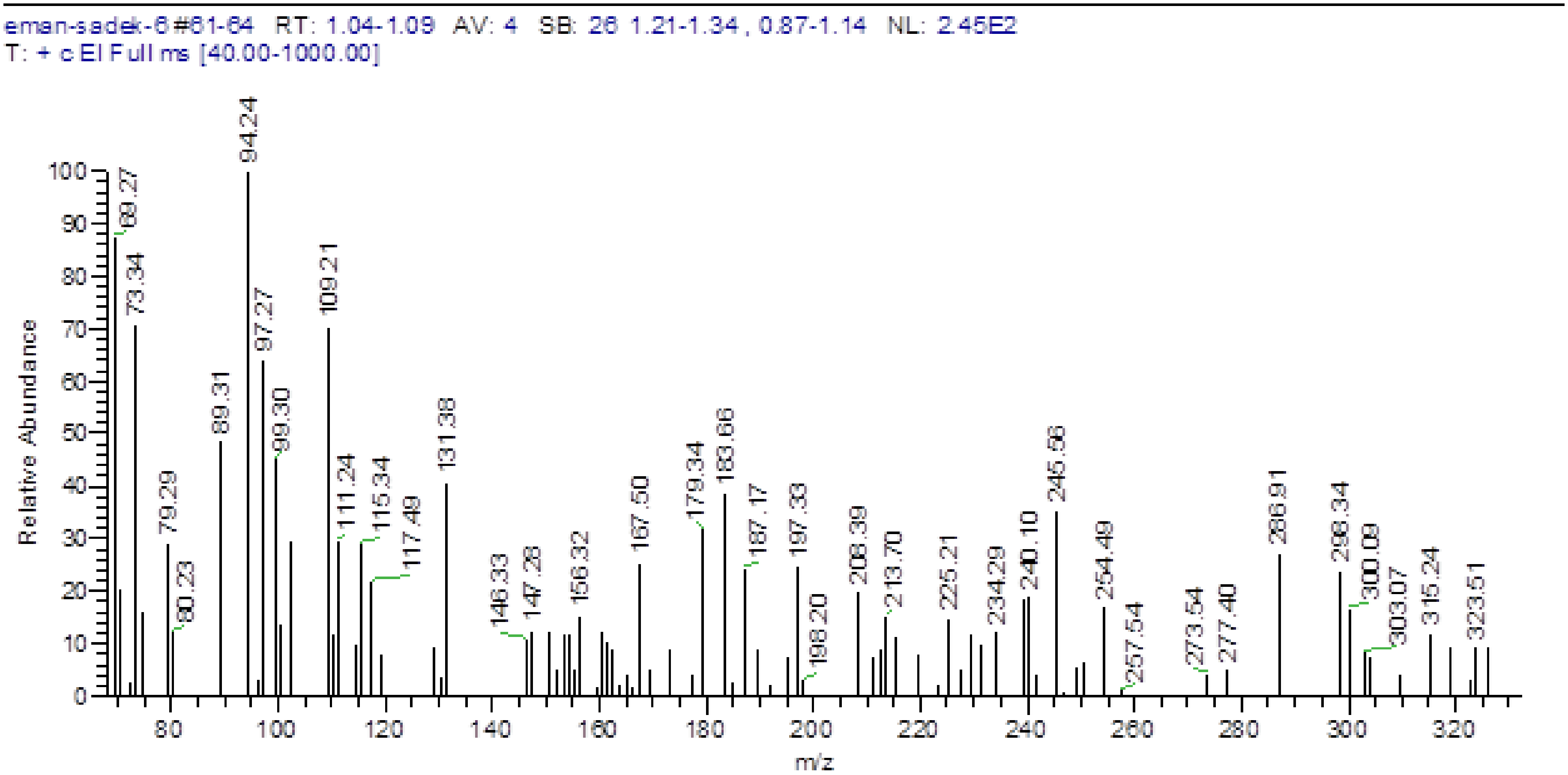

Supplementary fig. (10): mass spectrum for compound **5**.

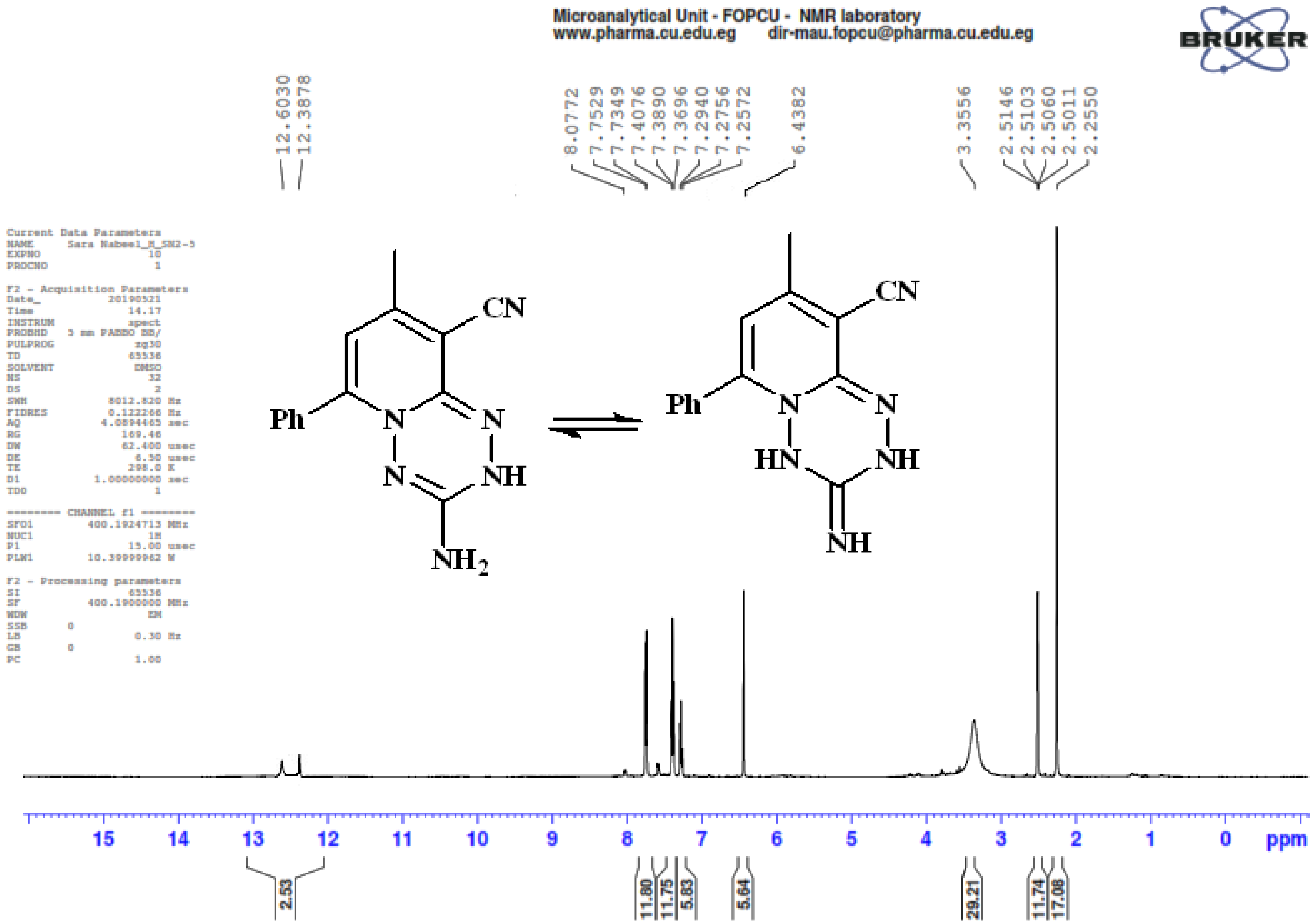

Supplementary fig. (11): ^{1}H NMR for compound **7**.

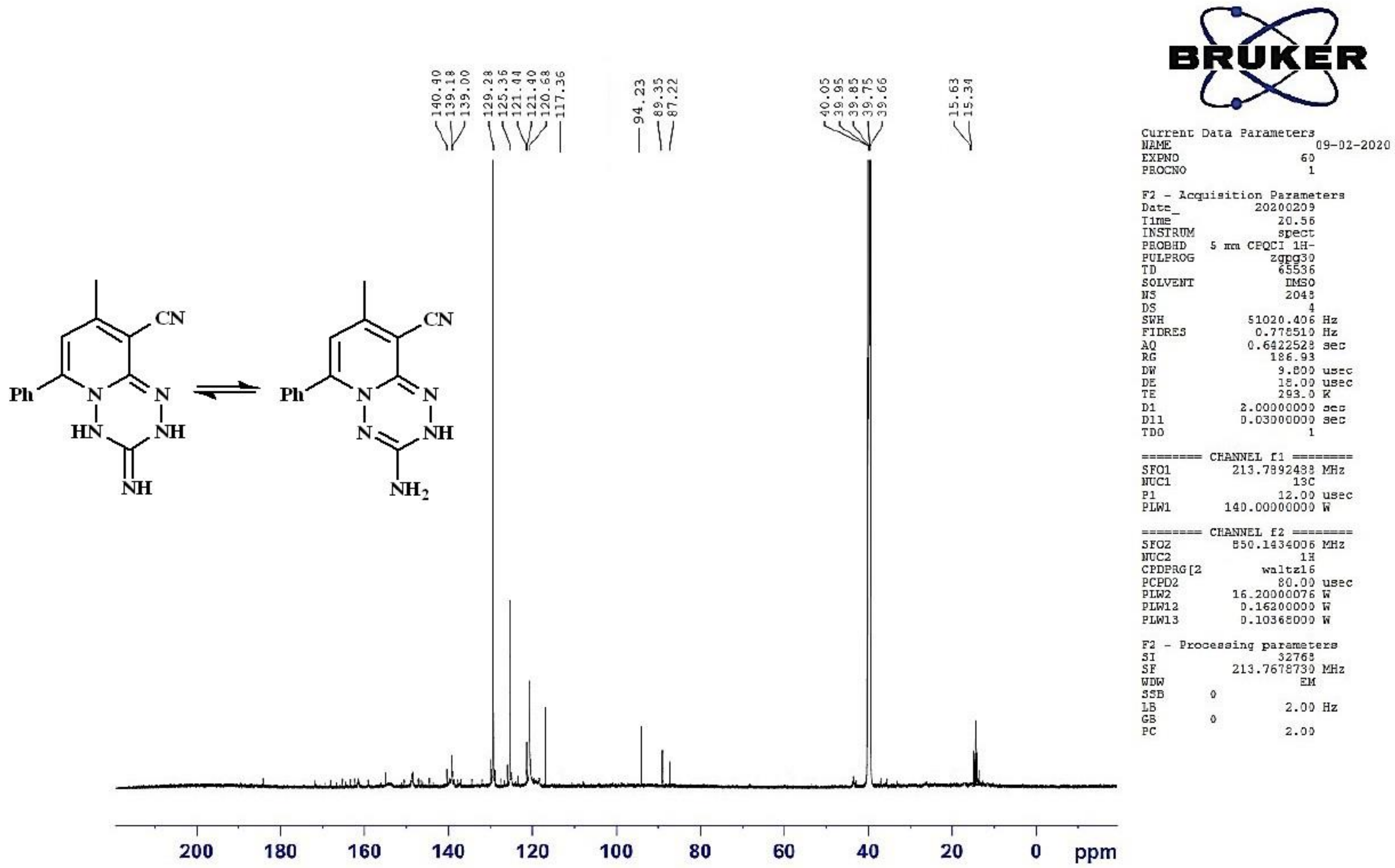

Supplementary fig. (12): ^{13}C NMR for compound **7**.

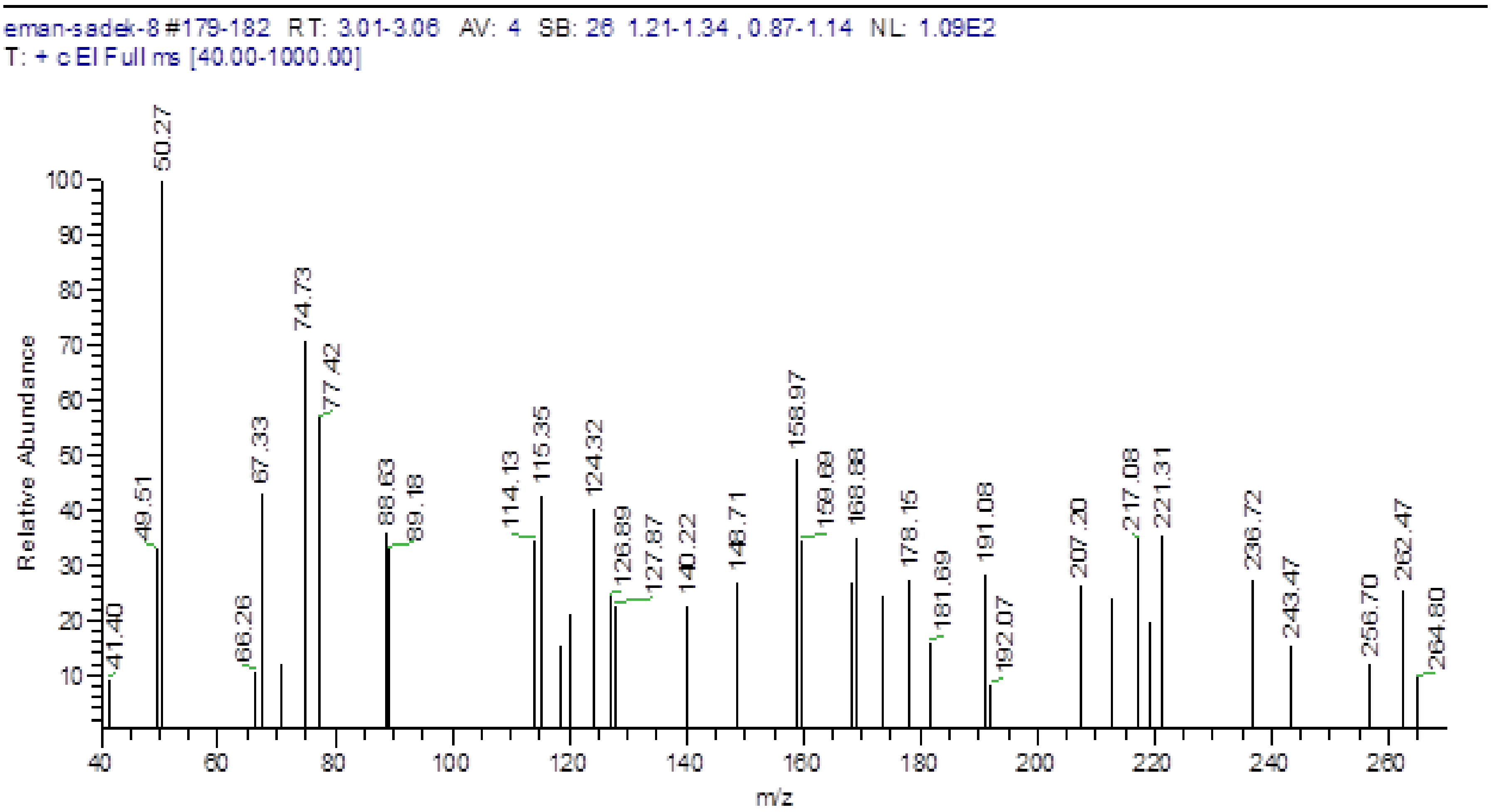

Supplementary fig. (13): mass spectrum for compound **7**.

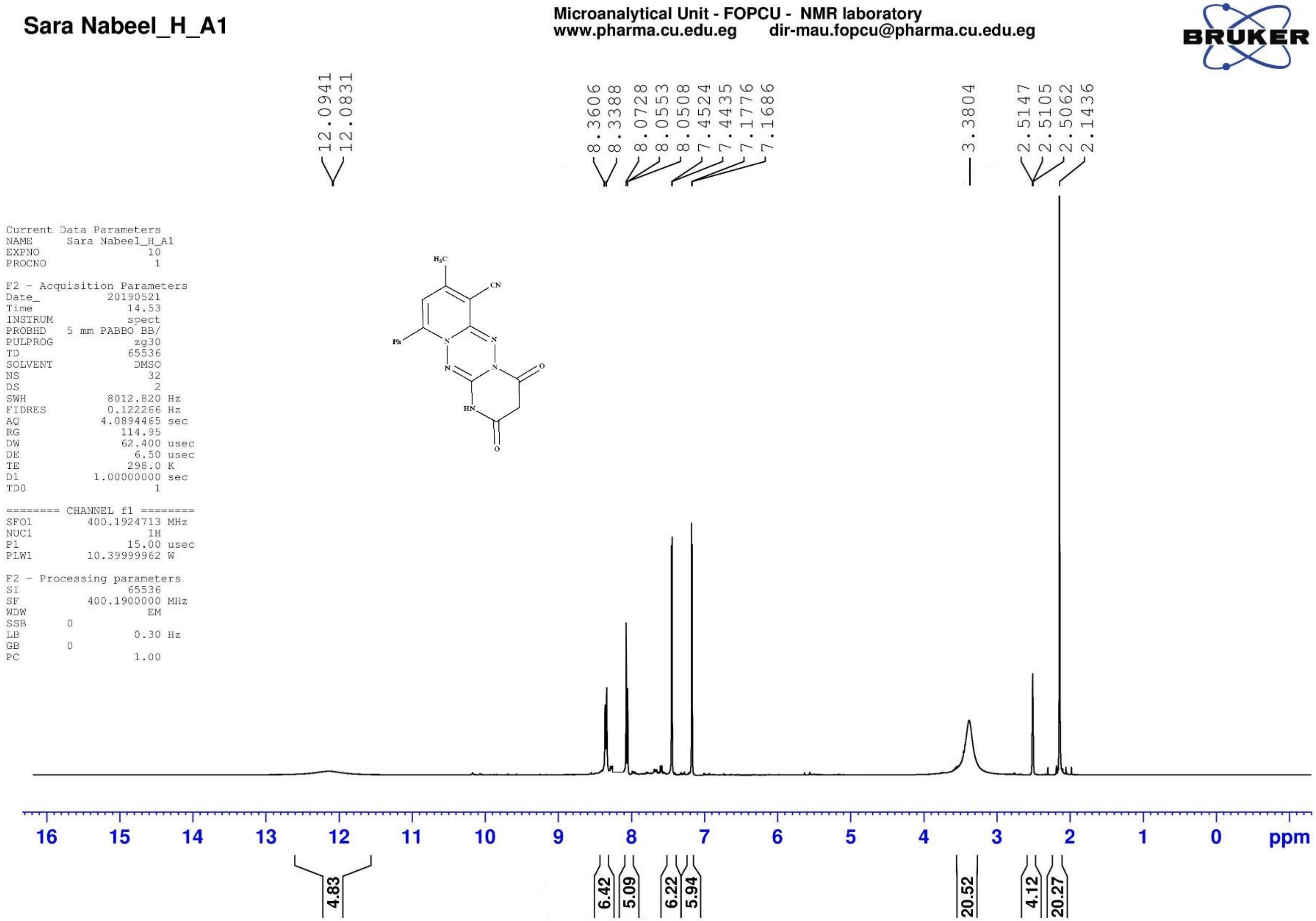

Supplementary fig. (14): ^{1}H NMR for compound **8**.

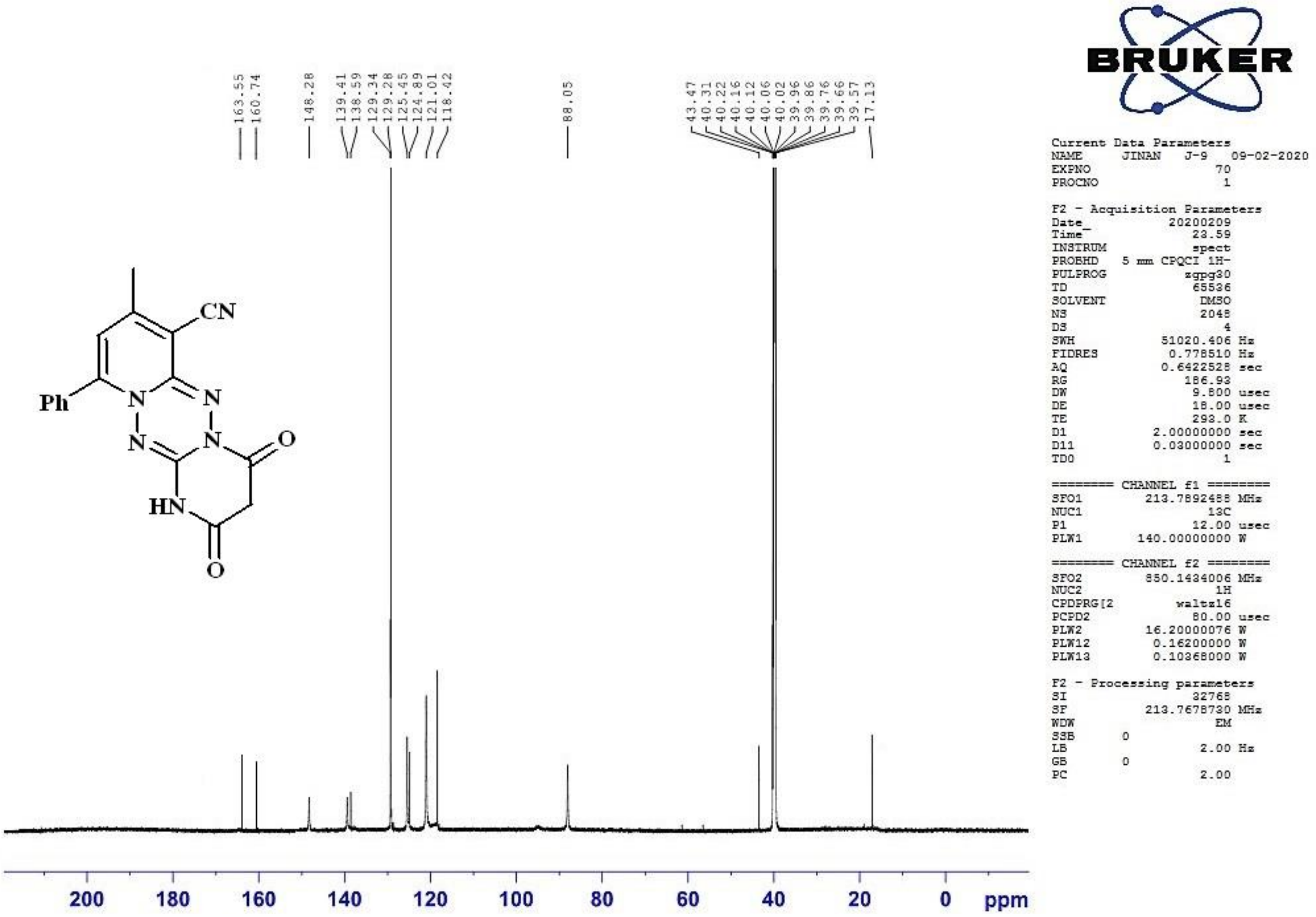

Supplementary fig. (15): ^{13}C NMR for compound **8**.

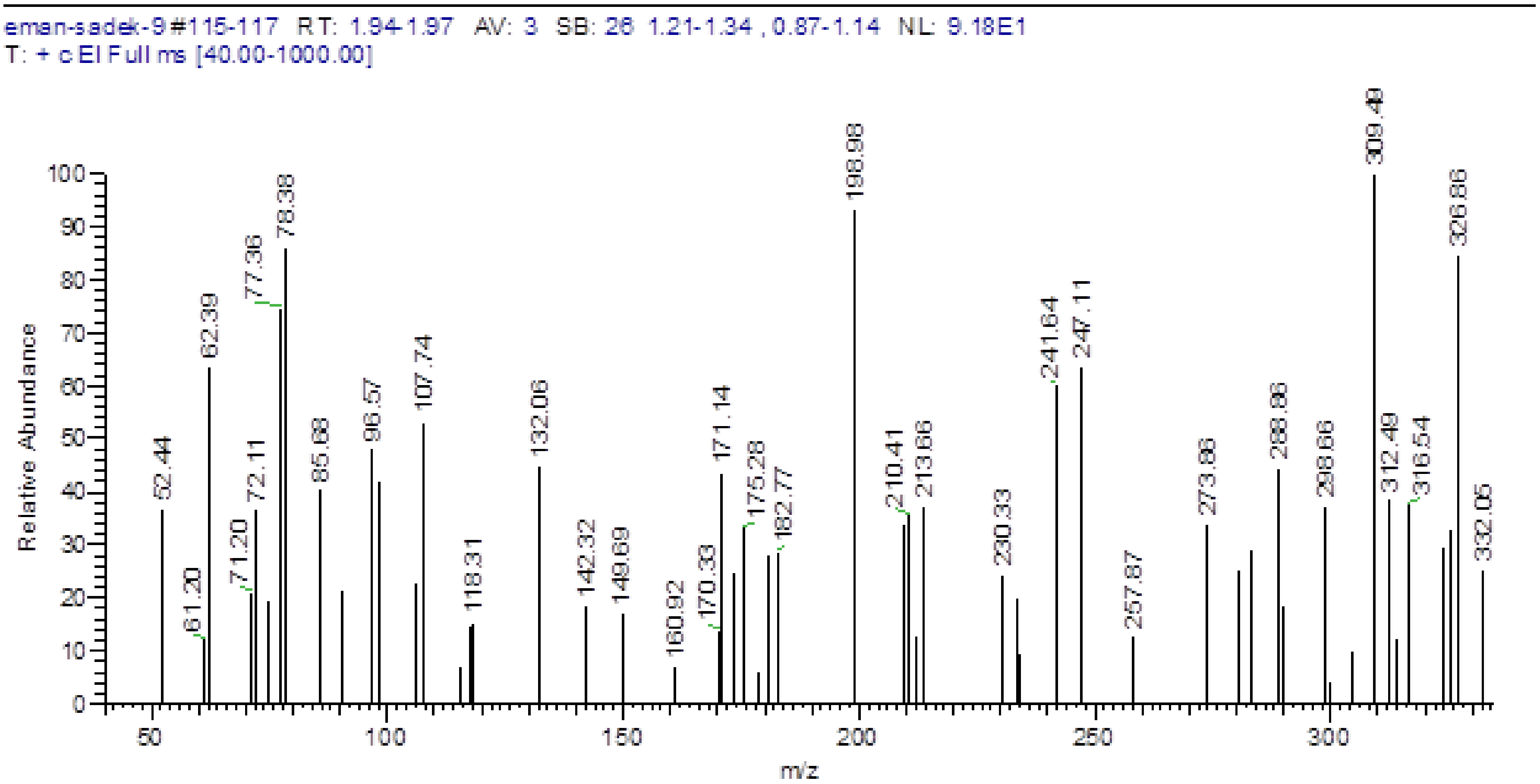

Supplementary fig. (16): mass spectrum for compound **8**.

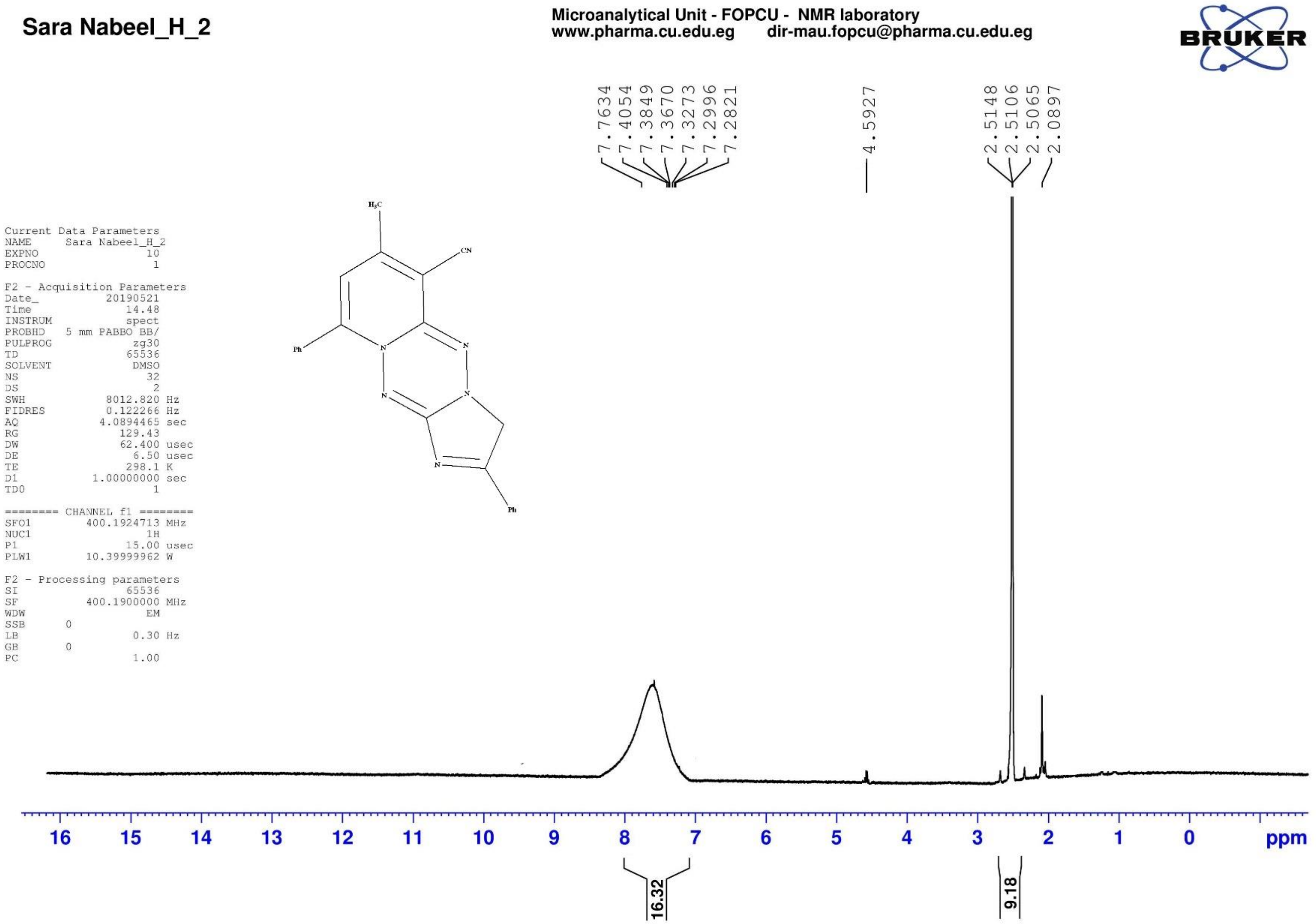

Supplementary fig. (17): ^{1}H NMR for compound **9**.

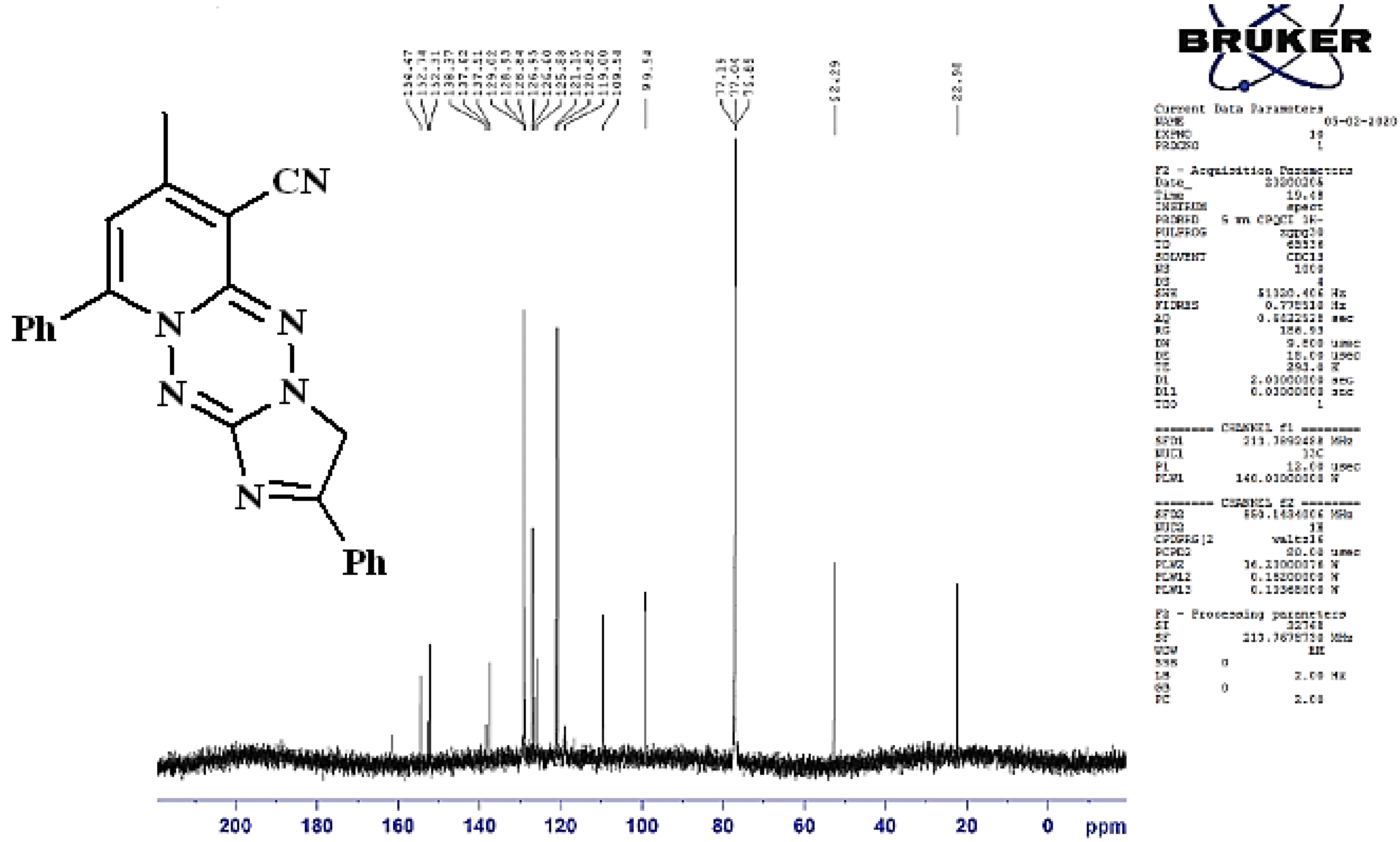

Supplementary fig. (18): ^{13}C NMR for compound **9**.

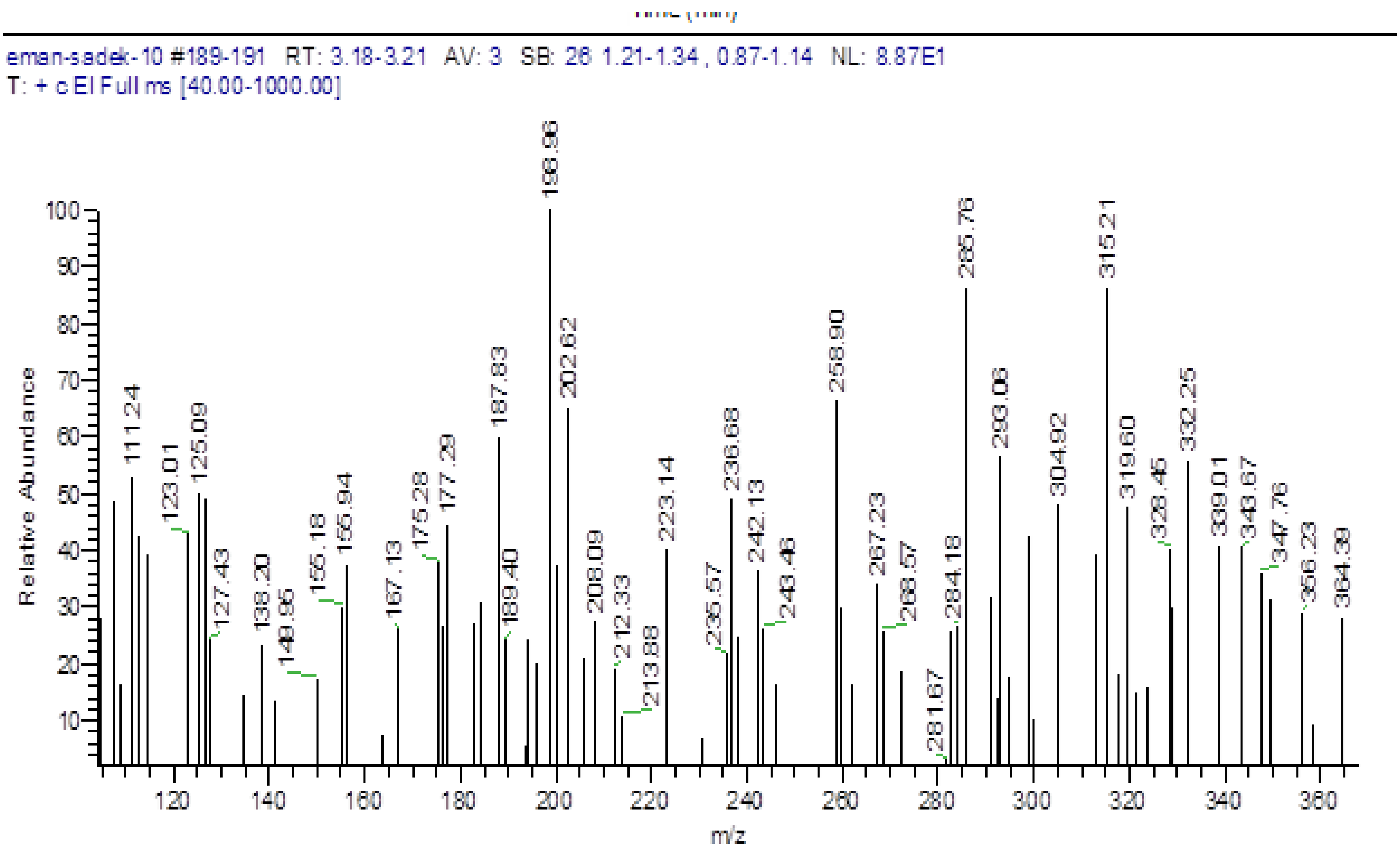

Supplementary fig. (19): mass spectrum for compound **9**.

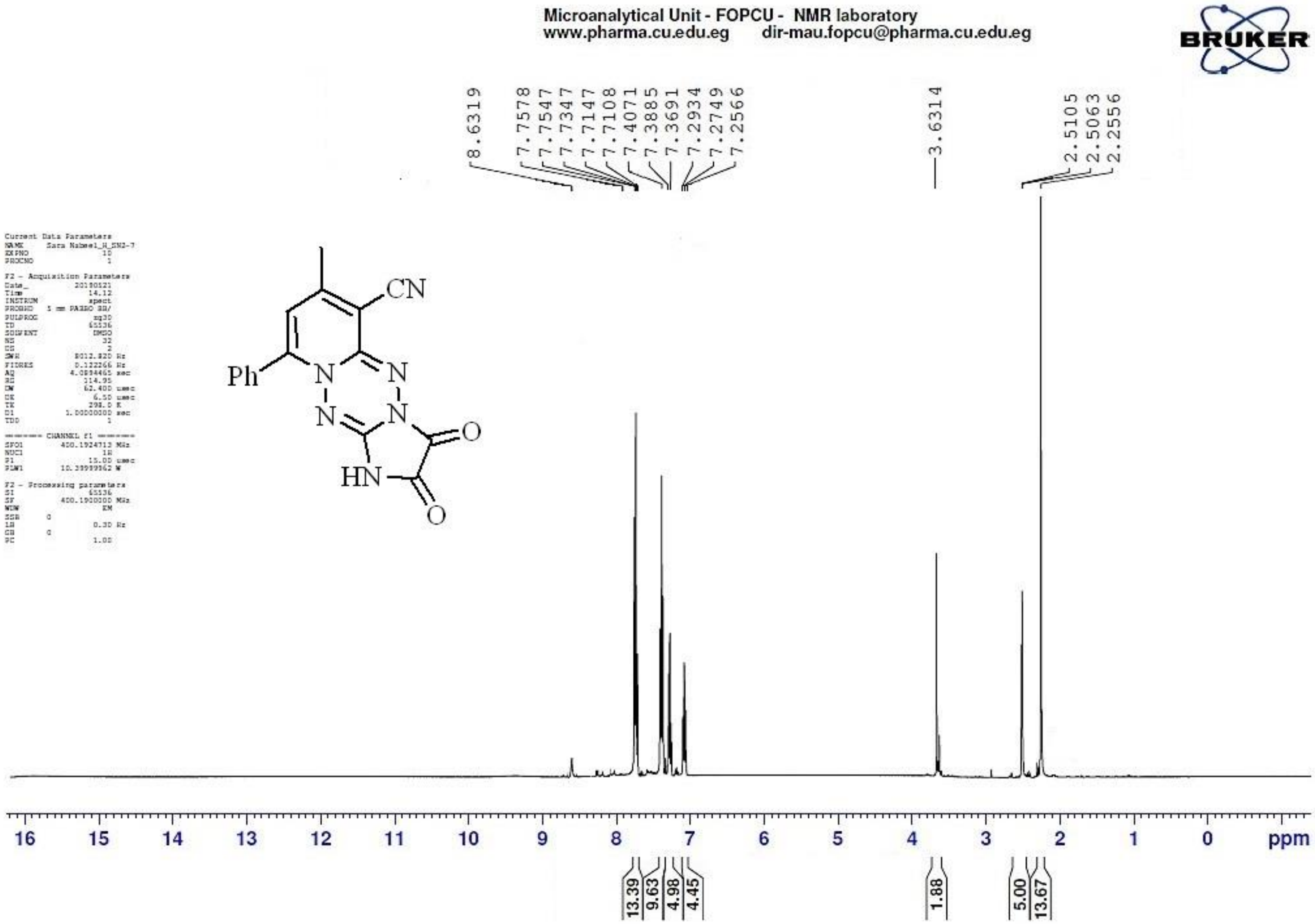

Supplementary fig. (20): ^{1}H NMR for compound **10**.

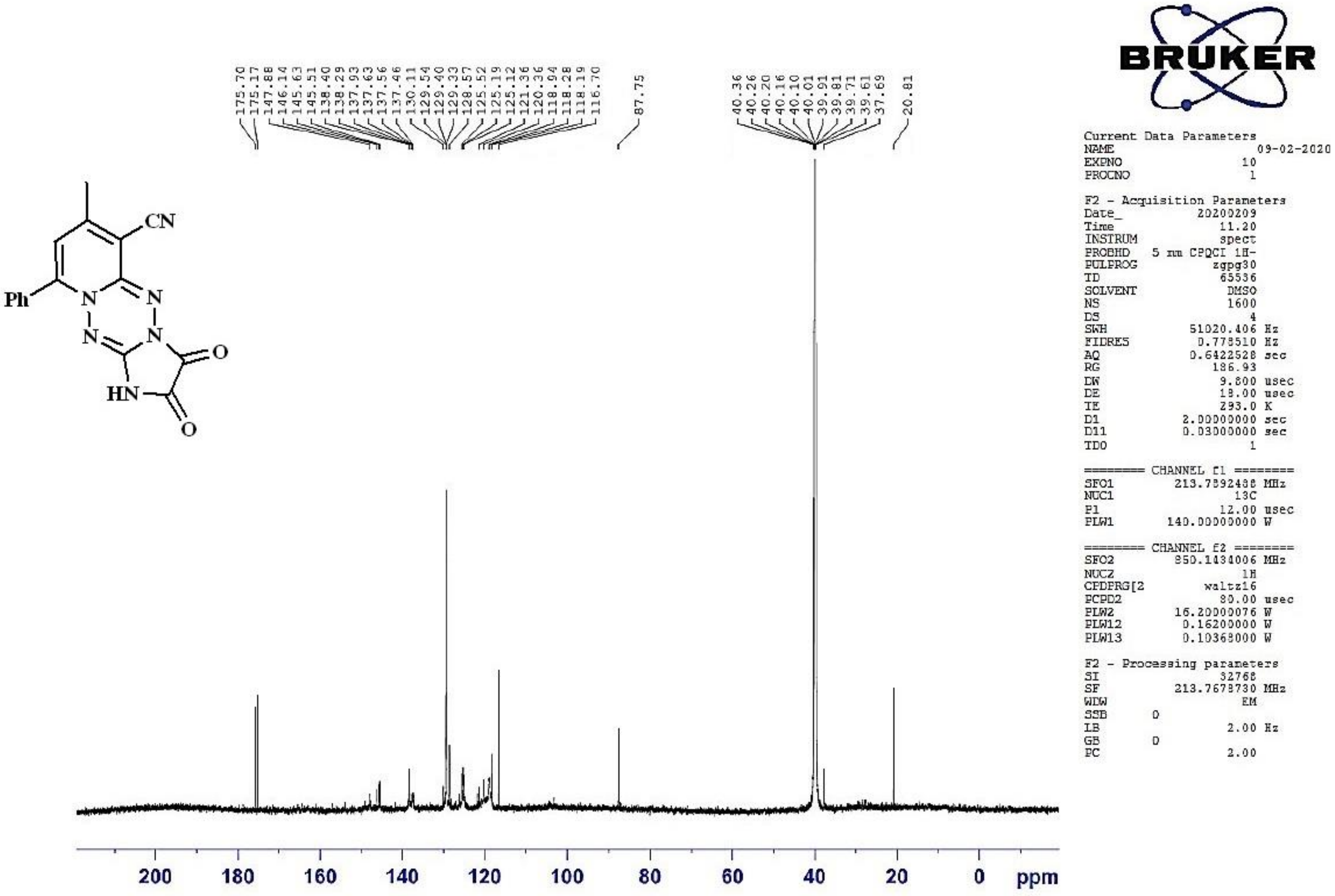

Supplementary fig. (21): ^{13}C NMR for compound **10**.

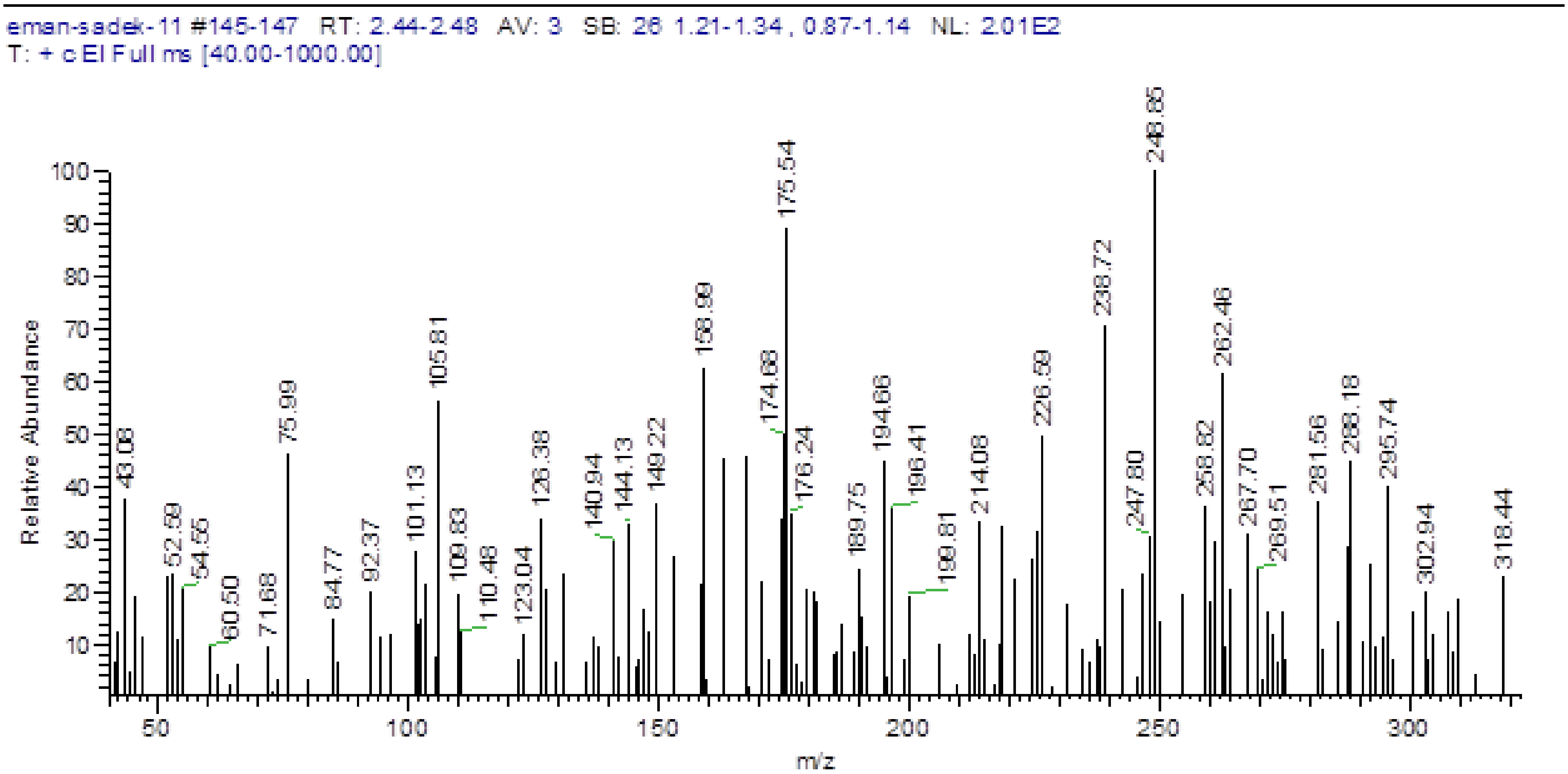

Supplementary fig. (22): mass spectrum for compound **10**.

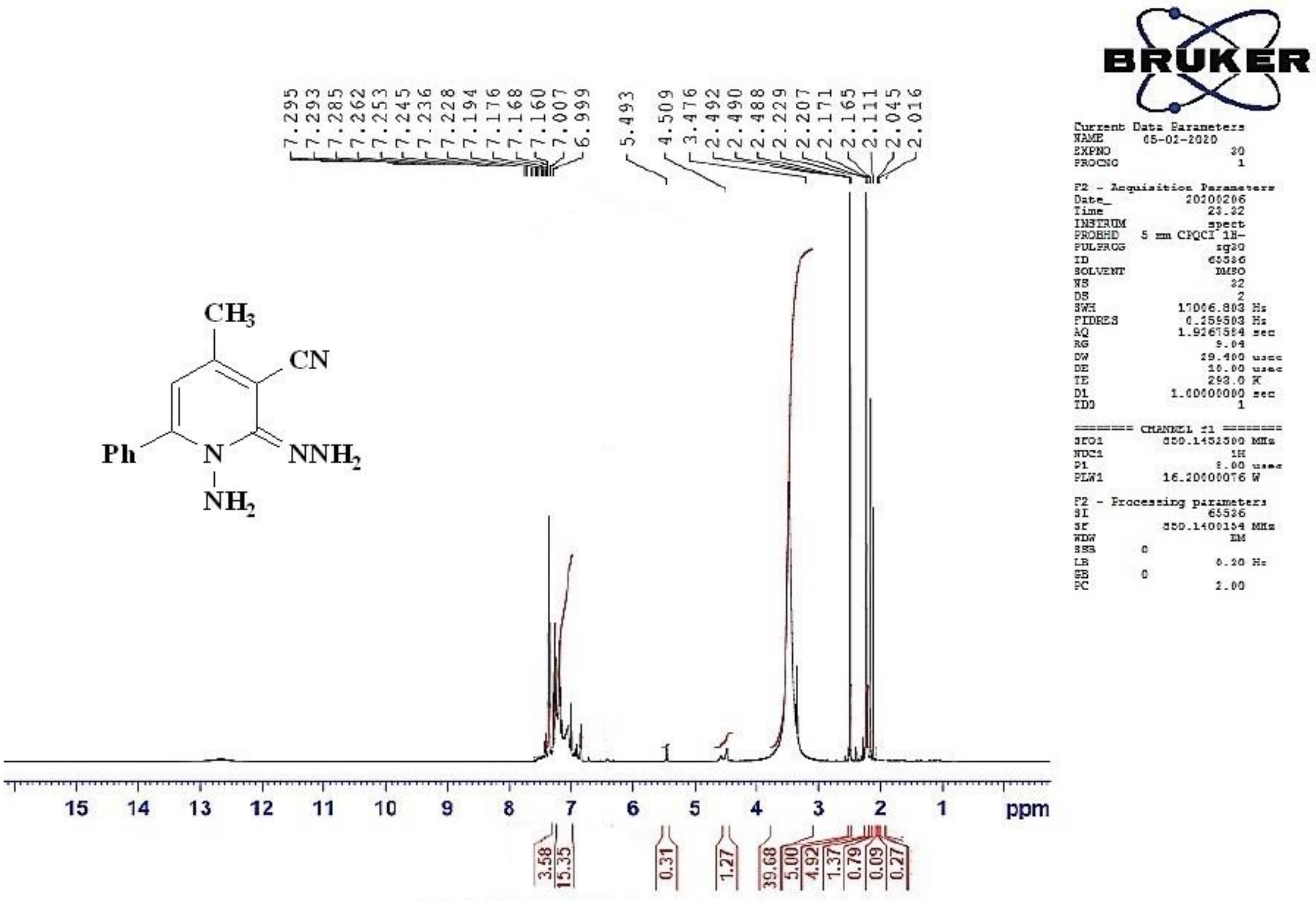

Supplementary fig. (23): ^{1}H NMR for compound **11**.

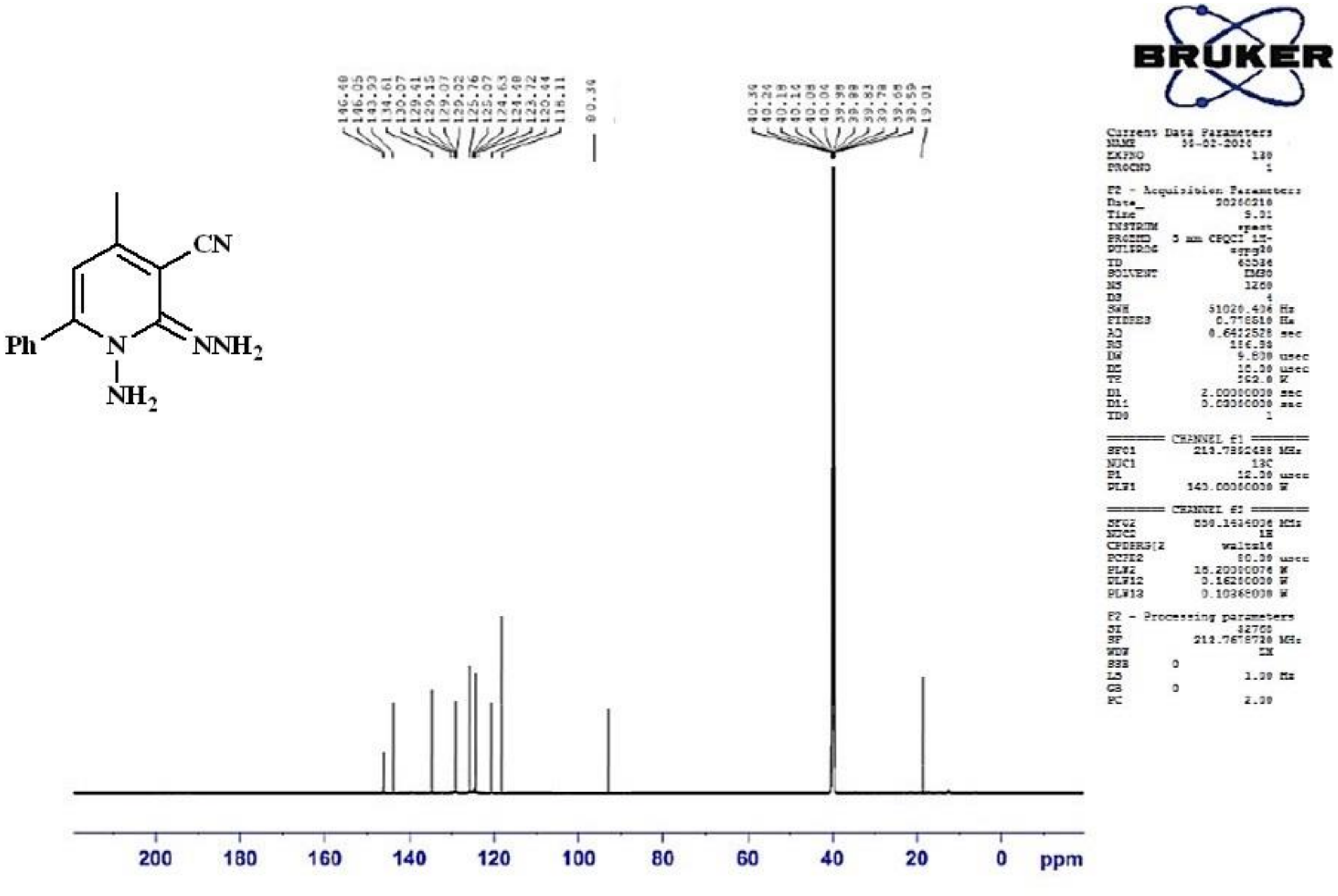

Supplementary fig. (24): ^{13}C NMR for compound **11**.

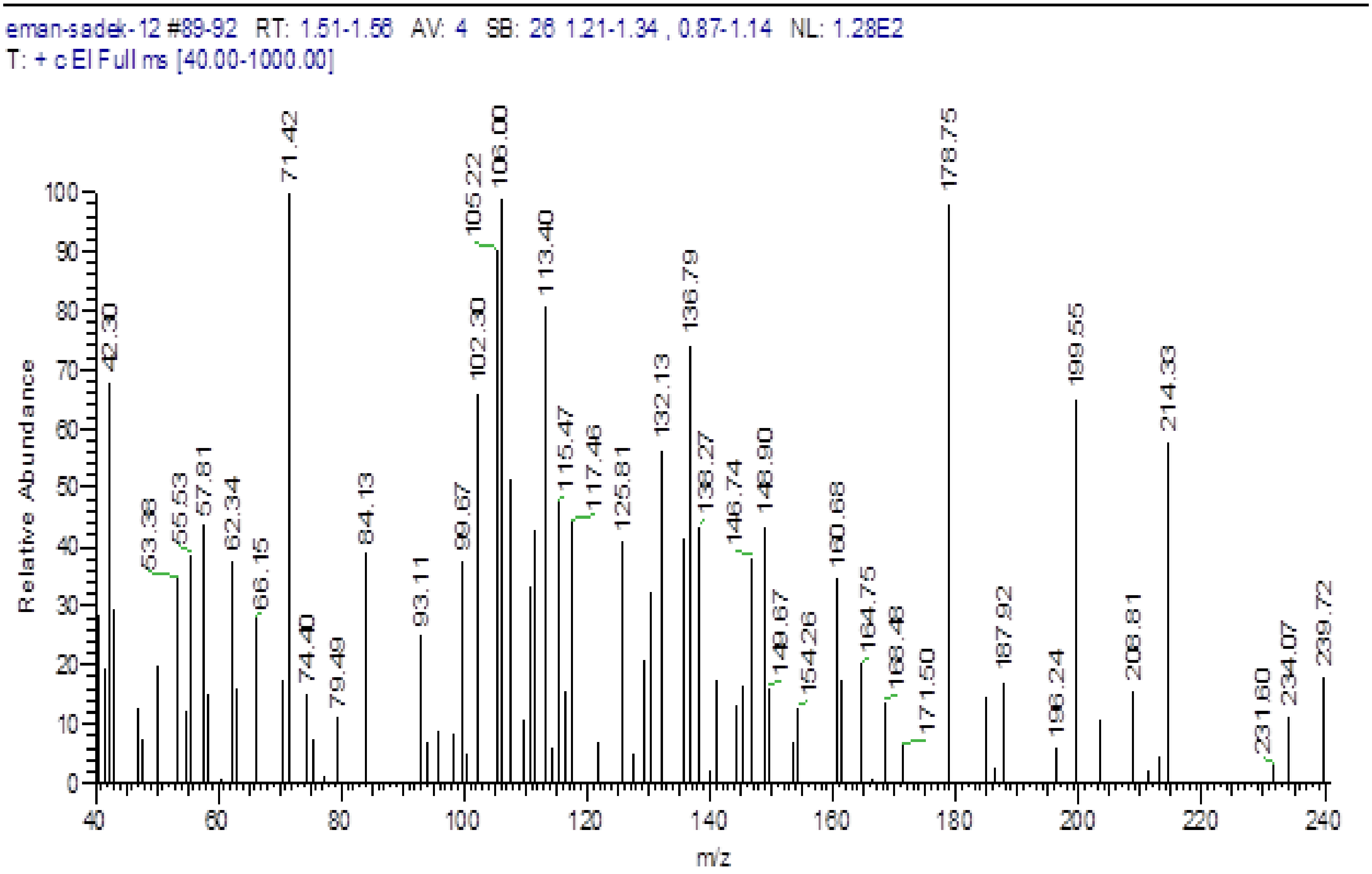

Supplementary fig. (25): mass spectrum for compound **11**

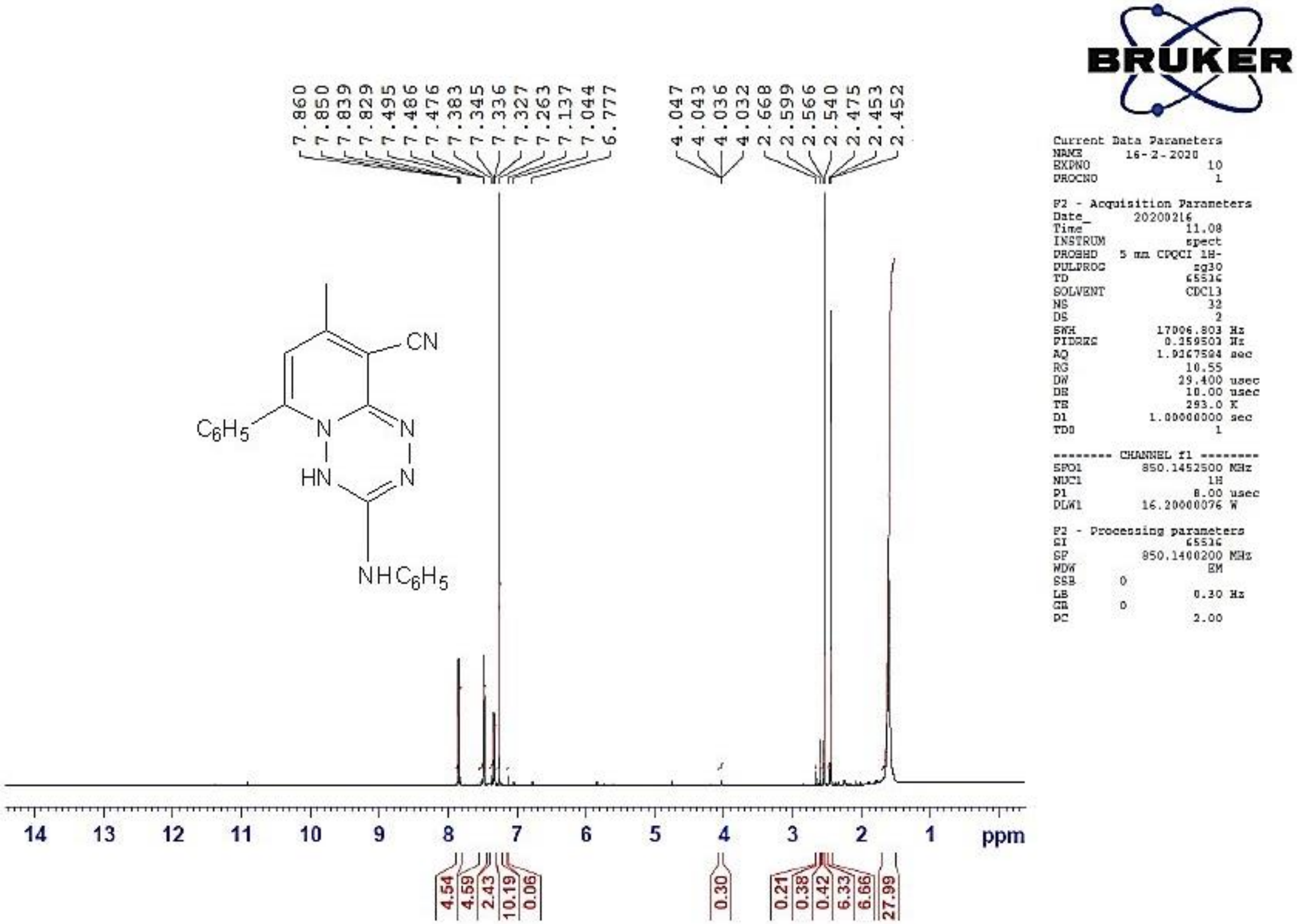

Supplementary fig. (26): ^{1}H NMR for compound **12**.

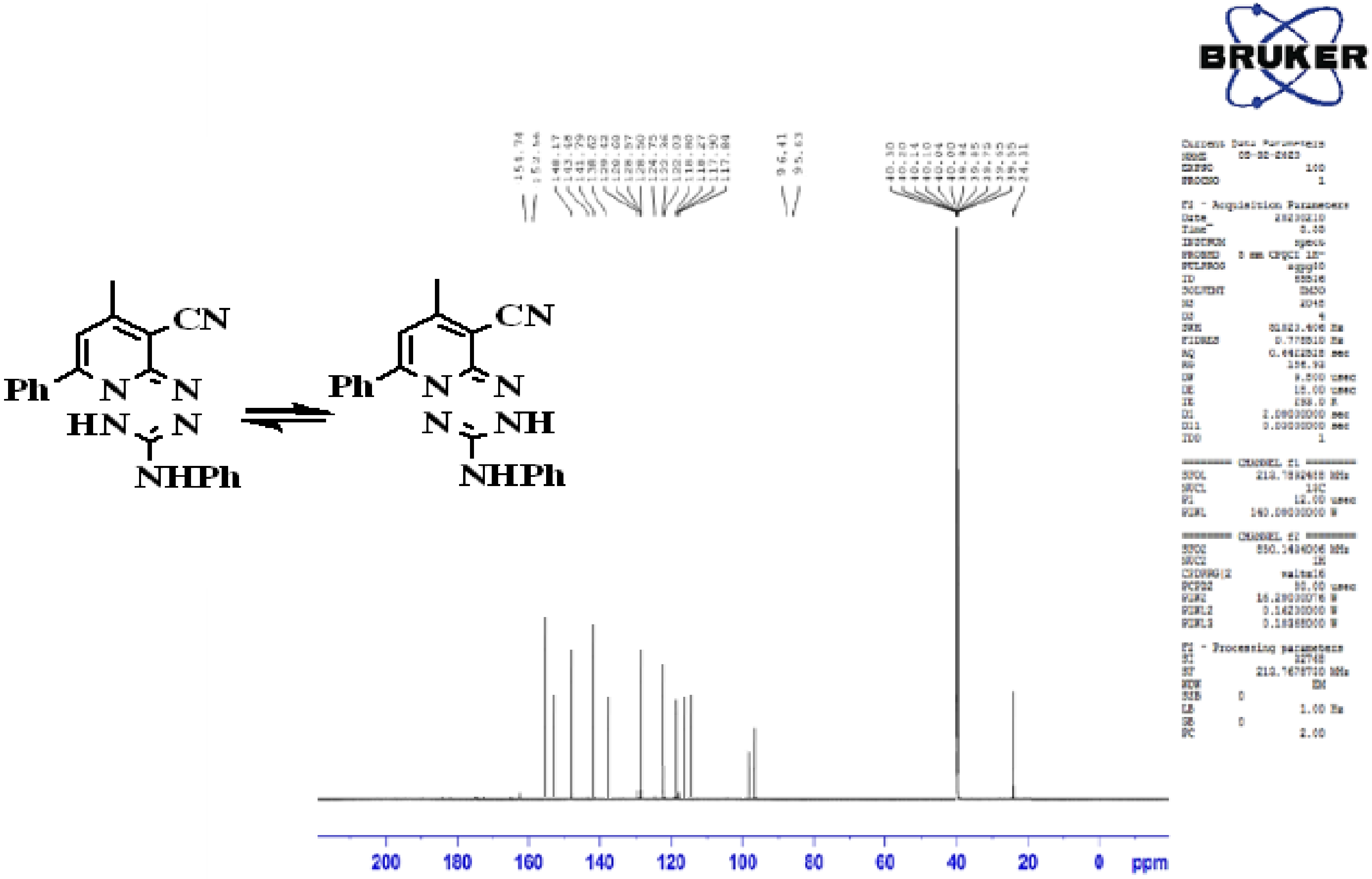

Supplementary fig. (27): ^{13}C NMR for compound **12**.

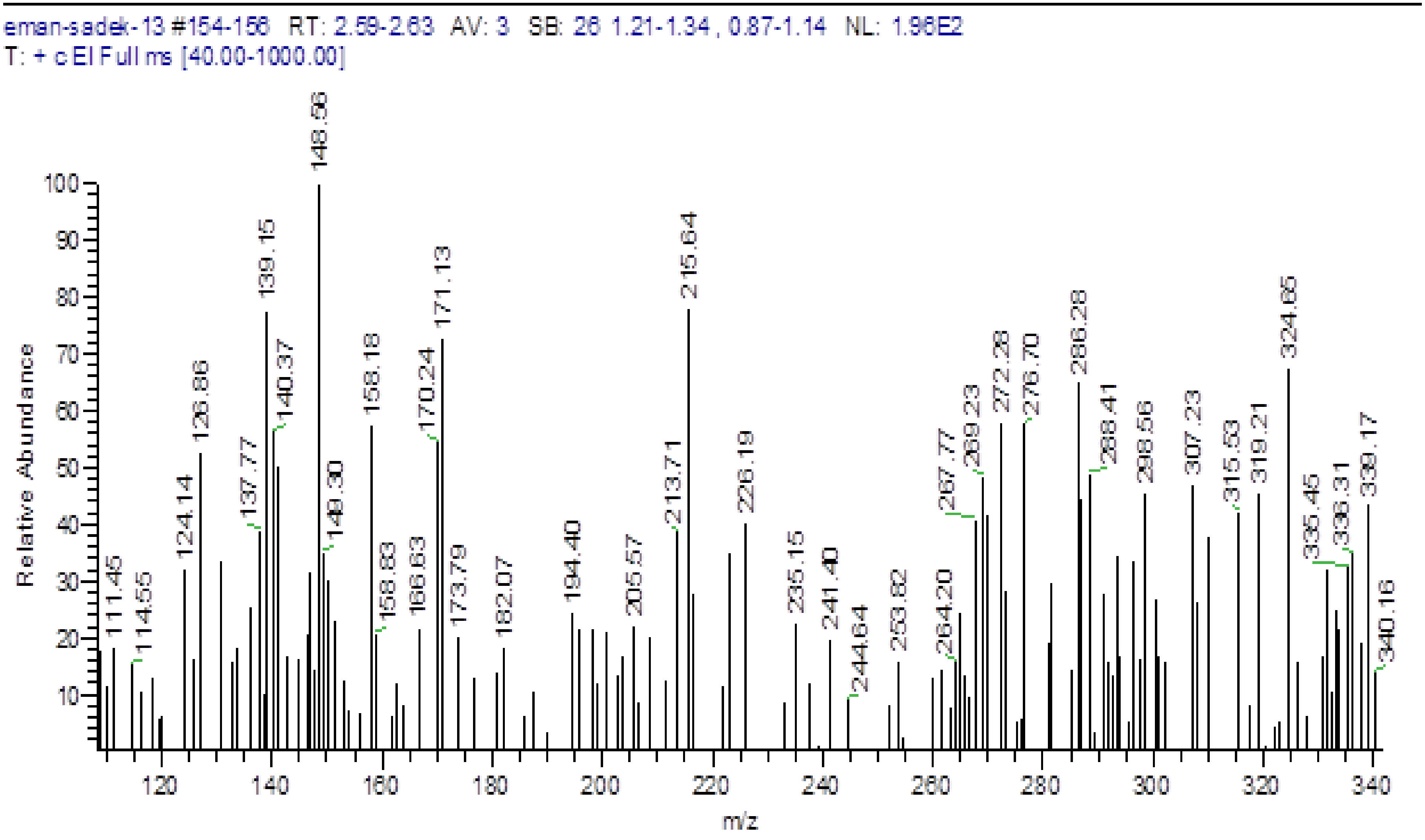

Supplementary fig. (28): mass spectrum for compound **12**.

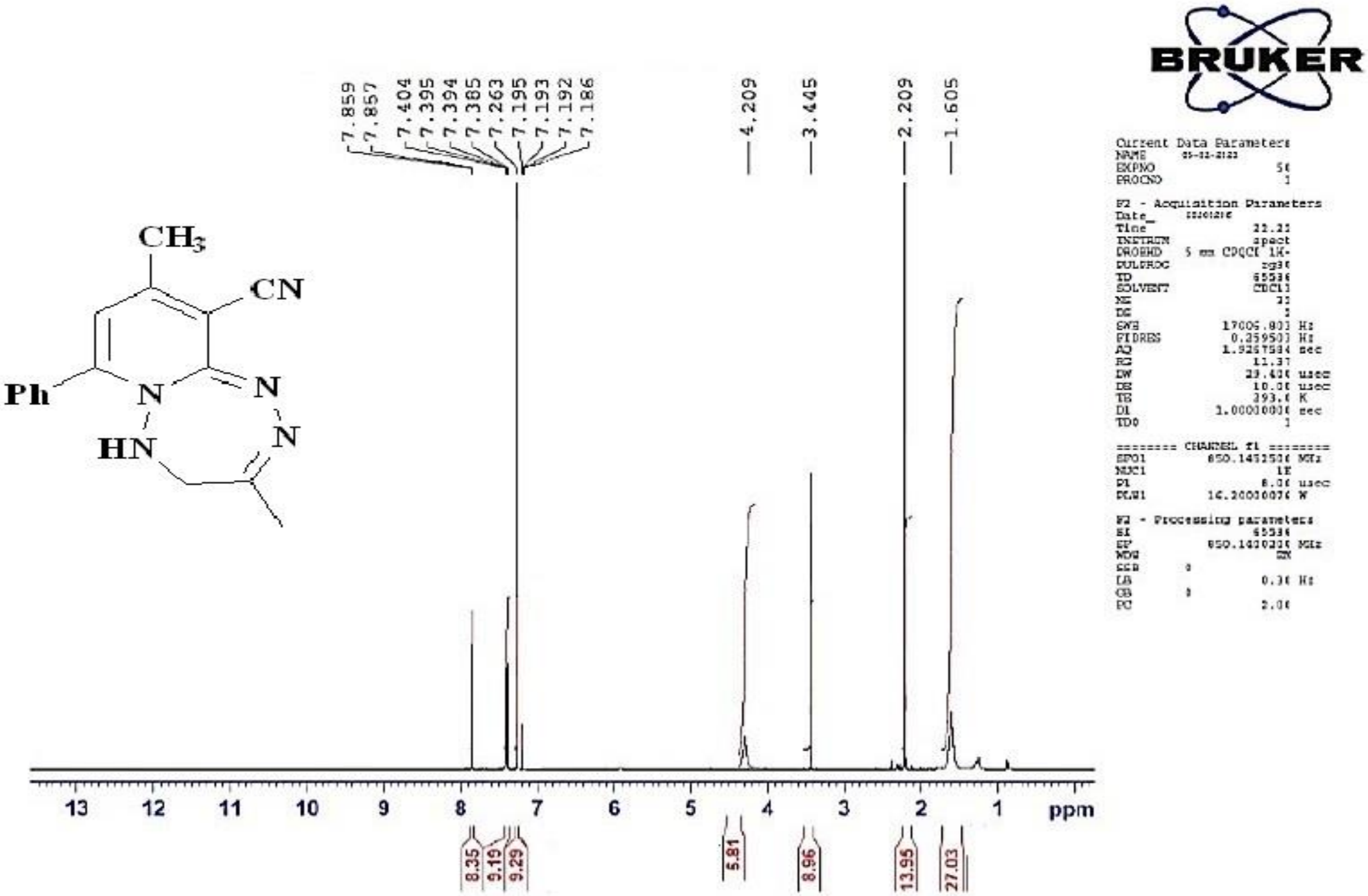

Supplementary fig. (29): ^{1}H NMR for compound **13**.

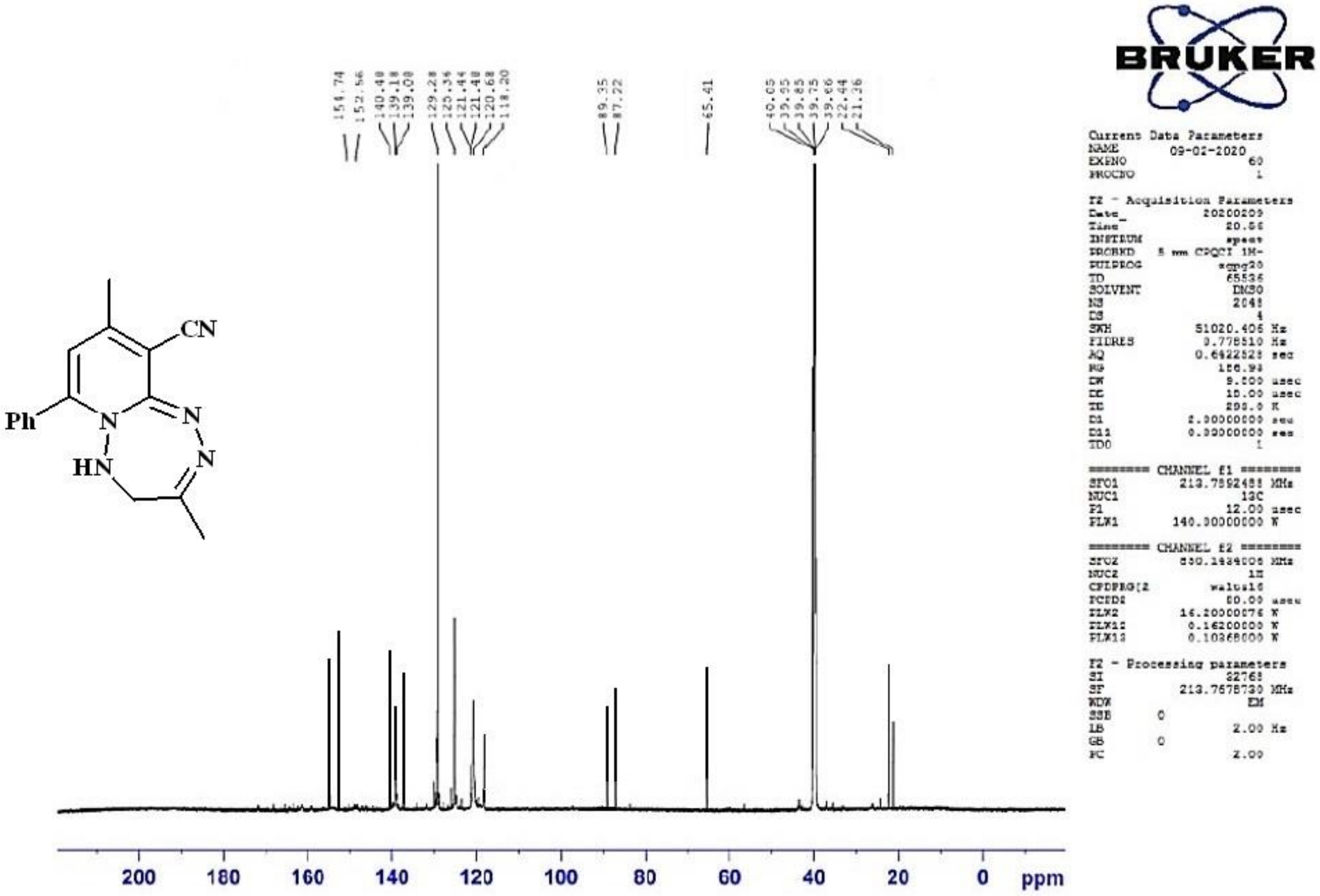

Supplementary fig. (30): ^{13}C NMR for compound **13**.

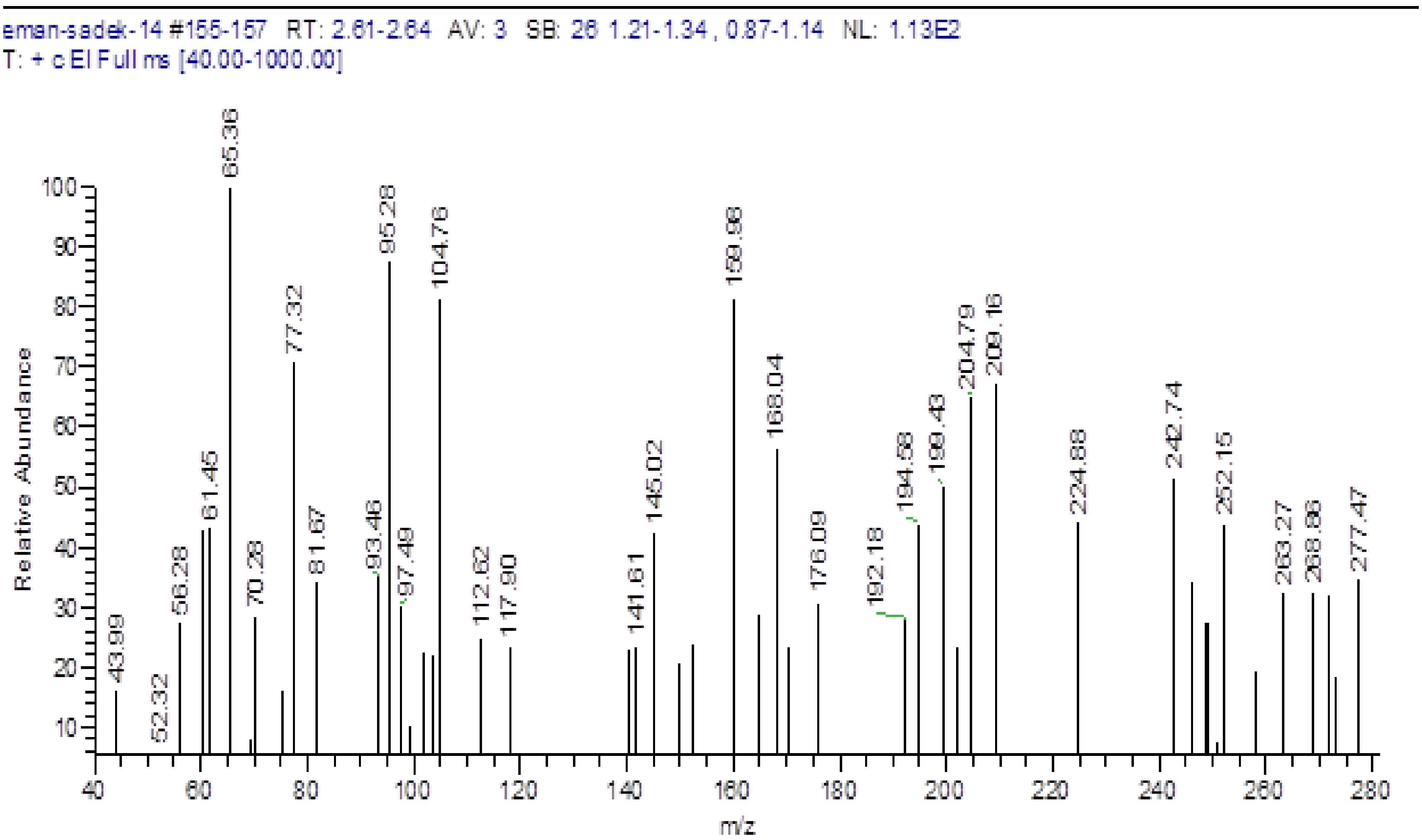

Supplementary fig. (31): mass spectrum for compound **13**.

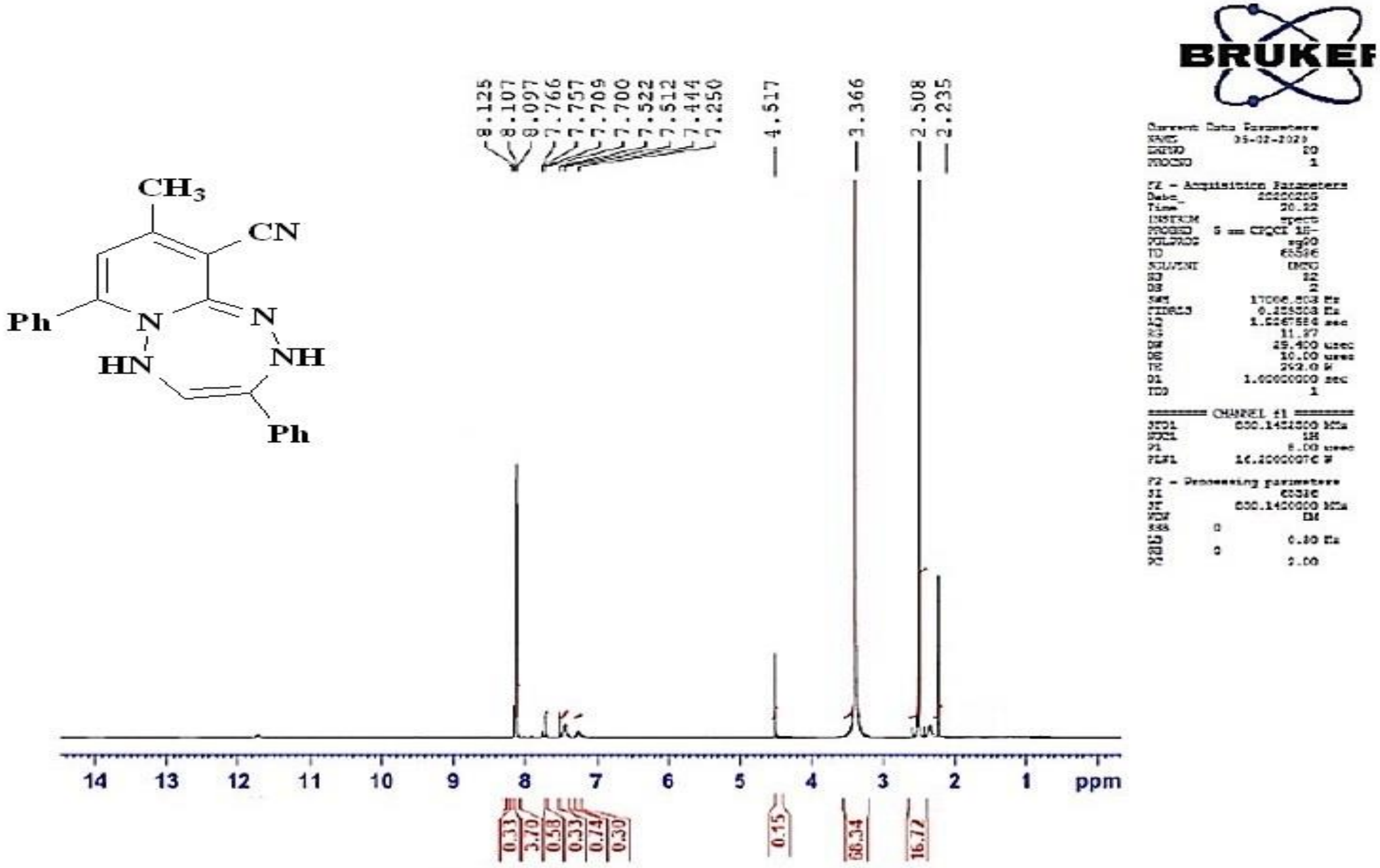

Supplementary fig. (32): ^{1}H NMR for compound **14**.

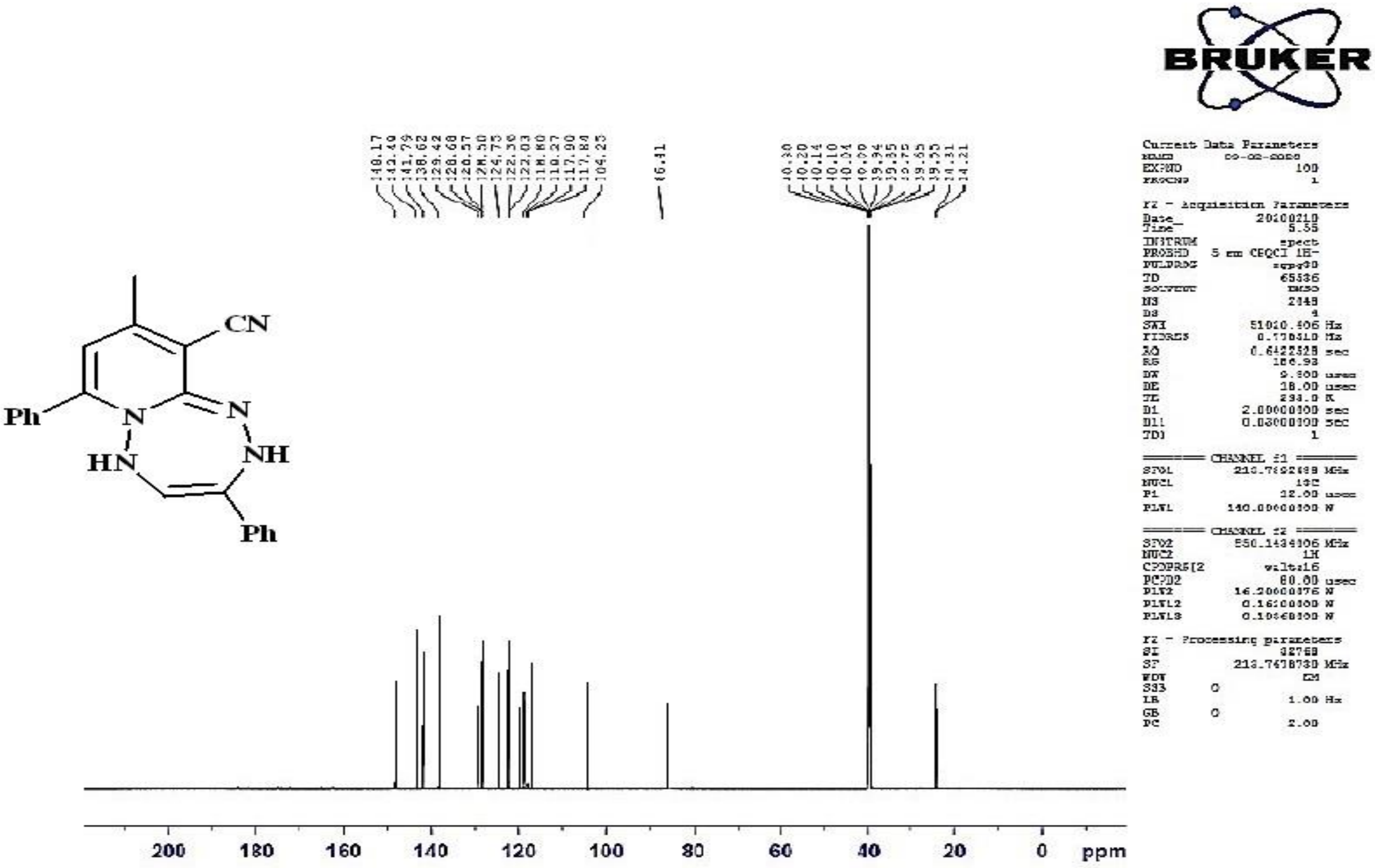

Supplementary fig. (33): ^{13}C NMR for compound **14**.

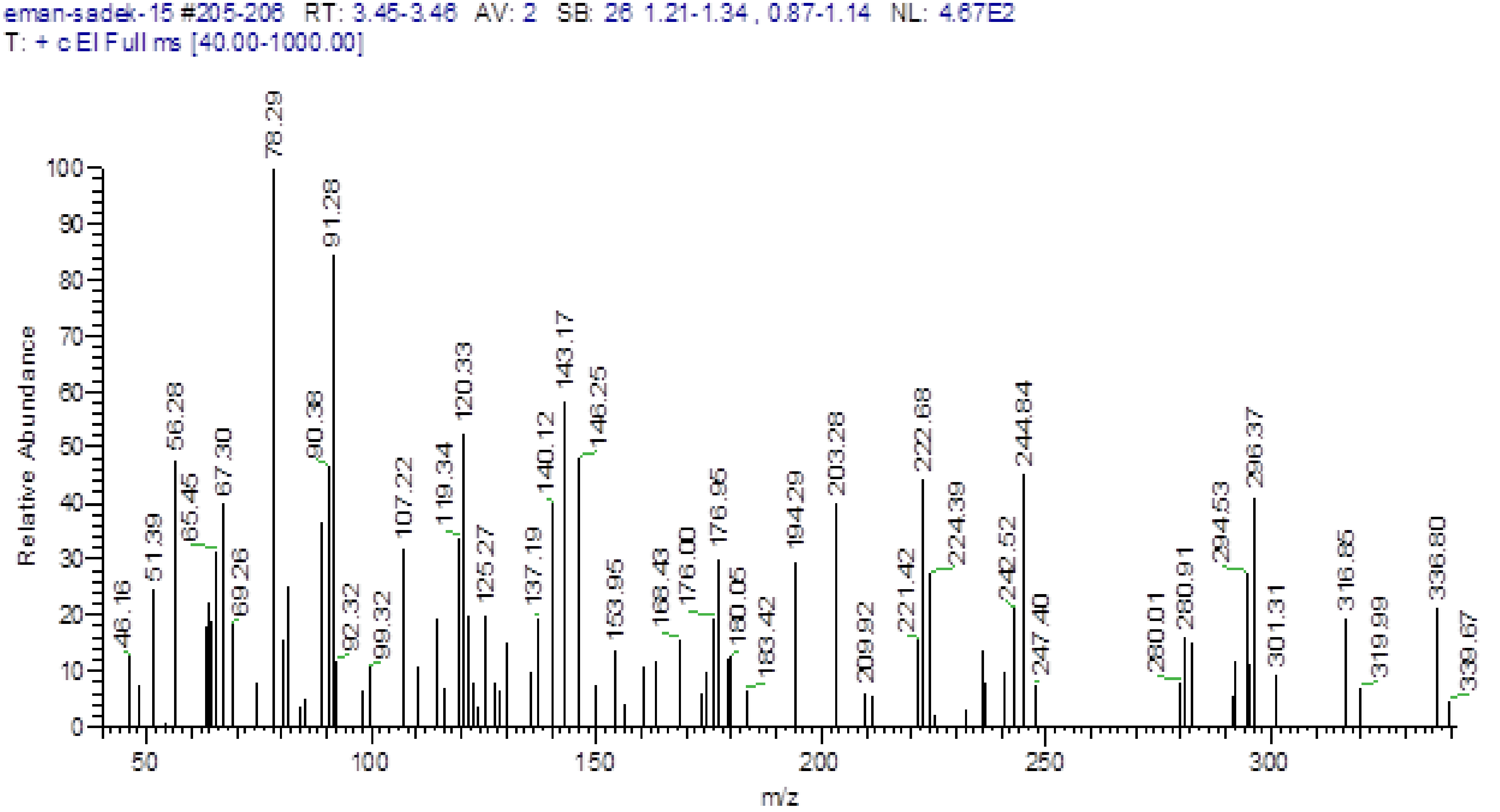

Supplementary fig. (34): mass spectrum for compound **14**.

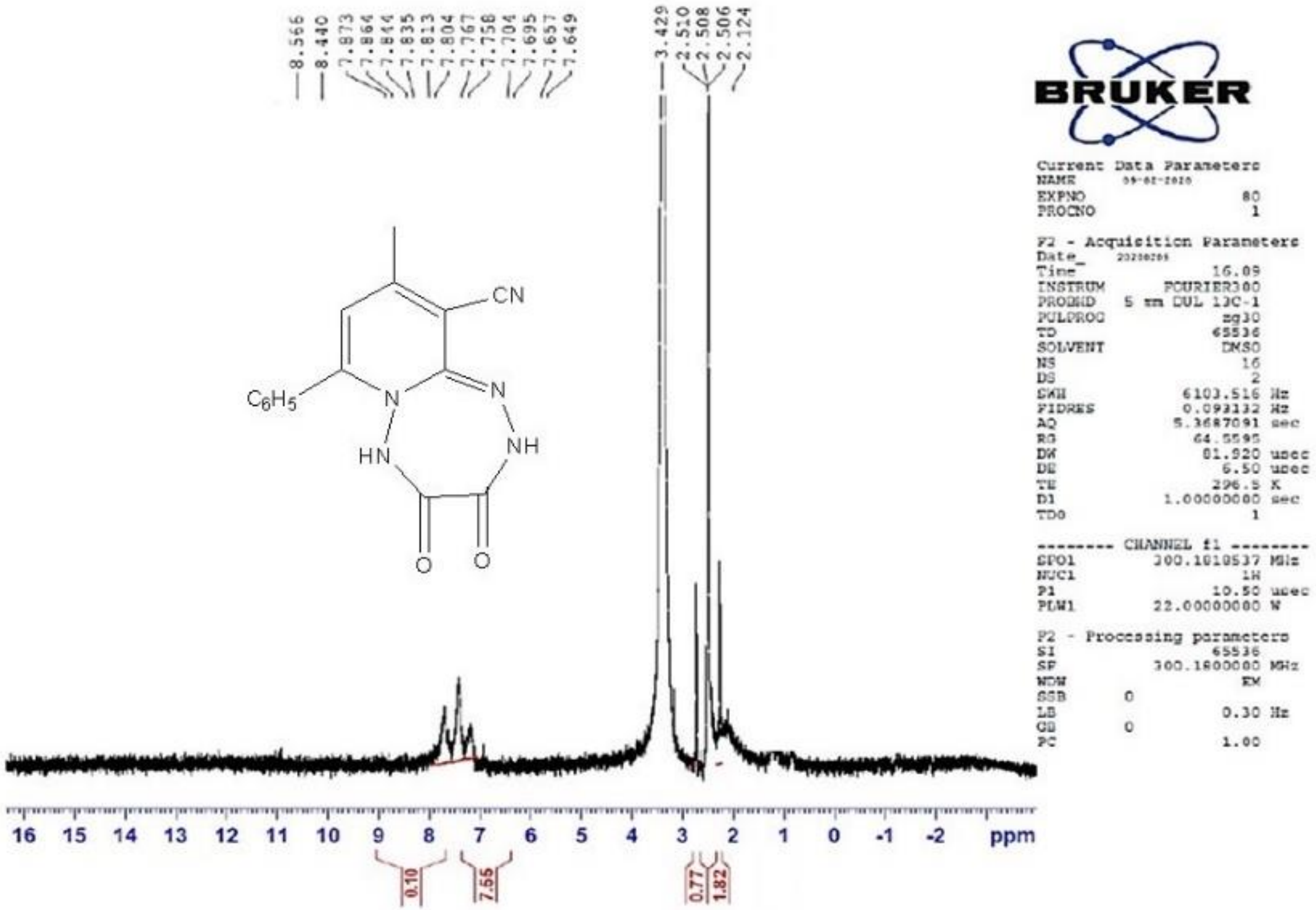

Supplementary fig. (35): ^{1}H NMR for compound **15**.

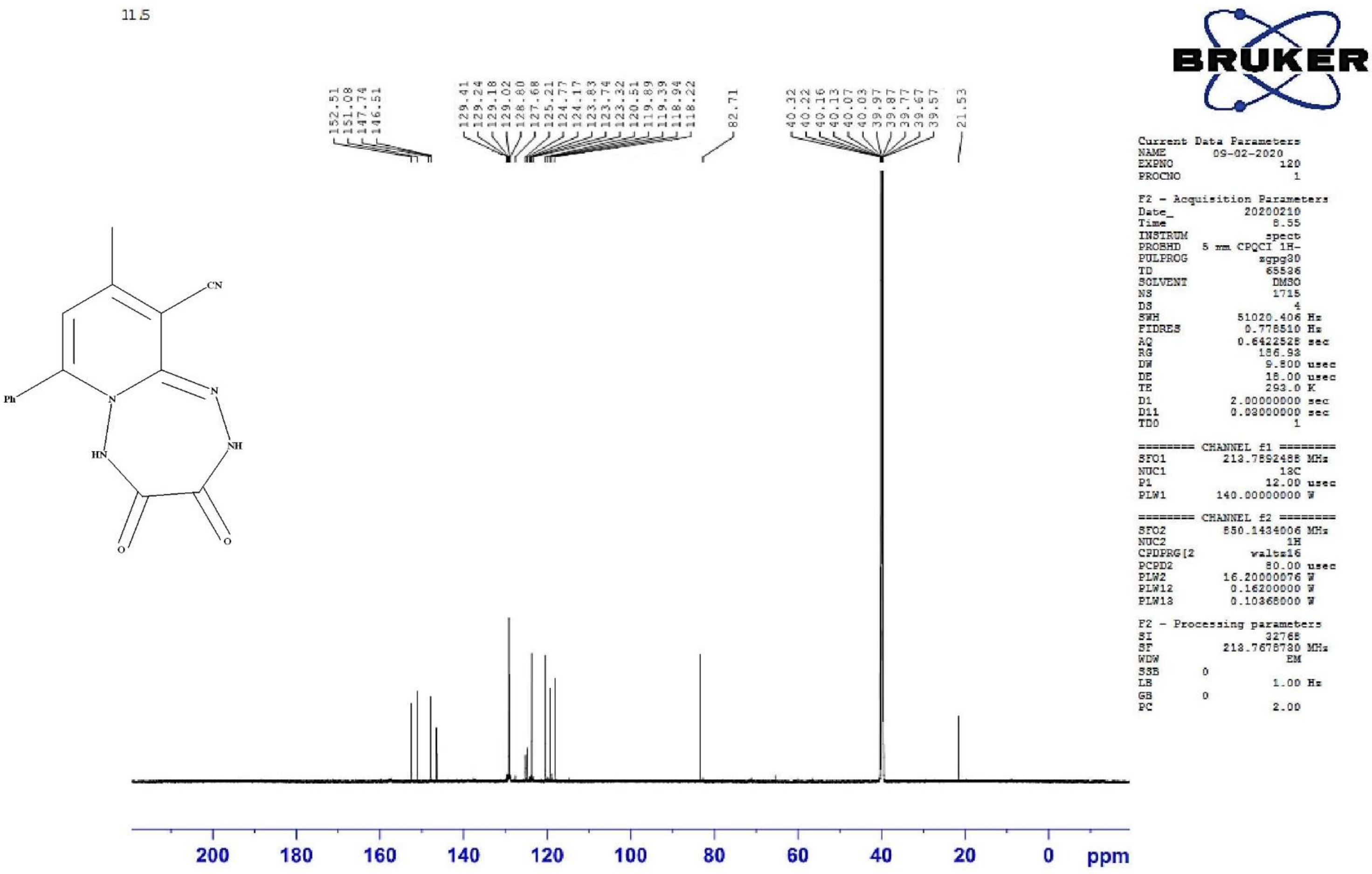

Supplementary fig. (36): ^{13}C NMR for compound **15**.

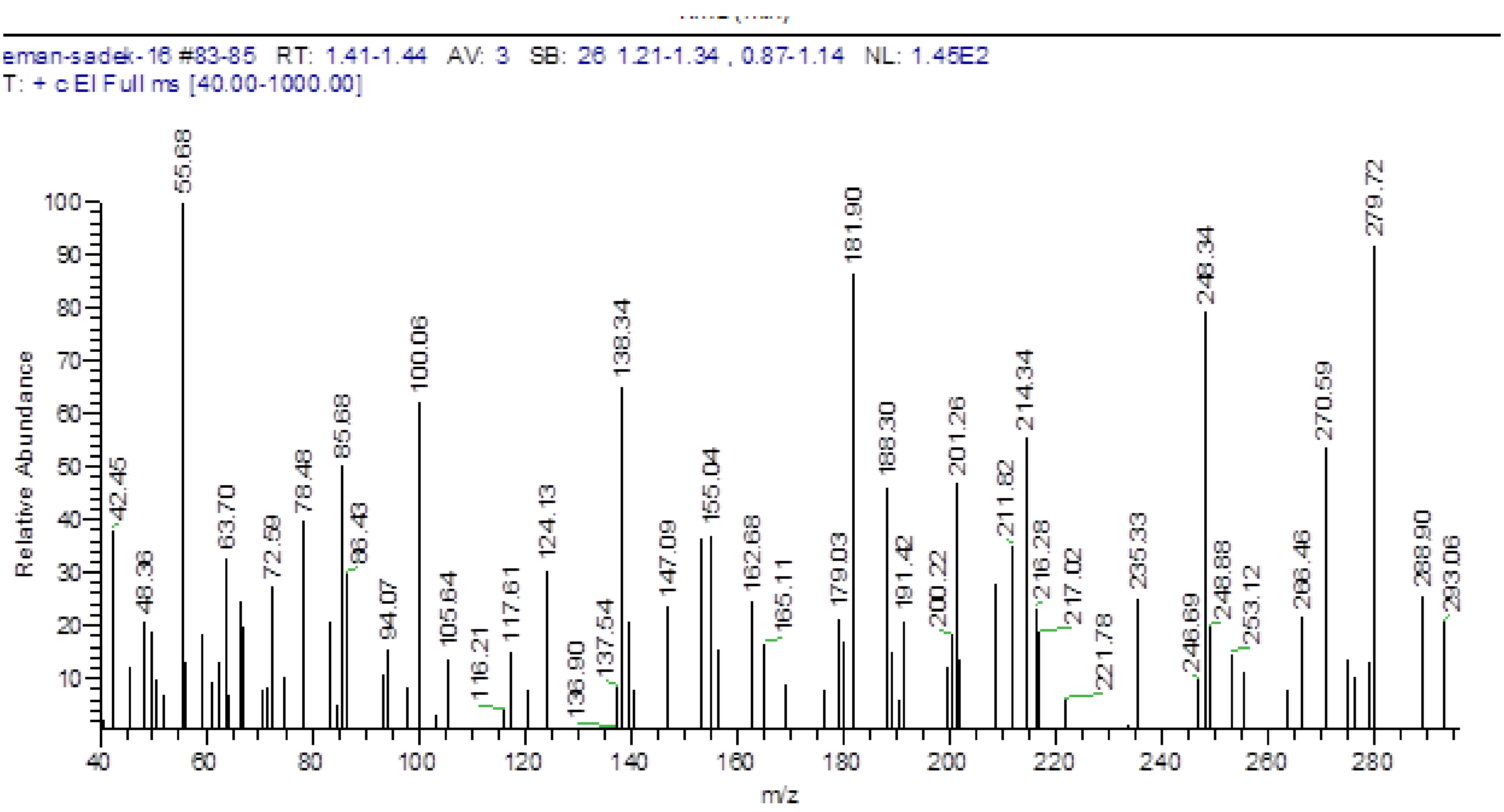

Supplementary fig. (37): mass spectrum for compound **15**.

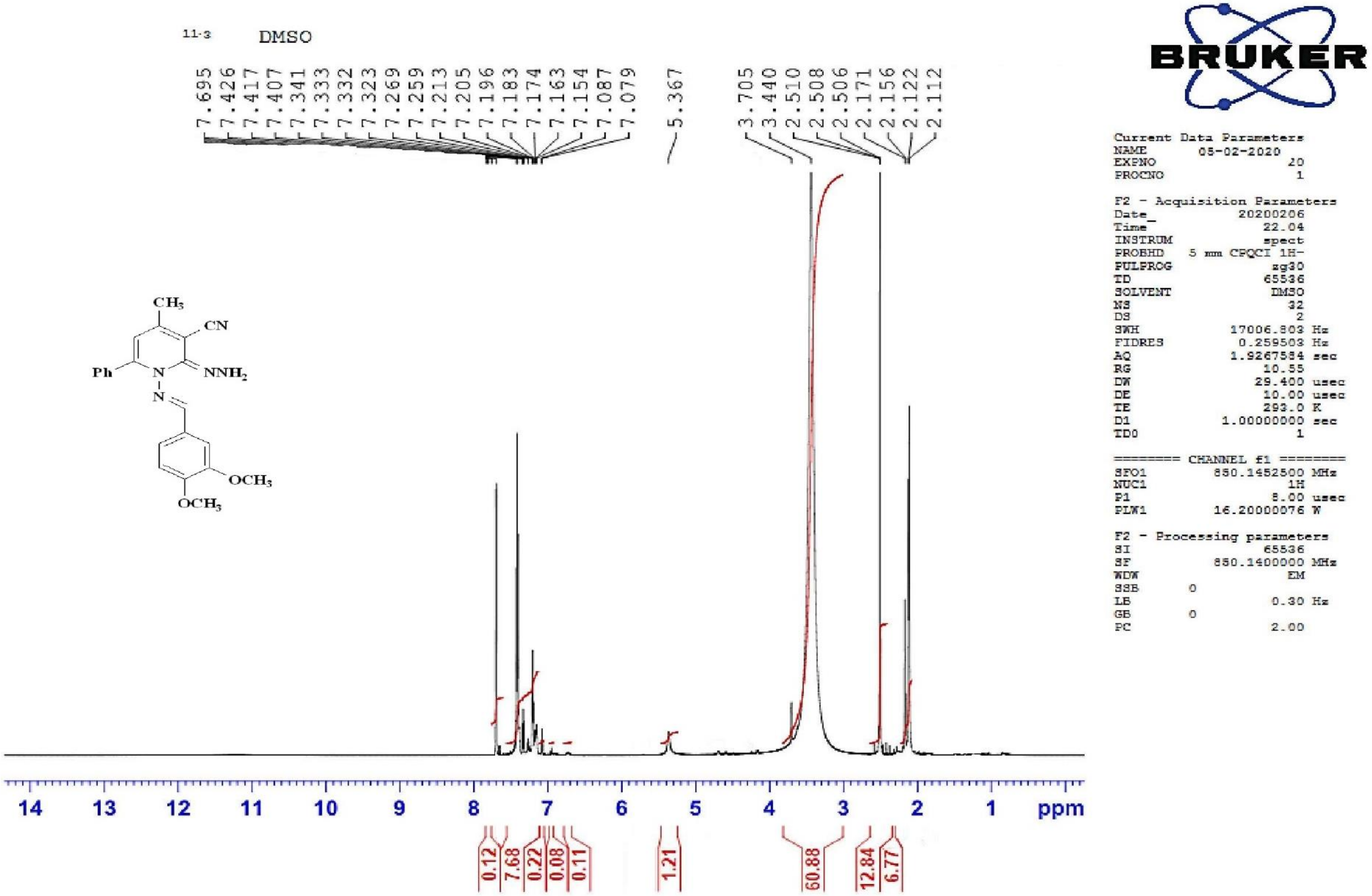

Supplementary fig. (38): ^{1}H NMR for compound **16**.N

NCI anticancer data result

Developmental Therapeutics Program	NSC: D-836918 / 1	Conc: 1.00E-5 Molar	Test Date: Mar 21, 2022
One Dose Mean Graph	Experiment ID: 2203OS92		Report Date: May 15, 2022

Panel/Cell Line	Growth Percent
Leukemia	
CCRF-CEM	121.38
HL-60(TB)	123.04
K-562	106.39
MOLT-4	117.76
RPMI-8226	108.08
SR	108.03
Non-Small Cell Lung Cancer	
A549/ATCC	98.81
EKVX	93.00
HOP-62	83.12
NCI-H226	100.44
NCI-H23	99.36
NCI-H322M	102.40
NCI-H460	111.62
NCI-H522	89.86
Colon Cancer	
COLO 205	109.26
HCC-2998	107.79
HCT-116	96.34
HCT-15	104.05
HT29	93.65
KM12	105.95
SW-620	105.65
CNS Cancer	
SF-268	143.25
SF-295	94.25
SF-539	102.68
SNB-19	91.91
U251	101.83
Melanoma	
LOX IMVI	103.94
MALME-3M	93.27
M14	98.57
MDA-MB-435	101.58
SK-MEL-2	102.05
SK-MEL-28	106.78
SK-MEL-5	102.81
UACC-257	99.76
UACC-62	93.90
Ovarian Cancer	
IGROV1	105.19
OVCAR-3	124.40
OVCAR-4	105.94
OVCAR-5	104.03
OVCAR-8	110.20
NCI/ADR-RES	108.90
SK-OV-3	95.87
Renal Cancer	
786-0	95.55
A498	112.67
ACHN	107.15
CAKI-1	102.17
RXF 393	113.87
SN12C	95.61
TK-10	86.25
UO-31	90.69
Prostate Cancer	
PC-3	113.04
DU-145	108.38
Breast Cancer	
MDA-MB-231/ATCC	100.11
HS 578T	103.04
BT-549	95.69
T-47D	92.40
MDA-MB-468	99.29
Mean	103.39
Delta	20.27
Range	60.13

Mean Growth Percent - Growth Percent

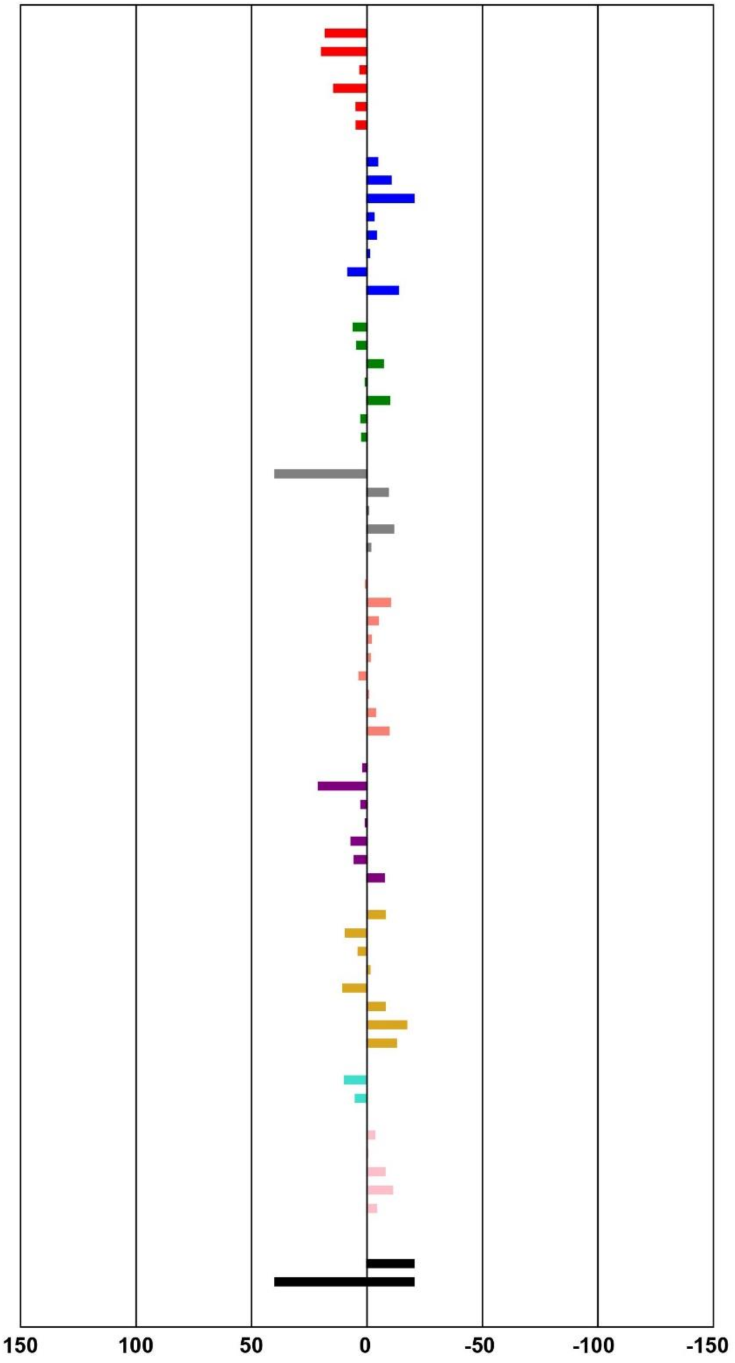

Compound 2

**Developmental Therapeutics Program**	**NSC:** D-836922 / 1	**Conc:** 1.00E-5 Molar	**Test Date:** Mar 21, 2022
**One Dose Mean Graph**	**Experiment ID:** 2203OS92		**Report Date:** May 07, 2022

Panel/Cell Line	Growth Percent
Leukemia	
CCRF-CEM	109.83
HL-60(TB)	110.25
K-562	87.50
MOLT-4	101.95
RPMI-8226	77.55
SR	92.73
Non-Small Cell Lung Cancer	
A549/ATCC	97.94
EKVX	113.33
HOP-62	76.62
NCI-H226	91.28
NCI-H23	98.57
NCI-H322M	105.33
NCI-H460	98.54
NCI-H522	80.95
Colon Cancer	
COLO 205	104.07
HCC-2998	107.68
HCT-116	90.15
HCT-15	108.43
HT29	97.29
KM12	96.39
SW-620	104.17
CNS Cancer	
SF-268	102.96
SF-295	98.41
SF-539	103.65
SNB-19	89.60
U251	103.77
Melanoma	
LOX IMVI	104.70
MALME-3M	96.60
M14	100.80
MDA-MB-435	105.72
SK-MEL-2	99.25
SK-MEL-28	105.61
SK-MEL-5	95.96
UACC-257	100.78
UACC-62	78.29
Ovarian Cancer	
IGROV1	84.91
OVCAR-3	105.02
OVCAR-4	90.04
OVCAR-5	112.80
OVCAR-8	103.79
NCI/ADR-RES	104.48
SK-OV-3	91.47
Renal Cancer	
786-0	103.74
A498	125.00
ACHN	91.84
CAKI-1	83.79
RXF 393	111.38
SN12C	93.78
TK-10	144.02
UO-31	67.93
Prostate Cancer	
PC-3	91.17
DU-145	101.46
Breast Cancer	
MDA-MB-231/ATCC	86.92
HS 578T	104.35
BT-549	96.14
T-47D	67.70
MDA-MB-468	97.76
Mean	98.18
Delta	30.48
Range	76.32

Mean Growth Percent - Growth Percent

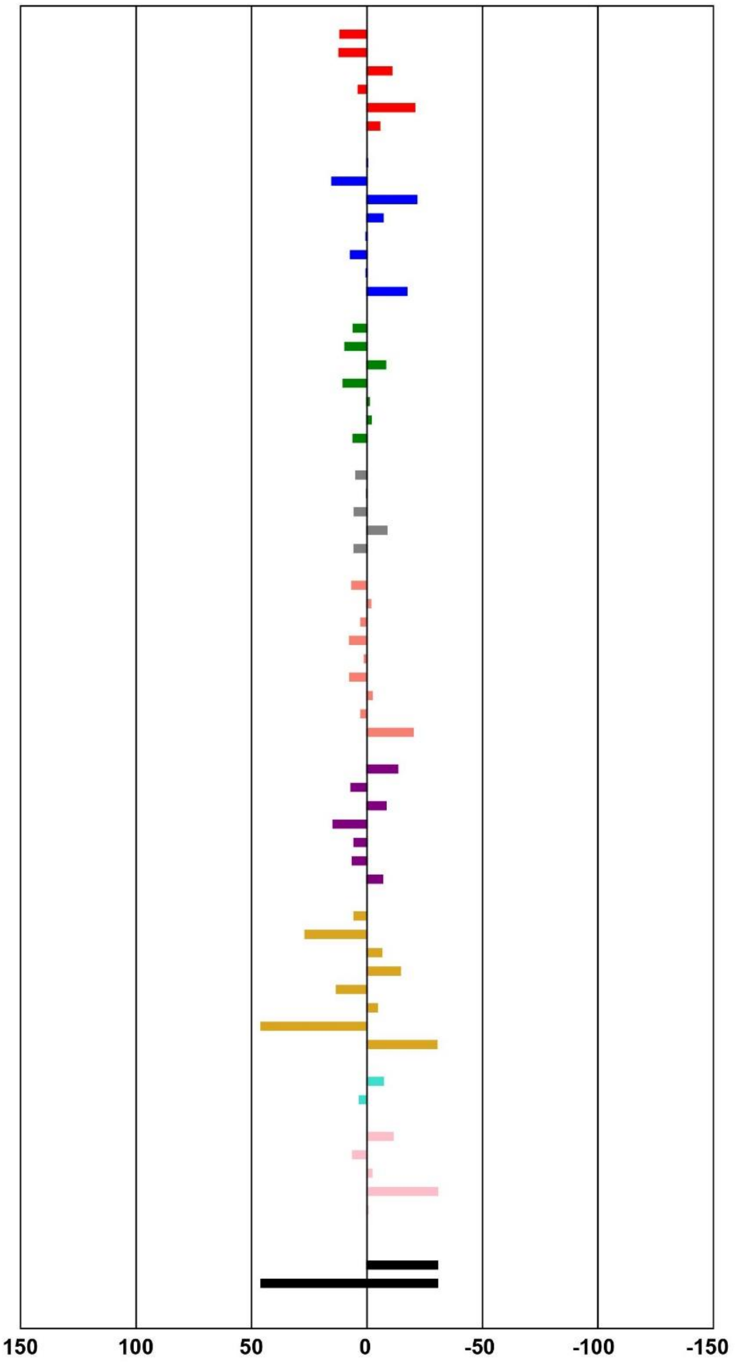

Compound 4

Developmental Therapeutics Program	NSC: D-836919 / 1	Conc: 1.00E-5 Molar	Test Date: Mar 21, 2022
One Dose Mean Graph	Experiment ID: 2203OS92		Report Date: May 15, 2022

Panel/Cell Line	Growth Percent
Leukemia	
CCRF-CEM	111.24
HL-60(TB)	119.51
K-562	93.83
MOLT-4	118.70
RPMI-8226	86.57
SR	107.14
Non-Small Cell Lung Cancer	
A549/ATCC	99.26
EKVX	99.90
HOP-62	87.12
NCI-H226	99.83
NCI-H23	97.88
NCI-H322M	102.05
NCI-H460	110.61
NCI-H522	86.68
Colon Cancer	
COLO 205	112.07
HCC-2998	119.38
HCT-116	94.33
HCT-15	108.89
HT29	103.57
KM12	102.29
SW-620	103.75
CNS Cancer	
SF-268	115.72
SF-295	98.46
SF-539	107.67
SNB-19	91.13
U251	102.82
Melanoma	
LOX IMVI	104.30
MALME-3M	90.59
M14	96.35
MDA-MB-435	118.36
SK-MEL-2	100.67
SK-MEL-28	108.40
SK-MEL-5	103.30
UACC-257	94.27
UACC-62	93.99
Ovarian Cancer	
IGROV1	103.93
OVCAR-3	100.22
OVCAR-4	99.96
OVCAR-5	100.66
OVCAR-8	112.73
NCI/ADR-RES	112.28
SK-OV-3	96.56
Renal Cancer	
786-0	102.62
A498	128.08
ACHN	102.87
CAKI-1	98.12
RXF 393	107.36
SN12C	92.58
TK-10	124.98
UO-31	75.26
Prostate Cancer	
PC-3	103.13
DU-145	112.50
Breast Cancer	
MDA-MB-231/ATCC	95.16
HS 578T	97.10
BT-549	97.21
T-47D	90.25
MDA-MB-468	113.25
Mean	102.76
Delta	27.50
Range	52.82

Mean Growth Percent - Growth Percent

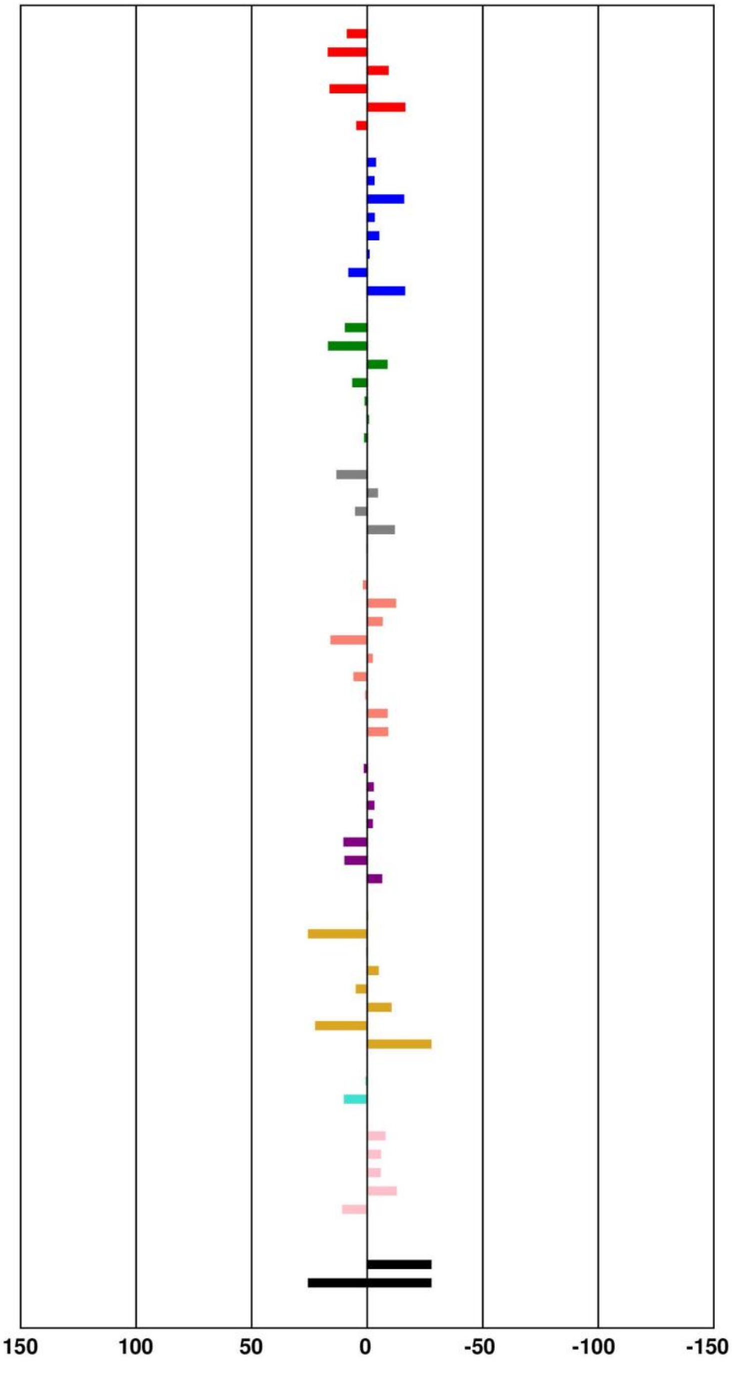

Compound 5

Developmental Therapeutics Program<br>One Dose Mean Graph	NSC: D-836923 / 1	Conc: 1.00E-5 Molar	Test Date: Mar 21, 2022
	Experiment ID: 2203OS92		Report Date: May 07, 2022

Panel/Cell Line	Growth Percent
Leukemia	
CCRF-CEM	108.39
HL-60(TB)	133.72
K-562	80.94
MOLT-4	104.46
RPMI-8226	88.57
SR	90.70
Non-Small Cell Lung Cancer	
A549/ATCC	104.15
EKVX	103.92
HOP-62	69.81
NCI-H226	94.23
NCI-H23	101.38
NCI-H322M	91.91
NCI-H460	105.52
NCI-H522	83.81
Colon Cancer	
COLO 205	122.77
HCC-2998	115.55
HCT-116	95.86
HCT-15	102.39
HT29	97.14
KM12	101.95
SW-620	108.87
CNS Cancer	
SF-268	103.56
SF-295	105.61
SF-539	106.83
SNB-19	91.06
U251	98.68
Melanoma	
LOX IMVI	91.32
MALME-3M	101.89
M14	102.21
MDA-MB-435	107.34
SK-MEL-2	95.83
SK-MEL-28	116.10
SK-MEL-5	101.74
UACC-257	103.69
UACC-62	77.18
Ovarian Cancer	
IGROV1	100.28
OVCAR-3	110.56
OVCAR-4	95.38
OVCAR-5	109.67
OVCAR-8	107.49
NCI/ADR-RES	109.25
SK-OV-3	88.02
Renal Cancer	
786-0	112.73
A498	141.98
ACHN	100.87
CAKI-1	83.09
RXF 393	122.04
SN12C	95.61
TK-10	166.45
UO-31	71.64
Prostate Cancer	
PC-3	78.98
DU-145	107.22
Breast Cancer	
MDA-MB-231/ATCC	84.57
HS 578T	112.75
BT-549	96.98
T-47D	71.69
MDA-MB-468	108.74
Mean	101.49
Delta	31.68
Range	96.64

Mean Growth Percent - Growth Percent

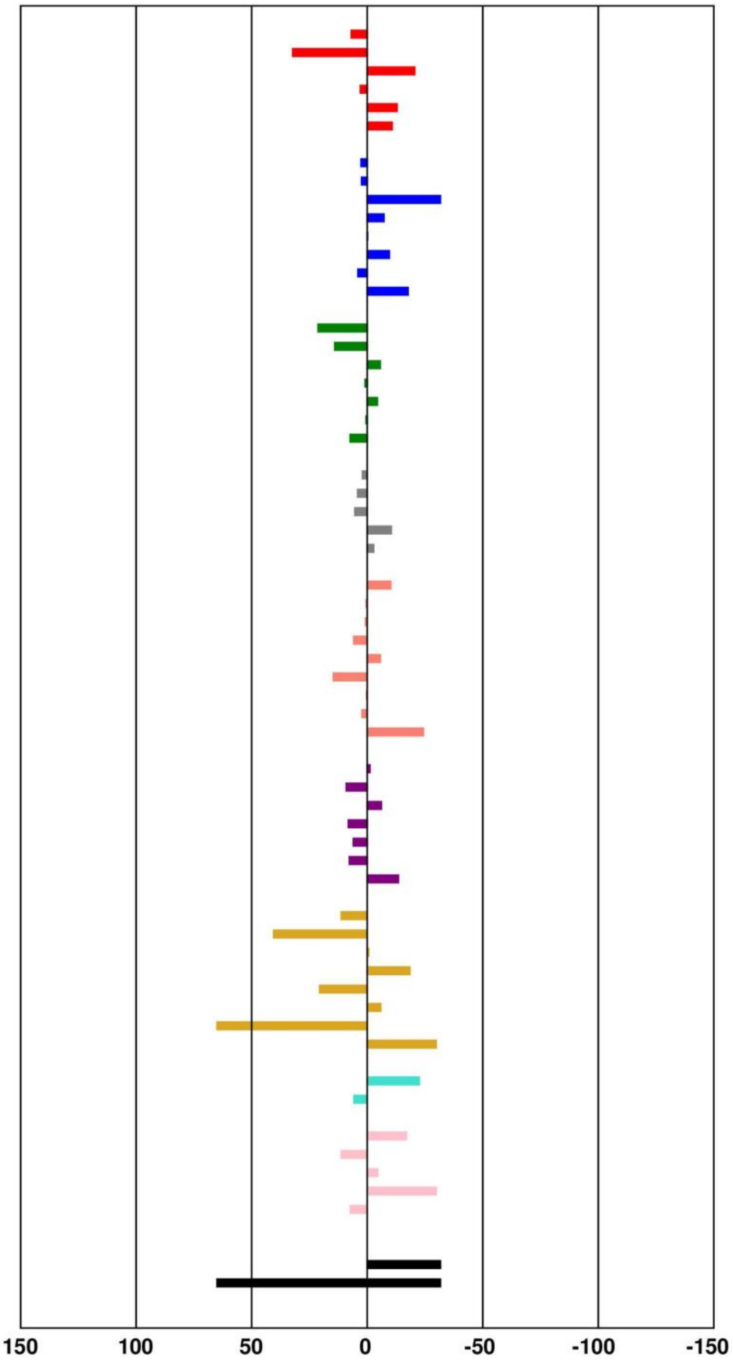

Compound 7

Developmental Therapeutics Program	NSC: D-836924 / 1	Conc: 1.00E-5 Molar	Test Date: Mar 21, 2022
One Dose Mean Graph	Experiment ID: 2203OS92		Report Date: May 07, 2022

Panel/Cell Line	Growth Percent
**Leukemia**	
CCRF-CEM	108.61
HL-60(TB)	126.30
K-562	88.53
MOLT-4	102.46
RPMI-8226	70.58
SR	93.78
**Non-Small Cell Lung Cancer**	
A549/ATCC	99.93
EKVX	98.71
HOP-62	70.61
NCI-H226	93.72
NCI-H23	93.43
NCI-H322M	93.53
NCI-H460	102.05
NCI-H522	75.86
**Colon Cancer**	
COLO 205	99.08
HCC-2998	104.95
HCT-116	85.29
HCT-15	100.30
HT29	95.84
KM12	89.58
SW-620	99.09
**CNS Cancer**	
SF-268	99.36
SF-295	85.09
SF-539	104.99
SNB-19	85.60
U251	100.71
**Melanoma**	
LOX IMVI	96.99
MALME-3M	85.24
M14	92.80
MDA-MB-435	99.13
SK-MEL-2	93.93
SK-MEL-28	105.51
SK-MEL-5	95.24
UACC-257	90.03
UACC-62	71.43
**Ovarian Cancer**	
IGROV1	100.99
OVCAR-3	98.25
OVCAR-4	86.90
OVCAR-5	103.55
OVCAR-8	100.39
NCI/ADR-RES	98.25
SK-OV-3	63.75
**Renal Cancer**	
786-0	107.53
A498	122.84
ACHN	93.05
CAKI-1	83.61
RXF 393	118.93
SN12C	94.01
TK-10	143.58
UO-31	75.90
**Prostate Cancer**	
PC-3	76.72
DU-145	105.63
**Breast Cancer**	
MDA-MB-231/ATCC	86.22
HS 578T	91.86
BT-549	95.21
T-47D	81.39
MDA-MB-468	104.84
Mean	95.29
Delta	31.54
Range	79.83

Mean Growth Percent - Growth Percent

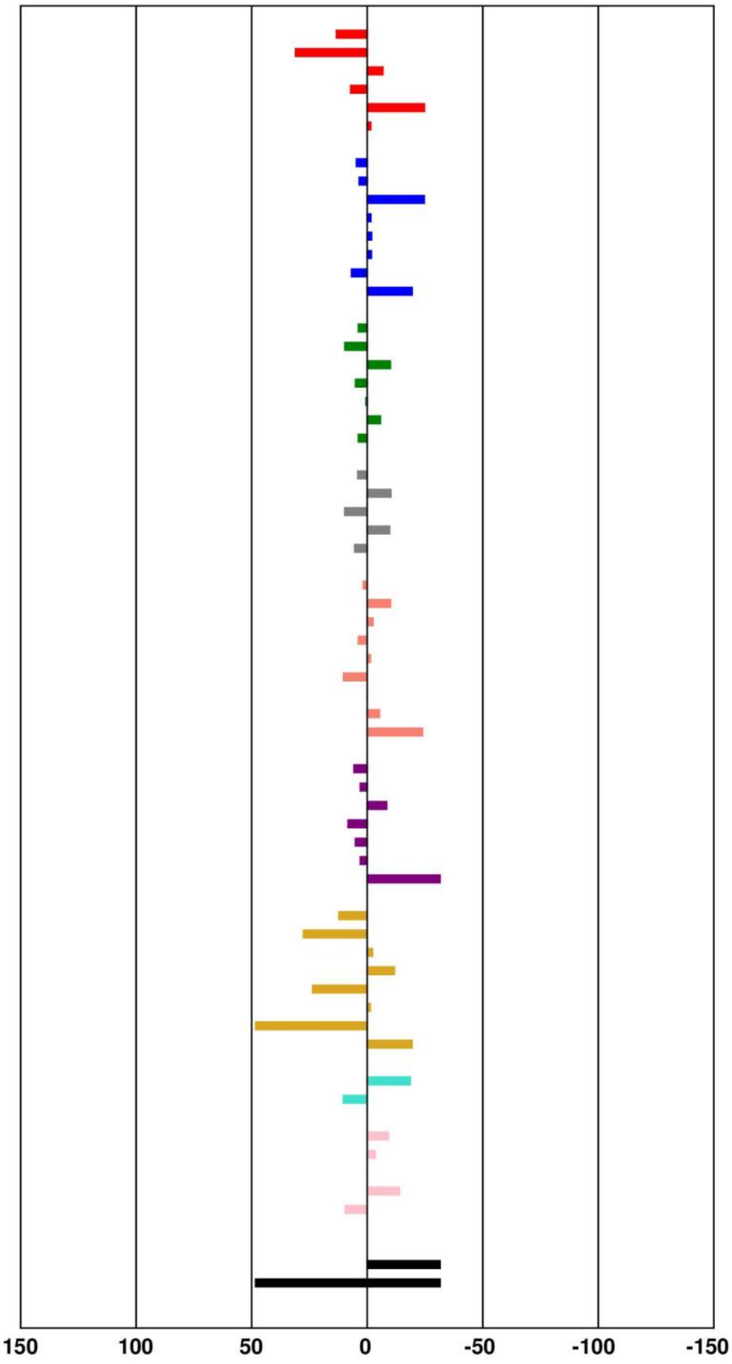

Compound 8

Developmental Therapeutics Program	NSC: D-836925 / 1	Conc: 1.00E-5 Molar	Test Date: Mar 21, 2022
One Dose Mean Graph	Experiment ID: 2203OS92		Report Date: May 07, 2022

Panel/Cell Line	Growth Percent
Leukemia	
CCRF-CEM	116.13
HL-60(TB)	117.89
K-562	98.09
MOLT-4	103.06
RPMI-8226	48.42
SR	104.43
Non-Small Cell Lung Cancer	
A549/ATCC	93.67
EKVX	100.71
HOP-62	79.83
NCI-H226	88.42
NCI-H23	94.91
NCI-H322M	94.71
NCI-H460	100.58
NCI-H522	87.45
Colon Cancer	
COLO 205	105.17
HCC-2998	115.79
HCT-116	22.06
HCT-15	98.65
HT29	93.86
KM12	102.57
SW-620	91.93
CNS Cancer	
SF-268	101.50
SF-295	90.43
SF-539	98.62
SNB-19	87.21
U251	90.63
Melanoma	
LOX IMVI	91.24
MALME-3M	92.21
M14	94.49
MDA-MB-435	101.10
SK-MEL-2	103.32
SK-MEL-28	104.88
SK-MEL-5	100.00
UACC-257	94.90
UACC-62	80.44
Ovarian Cancer	
IGROV1	89.38
OVCAR-3	110.96
OVCAR-4	101.17
OVCAR-5	96.45
OVCAR-8	100.22
NCI/ADR-RES	99.96
SK-OV-3	87.32
Renal Cancer	
786-0	106.20
A498	141.83
ACHN	94.38
CAKI-1	83.23
RXF 393	94.29
SN12C	94.10
TK-10	127.22
UO-31	63.16
Prostate Cancer	
PC-3	90.09
DU-145	109.36
Breast Cancer	
MDA-MB-231/ATCC	84.65
HS 578T	90.73
BT-549	92.59
T-47D	80.63
MDA-MB-468	114.25
Mean	95.46
Delta	73.40
Range	119.77

Mean Growth Percent - Growth Percent

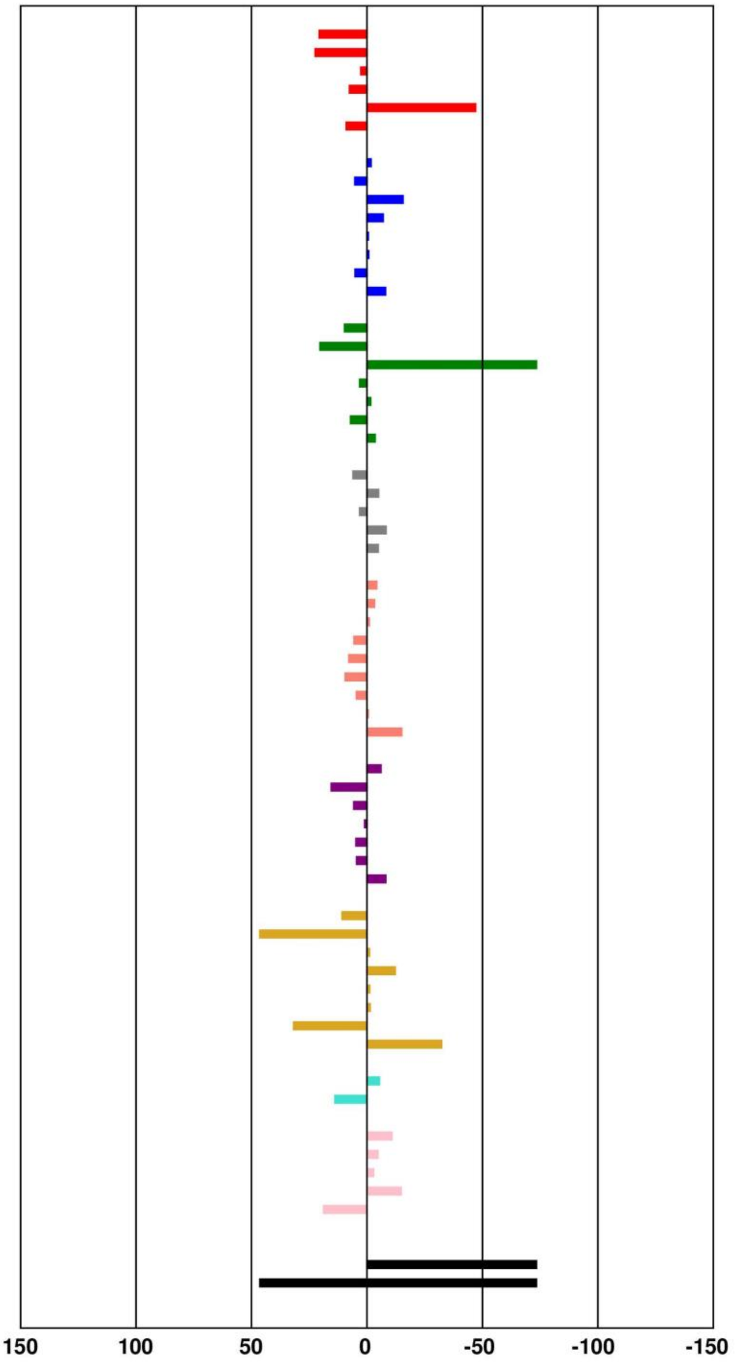

Compound 9

**Developmental Therapeutics Program**	**NSC:** D-836926 / 1	**Conc:** 1.00E-5 Molar	**Test Date:** Mar 21, 2022
**One Dose Mean Graph**	**Experiment ID:** 2203OS92		**Report Date:** May 07, 2022

**Panel/Cell Line**	**Growth Percent**
Leukemia	
CCRF-CEM	118.09
HL-60(TB)	141.38
K-562	100.56
MOLT-4	123.37
RPMI-8226	103.11
SR	108.43
Non-Small Cell Lung Cancer	
A549/ATCC	101.07
EKVX	83.61
HOP-62	77.72
NCI-H226	92.64
NCI-H23	94.47
NCI-H322M	97.85
NCI-H460	107.20
NCI-H522	91.17
Colon Cancer	
COLO 205	108.43
HCC-2998	111.94
HCT-116	92.65
HCT-15	104.42
HT29	100.27
KM12	104.62
SW-620	95.86
CNS Cancer	
SF-268	129.91
SF-295	94.77
SF-539	92.14
SNB-19	94.26
U251	95.06
Melanoma	
LOX IMVI	95.75
MALME-3M	83.70
M14	94.14
MDA-MB-435	107.18
SK-MEL-2	104.02
SK-MEL-28	105.09
SK-MEL-5	100.37
UACC-257	95.19
UACC-62	87.27
Ovarian Cancer	
IGROV1	98.81
OVCAR-3	111.78
OVCAR-4	96.38
OVCAR-5	100.35
OVCAR-8	104.74
NCI/ADR-RES	101.84
SK-OV-3	86.58
Renal Cancer	
786-0	107.14
A498	126.88
ACHN	95.40
CAKI-1	86.59
RXF 393	114.85
SN12C	97.82
TK-10	130.79
UO-31	65.52
Prostate Cancer	
PC-3	95.01
DU-145	111.29
Breast Cancer	
MDA-MB-231/ATCC	82.99
HS 578T	86.84
BT-549	92.57
T-47D	87.91
MDA-MB-468	114.21
Mean	100.60
Delta	35.08
Range	75.86

Mean Growth Percent - Growth Percent

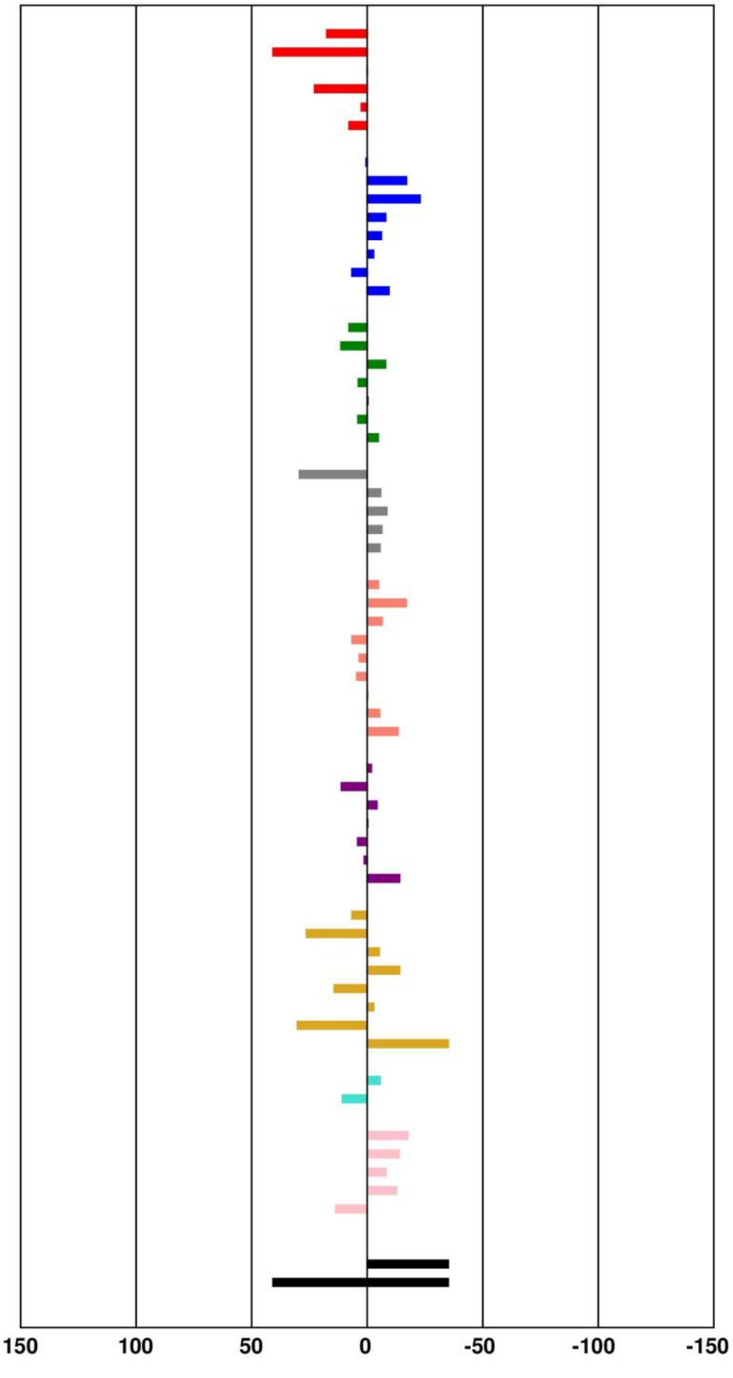

Compound 10

Developmental Therapeutics Program	NSC: D-836927 / 1	Conc: 1.00E-5 Molar	Test Date: Mar 21, 2022
One Dose Mean Graph	Experiment ID: 2203OS92		Report Date: May 07, 2022

Panel/Cell Line	Growth Percent
Leukemia	
CCRF-CEM	116.69
HL-60(TB)	149.67
K-562	102.85
MOLT-4	124.20
RPMI-8226	103.44
SR	107.34
Non-Small Cell Lung Cancer	
A549/ATCC	104.60
EKVX	102.72
HOP-62	81.43
NCI-H226	101.89
NCI-H23	98.54
NCI-H322M	100.69
NCI-H460	114.42
NCI-H522	89.37
Colon Cancer	
COLO 205	107.82
HCC-2998	113.26
HCT-116	100.37
HCT-15	104.16
HT29	107.09
KM12	106.47
SW-620	99.95
CNS Cancer	
SF-268	133.77
SF-295	93.64
SF-539	99.93
SNB-19	95.14
U251	100.86
Melanoma	
LOX IMVI	103.47
MALME-3M	94.67
M14	99.01
MDA-MB-435	111.92
SK-MEL-2	117.97
SK-MEL-28	115.57
SK-MEL-5	104.90
UACC-257	95.74
UACC-62	94.61
Ovarian Cancer	
IGROV1	99.81
OVCAR-3	102.66
OVCAR-4	102.09
OVCAR-5	105.37
OVCAR-8	110.23
NCI/ADR-RES	106.62
SK-OV-3	89.64
Renal Cancer	
786-0	109.64
A498	144.38
ACHN	100.18
CAKI-1	92.25
RXF 393	123.52
SN12C	101.04
TK-10	141.13
UO-31	74.70
Prostate Cancer	
PC-3	96.71
DU-145	110.17
Breast Cancer	
MDA-MB-231/ATCC	94.83
HS 578T	99.33
BT-549	99.95
T-47D	101.79
MDA-MB-468	120.87
Mean	105.70
Delta	31.00
Range	74.97

Mean Growth Percent - Growth Percent

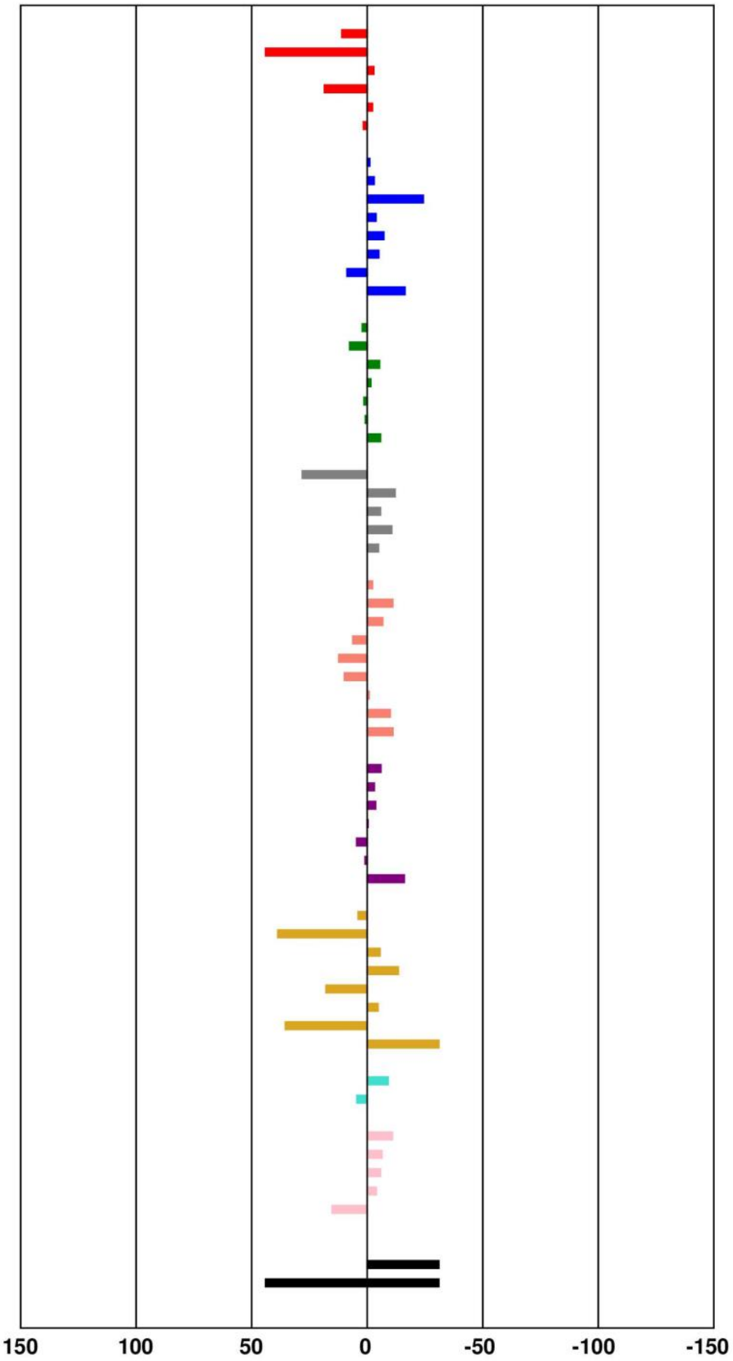

Compound 12

Developmental Therapeutics Program	NSC: D-836920 / 1	Conc: 1.00E-5 Molar	Test Date: Mar 21, 2022
One Dose Mean Graph	Experiment ID: 2203OS92		Report Date: May 15, 2022

Panel/Cell Line	Growth Percent
Leukemia	
CCRF-CEM	113.83
HL-60(TB)	125.28
K-562	104.95
MOLT-4	112.58
RPMI-8226	103.85
SR	108.48
Non-Small Cell Lung Cancer	
A549/ATCC	109.41
EKVX	98.64
HOP-62	86.90
NCI-H226	106.63
NCI-H23	107.42
NCI-H322M	104.99
NCI-H460	112.83
NCI-H522	100.17
Colon Cancer	
COLO 205	110.55
HCC-2998	124.97
HCT-116	97.70
HCT-15	102.55
HT29	94.52
KM12	105.09
SW-620	102.00
CNS Cancer	
SF-268	113.95
SF-295	103.65
SF-539	101.05
SNB-19	93.88
U251	104.02
Melanoma	
LOX IMVI	106.72
MALME-3M	93.23
M14	93.36
MDA-MB-435	114.63
SK-MEL-2	113.93
SK-MEL-28	112.34
SK-MEL-5	108.08
UACC-257	103.96
UACC-62	96.00
Ovarian Cancer	
IGROV1	113.04
OVCAR-3	114.20
OVCAR-4	108.71
OVCAR-5	110.86
OVCAR-8	107.40
NCI/ADR-RES	112.58
SK-OV-3	96.10
Renal Cancer	
786-0	103.67
A498	128.12
ACHN	103.22
CAKI-1	99.72
RXF 393	115.74
SN12C	103.95
TK-10	109.00
UO-31	89.22
Prostate Cancer	
PC-3	111.60
DU-145	111.69
Breast Cancer	
MDA-MB-231/ATCC	101.48
HS 578T	107.87
BT-549	98.76
T-47D	102.79
MDA-MB-468	115.06
Mean	106.26
Delta	19.36
Range	41.22

Mean Growth Percent - Growth Percent

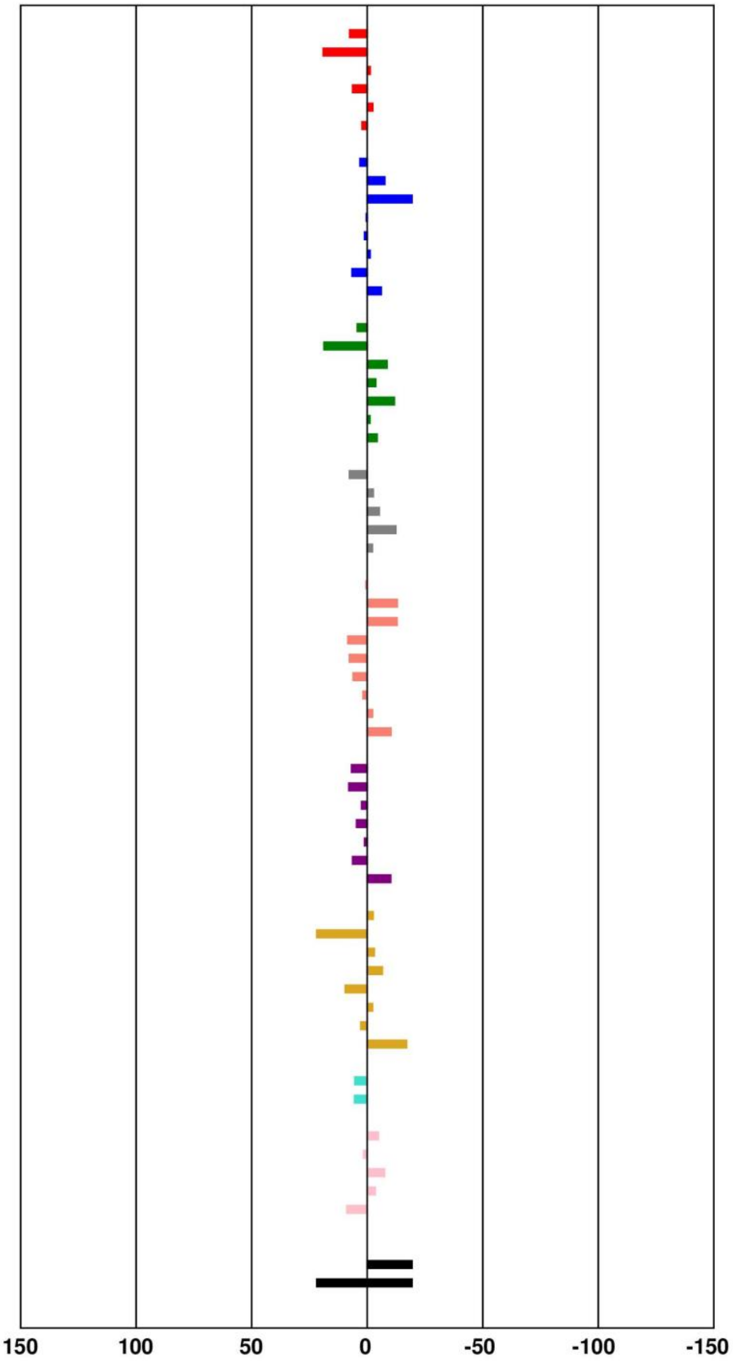

Compound 13

Developmental Therapeutics Program	NSC: D-836928 / 1	Conc: 1.00E-5 Molar	Test Date: Mar 21, 2022
One Dose Mean Graph	Experiment ID: 2203OS92		Report Date: May 07, 2022

Panel/Cell Line	Growth Percent
Leukemia	
CCRF-CEM	109.97
HL-60(TB)	137.68
K-562	109.94
MOLT-4	114.25
RPMI-8226	110.01
SR	96.04
Non-Small Cell Lung Cancer	
A549/ATCC	99.33
EKVX	99.74
HOP-62	85.57
NCI-H226	111.06
NCI-H23	104.55
NCI-H322M	104.90
NCI-H460	111.23
NCI-H522	93.75
Colon Cancer	
COLO 205	113.49
HCC-2998	117.56
HCT-116	101.36
HCT-15	103.91
HT29	113.96
KM12	106.63
SW-620	98.03
CNS Cancer	
SF-268	133.92
SF-295	103.36
SF-539	102.37
SNB-19	97.26
U251	96.23
Melanoma	
LOX IMVI	108.91
MALME-3M	93.32
M14	103.20
MDA-MB-435	106.42
SK-MEL-2	113.10
SK-MEL-28	109.20
SK-MEL-5	105.98
UACC-257	96.60
UACC-62	95.98
Ovarian Cancer	
IGROV1	108.57
OVCAR-3	111.38
OVCAR-4	111.84
OVCAR-5	113.57
OVCAR-8	110.63
NCI/ADR-RES	111.88
SK-OV-3	101.11
Renal Cancer	
786-0	98.24
A498	131.98
ACHN	109.84
CAKI-1	99.81
RXF 393	131.17
SN12C	105.50
TK-10	102.68
UO-31	91.24
Prostate Cancer	
PC-3	106.07
DU-145	110.60
Breast Cancer	
MDA-MB-231/ATCC	105.45
HS 578T	107.10
BT-549	103.75
T-47D	98.83
MDA-MB-468	124.45
Mean	107.10
Delta	21.53
Range	52.11

Mean Growth Percent - Growth Percent

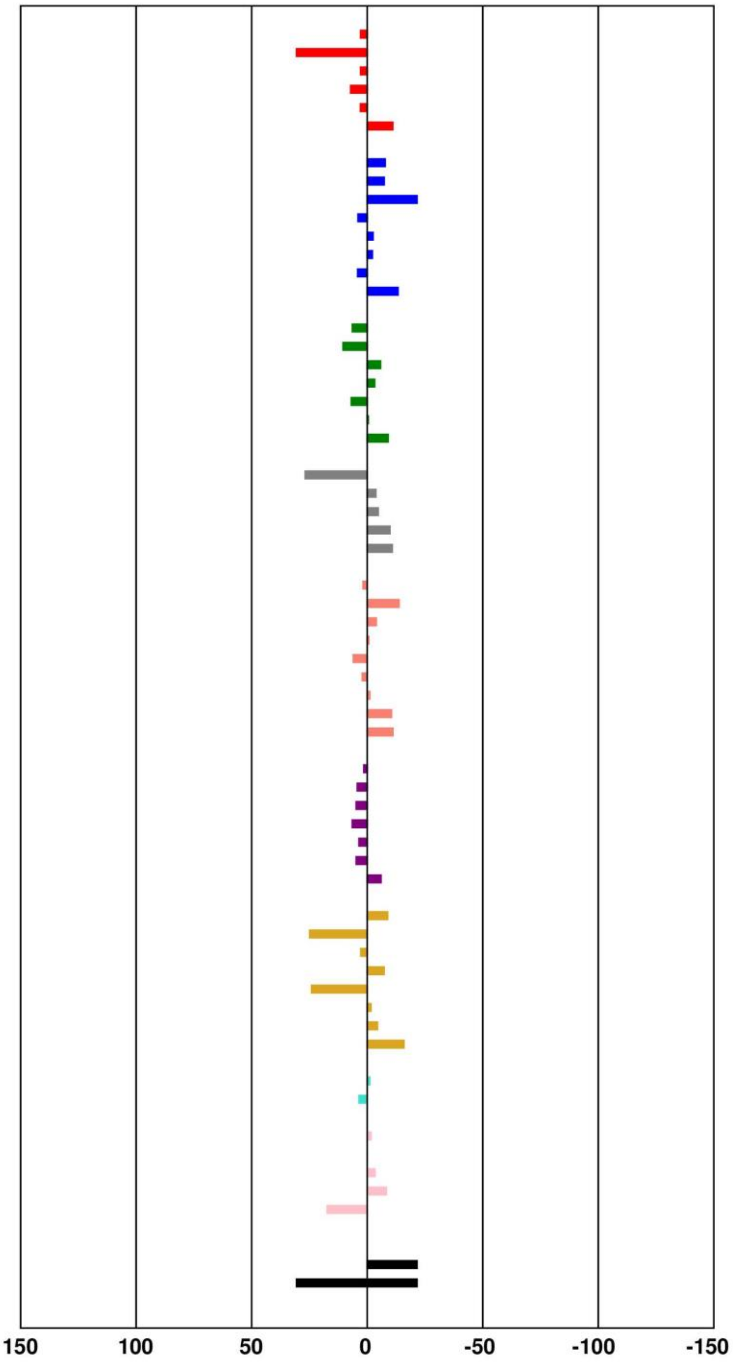

Compound 14

Developmental Therapeutics Program	NSC: D-836929 / 1	Conc: 1.00E-5 Molar	Test Date: Mar 21, 2022
One Dose Mean Graph	Experiment ID: 2203OS92		Report Date: May 07, 2022

Panel/Cell Line	Growth Percent
Leukemia	
CCRF-CEM	112.43
HL-60(TB)	119.08
K-562	106.09
MOLT-4	125.17
RPMI-8226	117.18
SR	94.59
Non-Small Cell Lung Cancer	
A549/ATCC	99.23
EKVX	95.20
HOP-62	83.84
NCI-H226	114.82
NCI-H23	105.48
NCI-H322M	106.63
NCI-H460	111.70
NCI-H522	95.69
Colon Cancer	
COLO 205	112.11
HCC-2998	120.95
HCT-116	97.17
HCT-15	105.48
HT29	107.65
KM12	101.88
SW-620	100.40
CNS Cancer	
SF-268	113.50
SF-295	96.61
SF-539	102.63
SNB-19	97.53
U251	99.14
Melanoma	
LOX IMVI	107.88
MALME-3M	95.15
M14	102.38
MDA-MB-435	102.19
SK-MEL-2	110.73
SK-MEL-28	105.12
SK-MEL-5	106.72
UACC-257	94.34
UACC-62	93.42
Ovarian Cancer	
IGROV1	112.12
OVCAR-3	111.45
OVCAR-4	103.15
OVCAR-5	114.19
OVCAR-8	107.80
NCI/ADR-RES	111.21
SK-OV-3	104.25
Renal Cancer	
786-0	97.73
A498	131.82
ACHN	105.85
CAKI-1	97.32
RXF 393	137.65
SN12C	106.00
TK-10	90.89
UO-31	90.17
Prostate Cancer	
PC-3	98.92
DU-145	101.59
Breast Cancer	
MDA-MB-231/ATCC	104.06
HS 578T	100.06
BT-549	105.20
T-47D	101.14
MDA-MB-468	130.36
Mean	105.67
Delta	21.83
Range	53.81

Mean Growth Percent - Growth Percent

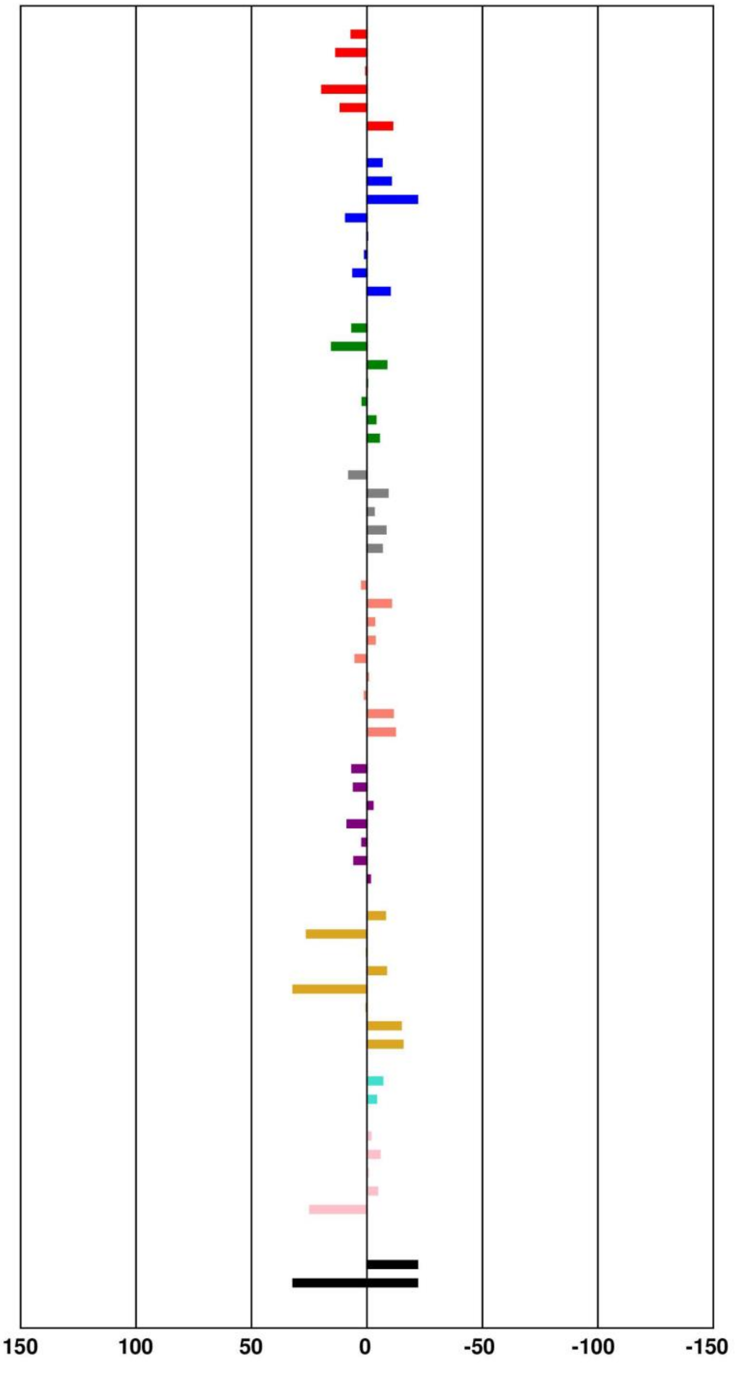

Compound 15

Developmental Therapeutics Program	NSC: D-836930 / 1	Conc: 1.00E-5 Molar	Test Date: Mar 21, 2022
One Dose Mean Graph	Experiment ID: 2203OS92		Report Date: May 07, 2022

Panel/Cell Line	Growth Percent
Leukemia	
CCRF-CEM	92.50
HL-60(TB)	121.16
K-562	96.93
MOLT-4	112.24
RPMI-8226	102.69
SR	103.00
Non-Small Cell Lung Cancer	
A549/ATCC	100.21
EKVX	104.72
HOP-62	77.20
NCI-H226	99.61
NCI-H23	103.61
NCI-H322M	102.35
NCI-H460	107.30
NCI-H522	102.73
Colon Cancer	
COLO 205	110.20
HCC-2998	110.79
HCT-116	92.50
HCT-15	106.97
HT29	112.44
KM12	97.27
SW-620	104.22
CNS Cancer	
SF-268	102.99
SF-295	95.24
SF-539	94.57
SNB-19	94.26
U251	96.64
Melanoma	
LOX IMVI	101.21
MALME-3M	91.31
M14	98.07
MDA-MB-435	105.36
SK-MEL-2	104.85
SK-MEL-28	110.64
SK-MEL-5	103.34
UACC-257	101.49
UACC-62	90.36
Ovarian Cancer	
IGROV1	95.64
OVCAR-3	109.78
OVCAR-4	92.35
OVCAR-5	111.36
OVCAR-8	108.81
NCI/ADR-RES	109.80
SK-OV-3	82.12
Renal Cancer	
786-0	101.87
A498	131.64
ACHN	92.77
CAKI-1	84.43
RXF 393	156.69
SN12C	96.97
TK-10	138.09
UO-31	72.29
Prostate Cancer	
PC-3	81.86
DU-145	102.06
Breast Cancer	
MDA-MB-231/ATCC	89.89
HS 578T	101.30
BT-549	88.78
T-47D	84.98
MDA-MB-468	125.28
Mean	101.93
Delta	29.64
Range	84.40

Mean Growth Percent - Growth Percent

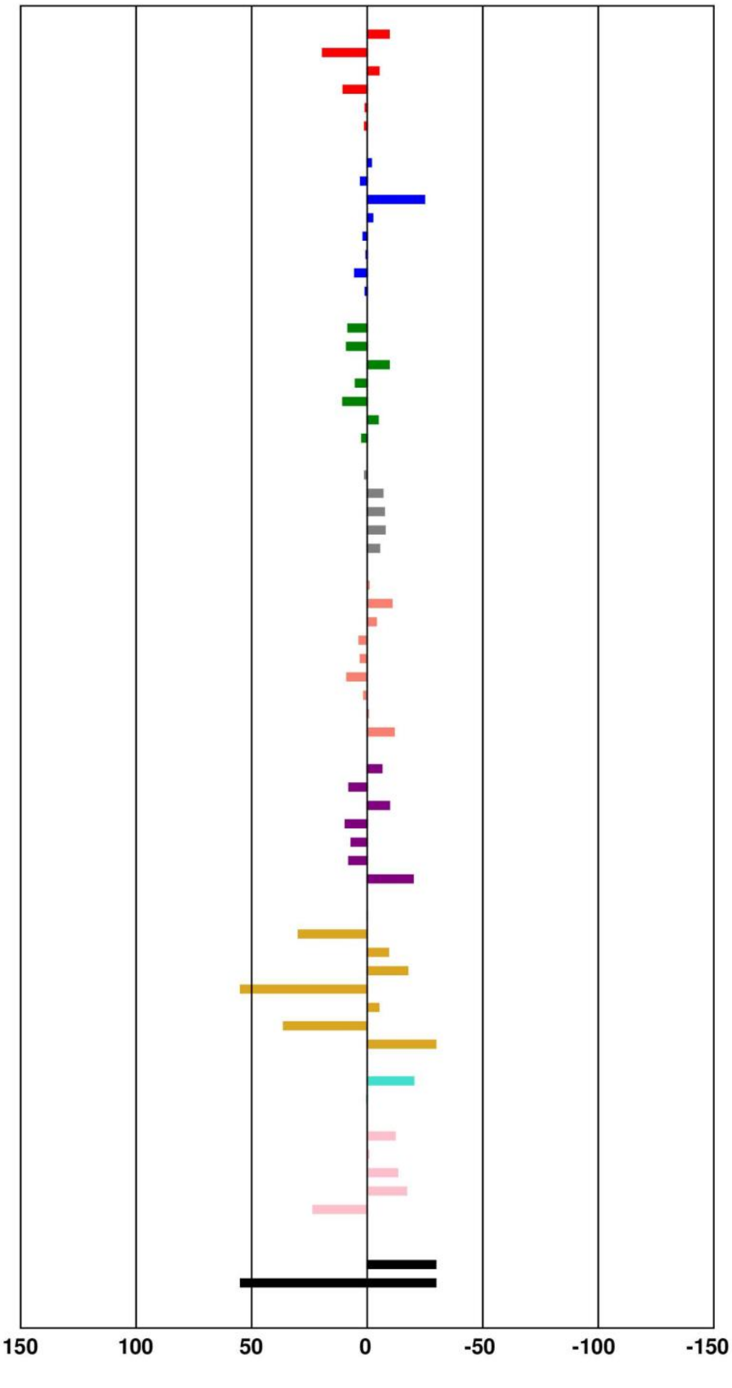

Compound 16
